# Remdesivir and three other drugs for hospitalised patients with COVID-19: final results of the WHO Solidarity randomised trial and updated meta-analyses

**DOI:** 10.1016/S0140-6736(22)00519-0

**Published:** 2022-05-21

**Authors:** WHO Solidarity Trial Consortium

## Abstract

**Background:**

The Solidarity trial among COVID-19 inpatients has previously reported interim mortality analyses for four repurposed antiviral drugs. Lopinavir, hydroxychloroquine, and interferon (IFN)-β1a were discontinued for futility but randomisation to remdesivir continued. Here, we report the final results of Solidarity and meta-analyses of mortality in all relevant trials to date.

**Methods:**

Solidarity enrolled consenting adults (aged ≥18 years) recently hospitalised with, in the view of their doctor, definite COVID-19 and no contraindication to any of the study drugs, regardless of any other patient characteristics. Participants were randomly allocated, in equal proportions between the locally available options, to receive whichever of the four study drugs (lopinavir, hydroxychloroquine, IFN-β1a, or remdesivir) were locally available at that time or no study drug (controls). All patients also received the local standard of care. No placebos were given. The protocol-specified primary endpoint was in-hospital mortality, subdivided by disease severity. Secondary endpoints were progression to ventilation if not already ventilated, and time-to-discharge from hospital. Final log-rank and Kaplan-Meier analyses are presented for remdesivir, and are appended for all four study drugs. Meta-analyses give weighted averages of the mortality findings in this and all other randomised trials of these drugs among hospital inpatients. Solidarity is registered with ISRCTN, ISRCTN83971151, and ClinicalTrials.gov, NCT04315948.

**Findings:**

Between March 22, 2020, and Jan 29, 2021, 14 304 potentially eligible patients were recruited from 454 hospitals in 35 countries in all six WHO regions. After the exclusion of 83 (0·6%) patients with a refuted COVID-19 diagnosis or encrypted consent not entered into the database, Solidarity enrolled 14 221 patients, including 8275 randomly allocated (1:1) either to remdesivir (ten daily infusions, unless discharged earlier) or to its control (allocated no study drug although remdesivir was locally available). Compliance was high in both groups. Overall, 602 (14·5%) of 4146 patients assigned to remdesivir died versus 643 (15·6%) of 4129 assigned to control (mortality rate ratio [RR] 0·91 [95% CI 0·82–1·02], p=0·12). Of those already ventilated, 151 (42·1%) of 359 assigned to remdesivir died versus 134 (38·6%) of 347 assigned to control (RR 1·13 [0·89–1·42], p=0·32). Of those not ventilated but on oxygen, 14·6% assigned to remdesivir died versus 16·3% assigned to control (RR 0·87 [0·76–0·99], p=0·03). Of 1730 not on oxygen initially, 2·9% assigned to remdesivir died versus 3·8% assigned to control (RR 0·76 [0·46–1·28], p=0·30). Combining all those not ventilated initially, 11·9% assigned to remdesivir died versus 13·5% assigned to control (RR 0·86 [0·76–0·98], p=0·02) and 14·1% versus 15·7% progressed to ventilation (RR 0·88 [0·77–1·00], p=0·04). The non-prespecified composite outcome of death or progression to ventilation occurred in 19·6% assigned to remdesivir versus 22·5% assigned to control (RR 0·84 [0·75–0·93], p=0·001). Allocation to daily remdesivir infusions (*vs* open-label control) delayed discharge by about 1 day during the 10-day treatment period. A meta-analysis of mortality in all randomised trials of remdesivir versus no remdesivir yielded similar findings.

**Interpretation:**

Remdesivir has no significant effect on patients with COVID-19 who are already being ventilated. Among other hospitalised patients, it has a small effect against death or progression to ventilation (or both).

**Funding:**

WHO.

## Introduction

In March 2020, WHO undertook Solidarity, a large, simple, international, open-label, randomised trial in patients hospitalised with COVID-19. It was designed and conducted by WHO in collaboration with national co-ordinators and principal investigators in 35 countries. Mortality was the primary endpoint, and the protocol-specified primary aim was to help to assess any effects of the study drugs on inpatient mortality, subdivided by disease severity at the time of randomisation. The two protocol-specified secondary endpoints were any effects on progression to ventilation in those not already ventilated, and on time to discharge. In early 2020, no specific treatments had been developed for COVID-19, so Solidarity started with repurposed drugs for other conditions. These could be quickly donated by manufacturers and approved by many national regulators for inclusion in a trial involving ordinary hospitals in dozens of countries. Following advice from an ad-hoc WHO working group, the initial aim was to evaluate remdesivir, hydroxychloroquine, lopinavir, and interferon (IFN)-β1a. For each of the four study groups, patients would be randomly allocated either the study drug or its control (yielding overlap between the control groups for different drugs). The trial would be open-label, so patients randomly allocated to receive no study drug would not be given placebos. This open-label design, which should yield unbiased estimates of any effects on mortality or progression to ventilation, was used to simplify the trial procedures and, hence, increase the study size to a point where any realistically moderate differences in mortality could be assessed reliably.

**Research in context**

**Evidence before this study**

Since March 2020, the open-access COVID-NMA collaboration between WHO, five Cochrane centres, and ten other groups has conducted each week systematic searches of trial registries and of study reports (in any language) to identify any randomised trial of COVID-19 treatment, assessing any methodological biases in them, and extracting their results objectively. We used this resource to determine which randomised trials had provided any relevant evidence before this study did so, and to help to update our meta-analyses of the mortality findings in Solidarity and all other trials. The largest other trial of remdesivir for COVID-19 was ACTT-1, involving 1062 inpatients, which aimed to assess effects on time to recovery. In that placebo-controlled comparison, remdesivir had little effect on median time to recovery in inpatients with a poor prognosis (those already on high-flow oxygen or ventilation at enrolment), but reduced it by 1–2 days in inpatients with a good prognosis (those on no oxygen or low-flow oxygen at enrolment). At enrolment, however, the proportion of good-prognosis inpatients was significantly greater among those randomly allocated remdesivir in ACTT-1 than among those randomly allocated placebo. Analyses unadjusted for this chance imbalance exaggerated the effects of remdesivir on time to recovery, suggesting median times to recovery (remdesivir *vs* placebo) of 10 days versus 15 days in all patients, or 11 days *vs* 18 days in patients on respiratory support or with an SpO_2_ of 94% or lower. These unadjusted analyses of ACTT-1 led US, UK, and European regulatory agencies to approve remdesivir. Slightly earlier median time to recovery in good-prognosis inpatients might not imply lower overall inpatient mortality, which is driven chiefly by dysregulated immune responses. Solidarity is the only trial big enough to assess moderate effects on mortality, and its interim results excluded any large effects. Non-randomised (so-called real-world) studies have been widely disseminated, but cannot reliably demonstrate or refute realistically moderate effects.

**Added value of this study**

Based on 1245 deaths in Solidarity (83% of the deaths in all randomised trials of remdesivir among COVID-19 inpatients), the mortality rate ratio was RR 0·91 (p=0·12) overall, with RR 1·13 (p=0·32) in ventilated patients and RR 0·86 (p=0·02) in non-ventilated patients. Benefit in non-ventilated patients is supported by an RR of 0·84 (p=0·001) among them for the composite outcome of death or progression to ventilation, but not by the results in patients who had already been ventilated. Our meta-analyses of all randomised trials showed that their findings are closely consistent with Solidarity's mortality findings. Solidarity was open-label, so it assessed not just the pharmacological effects but the net effects (pharmacological and non-pharmacological) of remdesivir usage on time to discharge from hospital. Overall, open-label allocation to remdesivir delayed discharge by about 1 day during the 10-day treatment period.

**Implications of all the available evidence**

Solidarity alone, or meta-analyses of all trials, suggest no mortality reduction in already-ventilated patients, but some mortality reduction (with a wide confidence interval) in patients who are receiving oxygen but are not ventilated. However, given that high-flow and low-flow oxygen were not recorded separately at enrolment into Solidarity, it is not known whether any protective effect in non-ventilated patients extends to those on high-flow oxygen. In Solidarity and in the meta-analyses, there was low mortality (3%) in hospitalised patients with COVID-19 who were not receiving oxygen. Daily open-label remdesivir infusions might slightly delay hospital discharge in those who would have been discharged early, as inpatients might be kept in hospital to continue their remdesivir treatment.

In this adaptive trial, unpromising drugs could be stopped early. Hydroxychloroquine, lopinavir, and IFN were eventually stopped for futility, but randomisation between remdesivir and control continued until the donated supplies were running low; recruitment then ceased everywhere. Interim results have been reported.^1^ Here, we report the final results, accompanied by meta-analyses of mortality in this trial and all other randomised trials to date of the four study drugs among COVID-19 inpatients. Only for remdesivir do the final results provide much extra evidence on mortality, so full analyses of the final results for other study drugs are provided only in the appendix (pp 22–56). Hence, the present report is chiefly of the final results for remdesivir, which involve more than twice as much evidence on mortality as the interim results did.

## Methods

### Study design and participants

In the protocol,^1^ eligible patients were aged 18 years or older, admitted to a collaborating hospital with definite COVID-19 (in the view of the responsible physician; PCR confirmation was not required), not known to have received any of the study drugs, not expected to be transferred elsewhere within 72 h, and had, in the physician's view, no contraindication to any locally available study drug. The protocol did not define contraindications to enrolment, but mentioned three possible contraindications (serious chronic liver or heart disease, or pregnancy). Written informed consent was provided by patients or, if incapable, a legal representative. Consent forms were retained by the signatories, but photos of them were to be encrypted for records. Trial procedures were minimal but rigorous, with data entry through a cloud-based data management system that complied with Good Clinical Practice and recorded demographic characteristics, respiratory support status, co-existing illnesses, and the local availability of each study drug that was still being evaluated in Solidarity before finalising trial entry (if at least one such drug was locally available) by generating in the study's central computer the treatment assignment by unstratified randomisation. Enrolment of consented patients via the trial website took just a few minutes.

Patients were subdivided by disease severity (defined by ventilation and supplemental oxygen use) at entry. The questions defining disease severity at entry did not separate high-flow from low-flow oxygen, or non-invasive from invasive ventilation.

The same cloud-based system was used to report death in hospital or discharge alive, the probable causes of any deaths, respiratory support usage, drug usage, and any suspected adverse reactions. National and global study monitors raised queries or resolved them, and checked study progress and data completeness.

The protocol for Solidarity has been published previously^1^ and was approved by the WHO ethics committee, with local protocols approved nationally. WHO and national governments were co-sponsors. Trial conduct accorded with Helsinki Declaration and Good Clinical Practice principles. Trial governance was by the steering committee and its executive group. Data and safety monitoring committee analyses were unseen by the executive group or WHO, with two exceptions. After external evidence of futility emerged for hydroxychloroquine and lopinavir, the executive group saw analyses just of those two drugs, and stopped assigning patients to them. After deciding blindly to report interim results, the executive group saw all analyses and stopped IFN for futility. Randomisation between remdesivir and its control continued, but the executive group eventually stopped randomisation into Solidarity because the donated supplies had run low.

### Randomisation and masking

We used open-label, unstratified randomisation. The study drugs were remdesivir, hydroxychloroquine, lopinavir (always given with ritonavir to slow hepatic clearance), and IFN-β1a (given with lopinavir until July 4, 2020). After receiving all data on a new patient and being told which study drugs were locally available (at least one had to be), the central computer assigned that patient, by unstratified randomisation in equal proportions, between the locally available options—ie, an available study drug or control (no study drug). No placebos were used. All patients were, in addition to any study drugs, to receive the local usual standard of care. Assignment of a patient to no study drug when more than one study drug was locally available put that patient into the control group for each of the locally available drugs. Hence, there was partial overlap among the control groups. Each comparison between patients allocated to receive a study drug and its control was evenly randomised and unbiased, so in expectation both groups would be affected equally by differences between countries, hospitals, or time periods, and by variation in patient characteristics or management.

### Procedures

Drugs were administered to patients as scheduled in the protocol (unless thought contraindicated by the responsible physician) or until patients were discharged. Briefly, patients assigned to remdesivir received, via daily intravenous infusion, 200 mg on day 0 and 100 mg on days 1–9. Patients assigned to hydroxychloroquine received, orally, 4 tablets at hour 0, 4 tablets at hour 6, and, starting at hour 12, 2 tablets every 12 h for 10 days. Each tablet contained 200 mg hydroxychloroquine sulphate (155 mg hydroxychloroquine base). The little-used option of chloride instead of sulphate also included 155 mg hydroxychloroquine base per tablet. Patients assigned to lopinavir received, orally, 2 tablets twice daily for 14 days. Each tablet contained 200 mg lopinavir (plus 50 mg ritonavir). Patients received no trial lopinavir while unable to swallow. Patients assigned to IFN-β1a mainly received, via subcutaneous injection, 44 μg on days 0, 3, and 6. A little-used alternative was daily intravenous infusion of 10 μg for 6 days. Where intravenous IFN was available, it was restricted to patients receiving high-flow oxygen, ventilation, or extracorporeal membrane oxygenation.

### Outcomes

The primary outcome used to assess the effects of study drugs was in-hospital mortality (regardless of whether before or after day 28), subdivided by disease severity at study entry. Palliative discharges were counted as in-hospital deaths, not discharges.

The two secondary outcomes were initiation of ventilation (yes or no) and duration of hospital stay (time from study entry to discharge). Although no placebos were used, appropriate analyses of these secondary outcomes can still be informative. Composite analyses of ventilation or death in those not ventilated at entry are also reported. There was, however, no formal statistical analysis plan for Solidarity, and this composite outcome was not specified in the protocol. Cause-specific mortality was not a primary or secondary outcome, although cardiac-related deaths are analysed in the appendix (p 52).

Add-on studies led from Canada,^2^ France,^3^ and Norway^4^ recorded other outcomes not reported here. The study in Canada continued randomising remdesivir versus its control for 2 additional months (including another 323 patients), but not as part of Solidarity.

### Statistical analysis

In regards to sample size, the protocol merely stated “The larger the number entered the more accurate the results will be, but numbers entered will depend on how the epidemic develops […] it may be possible to enter several thousand hospitalised patients with relatively mild disease and a few thousand with severe disease, but realistic, appropriate sample sizes could not be estimated at the start of the trial.”

**Figure 1:**
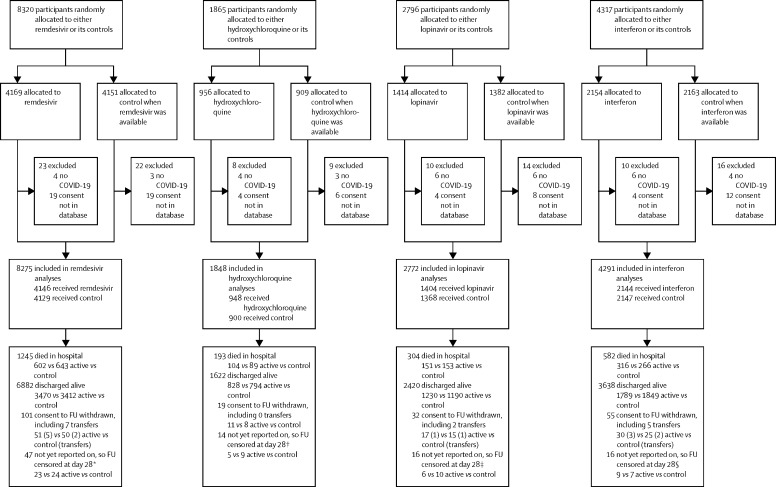
Trial profile 14 304 hospital inpatients were randomly allocated (with equal probability) between the local standard of care (control group) and whichever of the four study drugs (active group) were locally available. 83 patients with a refuted COVID-19 diagnosis (all of whom survived) or with no encrypted image of their signed consent forwarded to the database were excluded, leaving 14 221 patients included. For each study drug, the control participants for that drug were those who could have been randomly allocated to receive it but were, by chance, randomly allocated to receive the same management without it. IFN=interferon. *Entry ended on Jan 29, 2021. †Entry ended on June 19, 2020. ‡Entry ended on July 4, 2020. §Entry ended on Oct 16, 2020.

All analyses were conducted according to the randomly assigned treatment, regardless of the actual treatment, excluding patients with a refuted COVID-19 diagnosis or consent not encrypted into the database (figure 1). All entry data were recorded irrevocably before unstratified, computerised treatment assignment, yielding strict 1:1 randomisation with no foreknowledge of whether assignment would be to a particular drug or its controls.

The protocol-specified primary analyses were of in-hospital mortality split by disease severity at entry. Severity was defined by ventilation and supplemental oxygen use recorded at entry, without distinguishing between low-flow and high-flow oxygen. Mortality rate ratios (RRs) or, equivalently, hazard ratios (HRs) and their p values were calculated from log-rank or Cox analyses, stratified by three age groups (<50 years, 50–69 years, and ≥70 years) and three respiratory support groups (none, oxygen only, and ventilated), yielding 3 x 3=9 strata.

Mortality RRs describe only the proportional risk reductions, but the absolute risk reductions depend additionally on background risks. Graphs of mortality by time are from unstratified Kaplan-Meier methods, modified to assess in-hospital mortality. (Hence, the Kaplan-Meier denominators at each time include previously discharged patients. For example, if 99 of 100 patients were discharged alive before the last of them died, in-hospital mortality would be 1%, so at the time of that death the probability of not having died in hospital would be multiplied by 99/100.)

The risk on day N was calculated by first excluding patients with an outcome not reported or entry fewer than N days before dataset closure (or withdrawal of consent to follow-up or transfer elsewhere before day N). Then, the number of in-hospital deaths on day N was divided by the total number of patients in the hospital on day N or discharged alive before day N. This denominator (or risk set) was also used to calculate the contribution of day N to log-rank analyses and Cox analyses of in-hospital mortality. Denominators for the deaths on day 0, but not on later days, included patients with no follow-up reported (as deaths on day 0 would probably have been reported).

If the stratified log-rank observed minus expected number of deaths is O − E with variance V, log_e_ RR is calculated as (O − E) / V with variance 1 / V and a normal distribution.^5^ All CIs are 95%, with no allowance for multiple comparisons despite the dangers of unduly data-dependent emphasis on particular subgroups. Forest plots include χ^2^ statistics (based on [O − E]^2^ / V) to test for heterogeneity between RRs. In general, the more deaths in a stratum the larger is V and the smaller is 1/V, the variance of log_e_ RR, so V is the weight that stratum gets.

### Meta-analyses

We searched the WHO Cochrane-curated registry^6^ of COVID treatment trials—which is updated fortnightly and describes its exhaustive, detailed literature search and duplicate exclusion strategies—for randomised trials of the four study drugs. All unconfounded, randomised inpatient trials of the study drugs were extracted, excluding only any separately published parts of Solidarity.^2^, ^3^, ^4^ Published reports of the remaining trials were inspected to obtain, as reliably as possible, intention-to-treat analyses of all-cause mortality. In many major trials, mortality was monitored only until day 28 after study entry. Trials with no deaths reported were excluded. Meta-analyses involved calculation or estimation for each study (from 2 *x* 2 tables, from log-rank analyses, or from hazard ratios and their CIs) of the observed minus expected number of deaths in those allocated to receive study drug (O − E) and its variance (V). These were then summed over all studies, and used as described to get an inverse-variance-weighted average (on a log scale) of the RRs in all studies.

Meta-analyses of the major trial results are based on the inverse-variance-weighted average of b=log_e_ RR from each stratum of each trial (using odds ratios when RRs or, equivalently, HRs were unavailable). Conveniently, this approach means the weight in each stratum is V, and log_e_ RR times this weight is (O − E), so the weighted average is derived from the simple sums of (O − E) and of V over all relevant strata.^5^

For this weighted average to be medically informative, homogeneity of the averaged RRs is not necessary.^5^ So, the commonly-used name of fixed effect meta-analysis for it is inappropriate. The variances attributed to the result in each stratum, and to the overall weighted average, reflect only the play of chance at randomisation.

These methods work because log-rank and Cox methods are intimately connected. If b denotes log_e_ RR, L(b) denotes Cox log-likelihood, and event times are accurate, then the first and second derivatives of L(b) at b=0 are (O − E) and –V, which is why (O − E) / V is a useful estimator of log_e_ RR. We used SAS (version 9.4) and R (version 4.1.2) for all statistical analyses.

Solidarity is registered with ISRCTN, ISRCTN83971151, and ClinicalTrials.gov, NCT04315948.

### Role of the funding source

Study drugs were donated: remdesivir by Gilead Sciences; hydroxychloroquine by Mylan; lopinavir by AbbVie, Cipla, and Mylan; and IFN by Merck KGaA (subcutaneous) and Faron Pharmaceuticals (intravenous). The funder and drug donors had no role in study design, data collection, data analysis, data interpretation, or writing of the report.

## Results

Between March 22, 2020, and Jan 29, 2021, 14 304 patients were enrolled from 454 hospitals in 35 countries in six WHO regions. After 83 (0·6%) were excluded because their COVID-19 diagnosis was refuted or their consent was not encrypted into the study database, 14 221 patients were included in the intention-to-treat analysis. Figure 1 shows the numbers excluded and included in each treatment comparison; no exclusion biases are apparent, and the 83 exclusions are henceforth ignored. There is partial overlap between the four control groups, but this does not complicate any of the pairwise comparisons between a study drug and its controls. In the remdesivir comparison the diagnosis was reliable; only seven (<0·1%) of 8320 patients had it refuted.

The table describes the characteristics of these 14 221 patients, the relevance of these characteristics to in-hospital mortality, and their distribution between each study drug (active group) and its controls (control group). For each drug, all patient characteristics were reasonably well balanced between the study drug and control groups. The strongest determinant of risk was respiratory support at entry (none, oxygen only, or ventilation). The table also describes compliance with the random allocation among patients with in-hospital outcomes reported. Of 4077 such patients allocated remdesivir, 3892 (95·5%) were taking remdesivir halfway through the scheduled treatment period, compared with 73 (1·8%) of 4057 such patients allocated to control. There was little difference between treatment groups in use of corticosteroids (2782 [67·1%] of 4146 for remdesivir *vs* 2820 [68·3%] of 4129 for control), IL-6 inhibitors, or other non-study drugs (appendix p 23). Compliance was similarly high for the other three treatment comparisons.

**Table:** Baseline characteristics by random allocation, and compliance with that allocation

			**Included in analyses**		**Remdesivir *vs* its control**		**Hydroxychloroquine *vs* its control**		**Lopinavir *vs* its control**		Interferon *vs* its control*	
			Entered study, n (%)	Died, n (%)	Active	Control	Active	Control	Active	Control	Active	Control
Number of patients			14 221 (100%)	1989 (14·0%)	4146	4129	948	900	1404	1368	2144	2147
Age, years												
	<50		4771 (33·5%)	335 (7·0%)	1310	1326	336	316	513	504	759	731
	50–69		6443 (45·3%)	917 (14·2%)	1920	1908	410	394	602	594	970	1017
	≥70		3007 (21·1%)	737 (24·5%)	916	895	202	190	289	270	415	399
Respiratory support												
	No oxygen at entry		3627 (25·5%)	116 (3·2%)	869	861	346	338	527	535	503	508
	On oxygen at entry		9453 (66·5%)	1409 (14·9%)	2918	2921	518	480	765	718	1497	1503
	Already ventilated		1141 (8·0%)	464 (40·7%)	359	347	84	82	112	115	144	136
Bilateral lung lesions												
	No		1403 (9·9%)	91 (6·5%)	421	371	118	124	193	210	128	122
	Yes		11 468 (80·6%)	1678 (14·6%)	3326	3341	714	670	1048	1001	1857	1865
	Not imaged at entry		1350 (9·5%)	220 (16·3%)	399	417	116	106	163	157	159	160
Days in hospital before study entry												
	0		3681 (25·9%)	416 (11·3%)	888	892	296	280	422	401	707	702
	1		4819 (33·9%)	601 (12·5%)	1462	1459	319	307	450	444	700	699
	≥2		5721 (40·2%)	972 (17·0%)	1796	1778	333	313	532	523	737	746
Geographical location												
	Europe† or Canada		4342 (30·5%)	555 (12·8%)	1649	1594	287	266	348	353	268	256
	Latin America‡		2142 (15·1%)	499 (23·3%)	558	593	97	96	147	148	493	495
	Asia and Africa§		7737 (54·4%)	935 (12·1%)	1939	1942	564	538	909	867	1383	1396
Other characteristics												
	Sex											
		Male	8851 (62·2%)	1343 (15·2%)	2601	2639	574	532	852	800	1342	1331
		Female	5370 (37·8%)	646 (12·0%)	1545	1490	374	368	552	568	802	816
	Current smoker		975 (6·9%)	129 (13·2%)	247	233	93	82	137	121	142	147
	History of											
		Diabetes	3685 (25·9%)	630 (17·1%)	1129	1120	201	205	346	326	517	563
		Heart disease	3110 (21·9%)	576 (18·5%)	929	935	194	194	292	291	457	487
		Chronic lung disease	910 (6·4%)	194 (21·3%)	284	281	63	65	94	86	120	110
		Asthma	755 (5·3%)	91 (12·1%)	247	242	44	47	68	57	79	105
		Chronic liver disease	191 (1·3%)	41 (21·5%)	57	72	15	14	16	23	14	24
Number of patients who were taking the study drug midway through its scheduled duration, n/N (%)¶			..	..	3892/4077 (95·5%)	73/4057 (1·8%)	876/932 (94·0%)	45/883 (5·1%)	1299/1381 (94·1%)	27/1344 (2·0%)	1987/2108 (94·3%)	31/2117 (1·5%)
Of those eventually discharged, proportion of patients who were still in hospital on												
	Day 7		..	..	68·8%	62·5%	64·9%	54·5%	68·6%	60·0%	58·5%	53·7%
	Day 14		..	..	25·9%	24·7%	24·0%	20·2%	32·3%	22·9%	21·2%	20·7%
	Day 21		..	..	12·4%	12·5%	11·8%	10·6%	12·2%	12·1%	9·5%	8·9%

The pairwise comparisons are of each drug *vs* its own controls. Patients in different pairwise comparisons might overlap, so the total number in any comparison is only 14 221 (and not 17 186). Here and elsewhere, the few (always <0·1%) with a particular characteristic not known are merged with the largest category of that characteristic: nine merged with male, three merged with age 50–69 years, five merged with previous days in hospital before study entry ≥2. The number who died is the number reported as dying in hospital (before or after day 28) before ever having been discharged.

* Interferon randomisation was to interferon plus lopinavir *vs* lopinavir until July 4, 2020, then to interferon *vs* no study drug.

† Albania, Austria, Belgium, Finland, France, Georgia, Ireland, Italy, Lithuania, Luxembourg, North Macedonia, Norway, Portugal, Spain, and Switzerland.

‡ Argentina, Brazil, Colombia, Honduras, and Peru.

§ Egypt, Ethiopia, India, Indonesia, Iran, Kuwait, Lebanon, Malaysia, Mali, Oman, Pakistan, Philippines, Saudi Arabia, and South Africa.

¶ Compliance is calculated only among those with an in-hospital outcome reported, and is defined as the proportion of patients taking the study drug midway through its scheduled duration (or midway through the time from entry to death or discharge, if this is shorter).

Detailed analyses comparing each study drug with its control are shown in the appendix (pp 22–56). For the three study drugs that were discontinued for futility at or before publication of the interim analyses, the final results add relatively little information to the published interim results, and for each there is still no good evidence of harm or of benefit. The adverse effects of IFN-β1a on in-hospital mortality suggested by figure S1 (appendix p 25) are not supported by the multivariate analyses (appendix p 24), or by the analyses of progression to ventilation (appendix pp 35–44). For remdesivir, however, there is a substantially greater amount of data to report than in the interim analyses (as random allocation to remdesivir was continued), so only for remdesivir are the final analyses fully presented and discussed.

**Figure 2:**
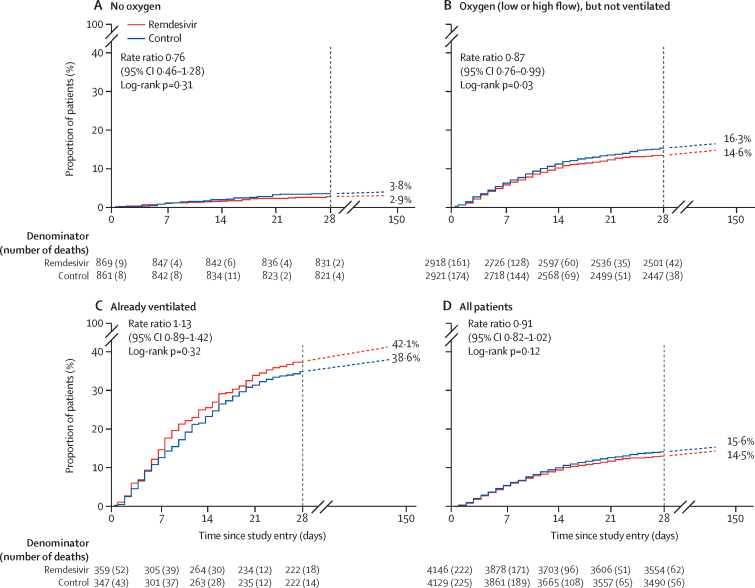
Primary outcome of in-hospital mortality for remdesivir *vs* its control, by respiratory support at study entry Kaplan-Meier graphs to day 28, then total in-hospital mortality after day 28 (dashed lines); all known deaths were before day 150. Kaplan-Meier denominators include all patients except those who had already died in hospital and the few already lost to follow-up. The log-rank mortality rate ratio is standardised for age and respiratory support, and uses all in-hospital deaths, before or after day 28.

The primary outcome was in-hospital mortality, overall and subdivided by disease severity at entry. This outcome is plotted against time from study entry in figure 2 and in the appendix (pp 25–29). With more than twice as many deaths as before, the overall mortality findings for remdesivir (in all patients) exclude substantial benefit or harm, but do not exclude either moderate effects or zero effects on mortality. Of 8275 patients in the overall remdesivir analyses, 602 (14·5%) of 4146 assigned to remdesivir and 643 (15·6%) of 4129 assigned to controls died (RR 0·91 [95% CI 0·82–1·02], p=0·12). These analyses of in-hospital mortality include 15 palliative discharges in the remdesivir group and 11 in the control group.

Subdivision by disease severity suggested a less favourable RR in more severe disease (trend test χ^2^_1_=3·9, p=0·05; appendix p 31). Among the 1730 patients not on oxygen initially, 25 (2·9%) of 869 assigned to remdesivir died, as did 33 (3·8%) of 861 assigned to control (RR 0·76 [0·46–1·28], p=0·30). Among the 5839 non-ventilated patients on low-flow or high-flow oxygen initially, 426 (14·6%) of 2918 assigned to remdesivir died versus 476 (16·3%) of 2921 assigned to control (RR 0·87 [0·76–0·99], p=0·03). Of 706 patients already ventilated, mortality was 42·1% for those assigned to remdesivir (151 of 359) versus 38·6% for those assigned to control (134 of 347; RR 1·13 [0·89–1·42], p=0·32).

**Figure 3:**
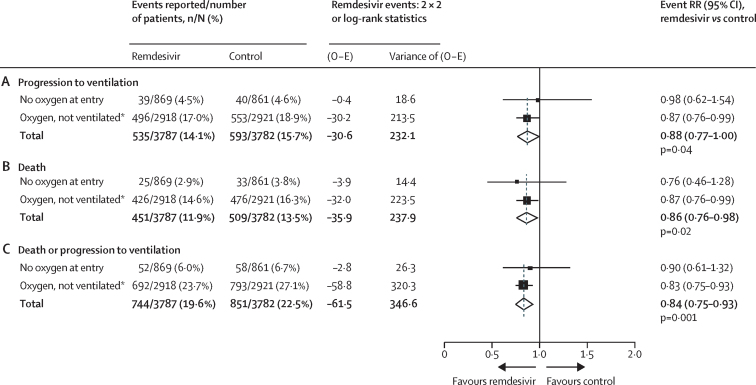
Secondary outcome of ventilation initiation for remdesivir *vs* its control in patients not already ventilated at study entry 2 X 2 analyses of ventilation, log-rank analyses of death, and combined analyses of death or ventilation. Analyses are stratified by age and by respiratory support, so each total is stratified for both factors. O − E=observed minus expected number of events. RR=rate ratio. *High-flow and low-flow oxygen were not recorded separately at entry into Solidarity.

Among those not already ventilated, 14·1% of patients assigned to remdesivir versus 15·7% patients assigned to control progressed to ventilation (RR 0·88 [0·77–1·00], p=0·04), 11·9% versus 13·5% died (RR 0·86 [0·76–0·98], p=0·02), and 19·6% versus 22·5% died or progressed to ventilation (RR 0·84 [0·75–0·93], p=0·001; figure 3).

Of 1730 patients not on supplemental oxygen initially, 4·5% of patients assigned to remdesivir (*vs* 4·6% assigned to control) progressed to ventilation and 6·0% versus 6·7%, respectively, died or had ventilation initiated (RR 0·90 [0·61–1·32], p=0·59). Of 5839 on low-flow or high-flow oxygen initially, 17·0% of patients assigned to remdesivir (*vs* 18·9% assigned to control) progressed to ventilation and 23·7% versus 27·1%, respectively, died or had ventilation initiated (RR 0·83 [0·75–0·93], p=0·001). Further details are given in the appendix (p 22). Multiple subgroup analyses of death or ventilation (or both) are also in the appendix (pp 30–44).

Figure 3 shows the net effect of allocation to open-label remdesivir was to delay discharge by about 1 day during the 10-day treatment period; later discharges were unaffected. Further details are in the appendix (pp 45–50).

Directly randomised comparisons between one study drug and another are also included in the appendix (p 51), none of which suggested that remdesivir reduced time-to-discharge. Before IFN was discontinued for futility, 1997 patients had been concurrently randomised between it and remdesivir in hospitals where both drugs were, at that time, locally available. In this directly randomised, concurrent comparison of two parenteral treatment regimens there was no trend towards earlier discharge among those allocated remdesivir than among those allocated to IFN. No study drug significantly affected cardiac mortality (appendix p 52).

Meta-analyses, updated since those in the interim Solidarity report,^1^ of overall mortality in the randomised trials of remdesivir, and of the other three study drugs among hospitalised patients, are included in the appendix (pp 53–56). The results of all four meta-analyses are numerically reliable, given that each is dominated by its two largest trials, both of which have reported the numbers of participants randomly allocated to treatment and mortality rates by allocated treatment.

## Discussion

The additional evidence on remdesivir versus its control has not materially altered the mortality RRs in the interim Solidarity results,^1^ overall or in subgroups defined by the type of respiratory support being given at the time of randomisation (no oxygen, oxygen, or ventilation). This additional evidence has, however, reduced the statistical uncertainty in the mortality RRs. The final results of Solidarity have also provided better evidence on the secondary endpoints of progression to ventilation, and time to discharge from hospital. Time to discharge is discussed first, as quantitative estimates of the effects of remdesivir on time to fitness for discharge—based on unadjusted analyses of data from the ACTT-1 placebo-controlled trial with 1062 patients^7^—underlay the regulatory approval of remdesivir.^8^, ^9^, ^10^ Similar unadjusted analyses of that trial were also used by the manufacturer of remdesivir to help to determine the price initially charged for remdesivir, based on the health economic benefits if hospital stay could be reduced by several days.^11^, ^12^

**Figure 4:**
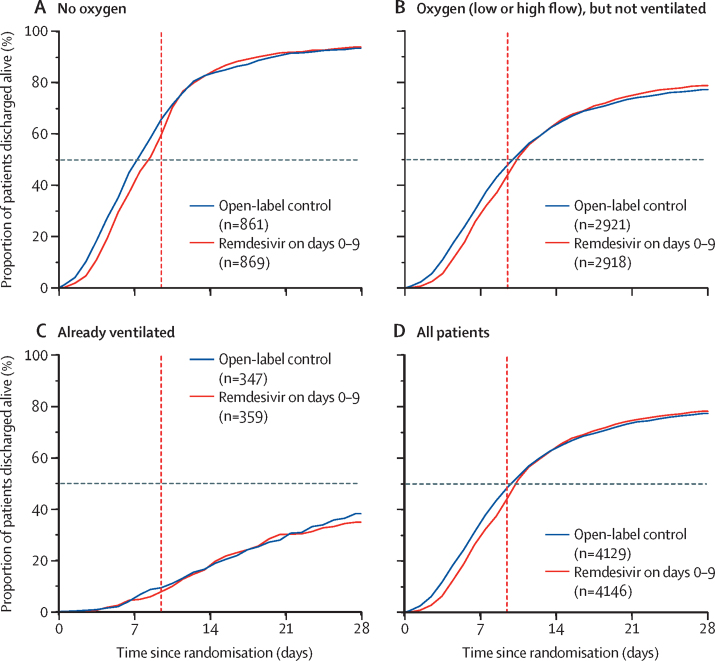
Secondary outcome of time-to-discharge alive from hospital, subdivided by respiratory support at study entry All enrolled patients with outcomes reported are included. Vertical red line shows the end of scheduled treatment duration (10 days) if still in hospital. Horizontal lines at 50% crossed graphs at median time to discharge.

Solidarity randomly allocated 8275 patients to remdesivir or open control, and has reliably shown that allocation of patients to open-label remdesivir infusions did not reduce time-to-discharge. As Solidarity is an open-label trial, the net effects of remdesivir on time to discharge from hospital combine any pharmacological effects of remdesivir on time to fitness for discharge with any non-pharmacological effects (eg, of knowledge as to whether the patient was receiving active treatment) on time chosen to discharge the patient. Figure 4 reports the sum of these two effects, showing that, because knowledge that patients are being given a potentially active treatment can delay discharge from hospital, the net effect of allocation to open-label remdesivir was to increase time to discharge by about 1 day during the 10-day scheduled treatment period; later discharges were unaffected. Delaying discharge by about 1 day during a modern 5-day scheduled treatment period but not after it would, however, affect only those who would otherwise be discharged within the first 5 days.

Delays in the discharge of patients who would be discharged within the first 5 or 10 days does not mean remdesivir has no pharmacological effect on time to fitness for discharge, but it does mean that any such effect is not large and that, in Solidarity, it was outweighed by the non-pharmacological effect of patients remaining in hospital to continue remdesivir infusions. Moreover, before IFN-β1a was discontinued for futility, almost 2000 patients had been randomly allocated between it and remdesivir, with no difference between these two parenteral regimens in time to discharge while both were scheduled to continue. This finding provides further evidence that any pharmacological effect of remdesivir on time to discharge is not large.

**Figure 5:**
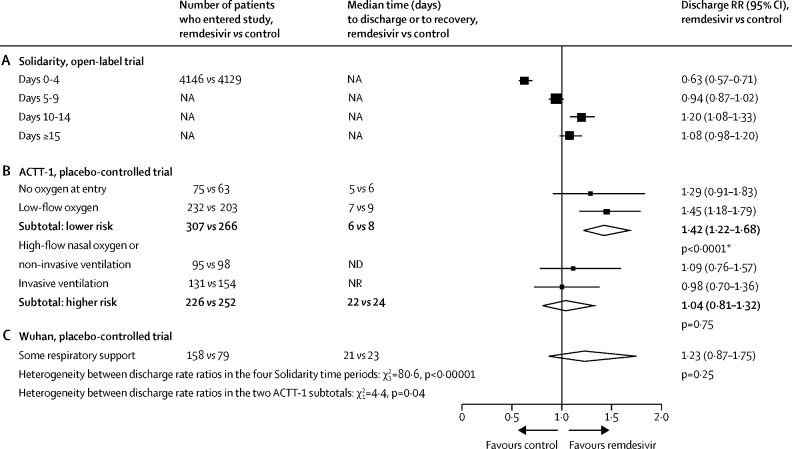
Comparison between the effects of random allocation to remdesivir on the daily discharge rate in Solidarity and in two placebo-controlled trials (A) Solidarity data are shown as treatment effects during different time periods. (B) ACTT-1 data are shown as treatment effects, split by initial respiratory support. (C) Wuhan data are shown as treatment effects among all patients. NA=not applicable. ND=not done. NR=median not reached. RR=rate ratio. *In ACTT-1, there was a chance imbalance favouring remdesivir in the initial proportions at higher risk, as defined in this figure (remdesivir: 226 [42%] of 533 *vs* placebo: 252 [49%] of 518, p=0·02). So, any findings combining low-risk and high-risk patients in that trial depend on whether this imbalance is allowed for. Cited ACTT-1 analyses are from the published text and its appendix.^7^

Because it is an open-label trial, Solidarity yields estimates of the net effects of remdesivir on time to discharge (pharmacological and non-pharmacological), whereas placebo-controlled trials yield estimates of only the pharmacological effects. We compared the findings in Solidarity with those in two placebo-controlled trials (figure 5).^7^, ^13^ For Solidarity, the figure gives discharge RRs from Cox analyses of time to discharge, subdivided by days since entry. These show that the net effect of allocation to remdesivir was to delay discharges during the scheduled treatment period, with catch-up just after the period ended. For ACTT-1,^7^ it gives discharge RRs from Cox analyses of time to fitness for discharge (ie, recovery), subdivided by respiratory support at entry. These RRs show that the pharmacological effect of remdesivir was to hasten recovery in lower-risk patients (ie, those on no oxygen or low-flow oxygen), with little effect on time to recovery in higher-risk patients.

Medians are given for the placebo-controlled trials, but these are reliable descriptive statistics only if the recovery probabilities increase rapidly across the 50% mark, as in the lower-risk patients in ACTT-1. In those patients, remdesivir reduced median time to fitness for discharge by 1–2 days. By chance, as shown in figure 5, significantly more of those lower-risk patients had been randomly allocated to remdesivir than to control. Analyses^7^, ^8^, ^9^, ^10^ unadjusted for this chance imbalance misleadingly suggested median times to recovery (remdesivir *vs* placebo) of 10 days versus 15 days in all patients, or 11 days versus 18 days in patients with an SpO_2_ of less than 94% or respiratory support at study entry. However, within each of the four categories of respiratory support in figure 5 there were no such big differences.

**Figure 6:**
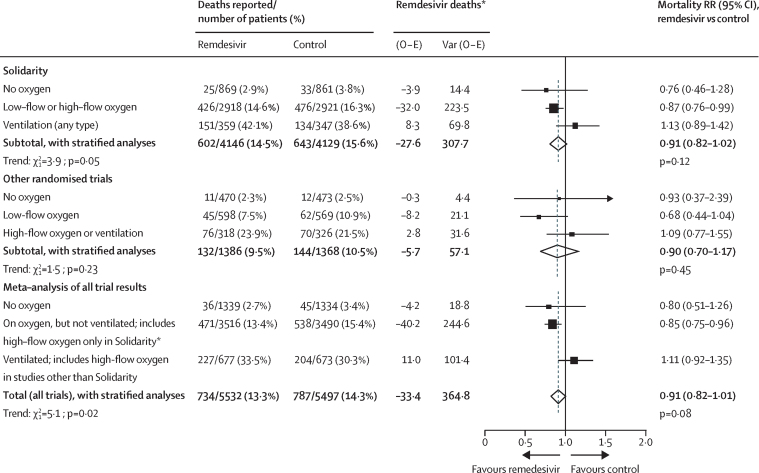
Meta-analysis of the effects of remdesivir *vs* control on mortality in Solidarity and other trials, by respiratory support at study entry High-flow and low-flow oxygen were not recorded separately at entry into Solidarity. Ventilation includes non-invasive ventilation. Full details of these meta-analyses are given in the appendix (p 53). Solidarity data are from figure 2 and table 2, and other data are published (supplementary table 10).^7^ O − E=observed minus expected number of deaths. RR=rate ratio. *If V is the variance of the log-rank statistic O − E then RR is obtained by taking log_e_ RR to be (O − E) / V with normal variance 1 / V. Summation of (O − E) and of V yields the stratified total (providing the inverse-variance-weighted average of the separate log_e_ RR values).

An anti-viral treatment that reduces time to recovery in lower-risk inpatients might have little effect on overall inpatient mortality, because this outcome is driven chiefly by dysregulated immune responses. The interim findings from Solidarity excluded any large effects on mortality. The final findings include 83% of the deaths in all randomised inpatient trials of remdesivir (and this 83% includes part^2^ or all^3^, ^4^ of the three particular parts of Solidarity that have already been published separately). Hence, a weighted average of the stratified mortality findings in Solidarity and other trials yields a meta-analysis (stratified by respiratory support at entry) with results that differ little from those from Solidarity alone (figure 6). The overall mortality RRs were 0·91 (0·82–1·02, p=0·12) in Solidarity alone and 0·91 (0·82–1·01, p=0·08) in the meta-analysis, and the apparent dependence of RR on respiratory support at entry was similar in Solidarity and in the meta-analysis.

Categorisation of respiratory support at entry differed between trials. The data recorded at entry into Solidarity did not separate low-flow and high-flow oxygen, although prognosis might be about as poor with high-flow oxygen as with non-invasive ventilation. Conversely, some other trial reports did not distinguish between high-flow oxygen and non-invasive ventilation. This disparity complicates the combination of evidence from Solidarity with that from the other trials; therefore, figure 6 gives the results for Solidarity alone and for all other trials separately as well as together. Despite the absence of exact comparability, three highly prognostic strata could be defined: no oxygen; not ventilated but on oxygen (including high-flow oxygen in Solidarity, but not other trials); and ventilated (or, in other trials, on high-flow oxygen). These spanned a 10-fold range of mortality in the control groups, from 3% with no oxygen to 30% with ventilation; most patients were in the middle stratum, with 15% mortality.

In the meta-analysis (as in Solidarity) the mortality RR (remdesivir *vs* control) was somewhat adverse in the ventilated subgroup and somewhat favourable in the other two subgroups (p=0·006 for both non-ventilated subgroups combined, with p=0·02 for the trend in the RR across all three subgroups), but it is not known whether any benefit in non-ventilated patients extends to those on high-flow oxygen.

As the mortality reduction in all subgroups combined is not definite and subgroup analyses can easily be misleading,^14^ the apparent mortality reduction in patients not already being ventilated is difficult to interpret without further evidence. Four results support there being at least some effect on mortality in non-ventilated patients. First, in Solidarity, one of the two protocol-specified secondary outcomes was progression to ventilation in non-ventilated patients. This outcome was reduced by allocation to remdesivir (p=0·04), as was the composite outcome of death or progression to ventilation (p=0·001) in these patients. Second, in ACTT-1, remdesivir significantly reduced time to fitness for discharge among patients who were receiving no, or only low-flow, oxygen at entry. Third, a small placebo-controlled trial of remdesivir in non-hospitalised patients reported an 87% reduction in hospitalisation (0·7% [2/279] *vs* 5·3% [15/283], p=0·008, with no deaths by day 28).^15^, ^16^ The possibility of pre-hospital antiviral treatment causing a substantial proportional reduction—but a small absolute reduction—in hospitalisation is reinforced by placebo-controlled trials of other antivirals, two oral^17^, ^18^ and three antibody regimens.^19^, ^20^, ^21^ Two^19^, ^20^ of these antibody regimens can no longer control current variants.^22^ Fourth, an antibody regimen that substantially reduced hospital admission rates^19^ moderately reduced mortality among seronegative inpatients.^23^

Solidarity has several limitations. First, only simple information on respiratory support was collected at entry, and the reasons for needing oxygen were not recorded. Second, ventilation was more resource-limited in some countries or hospitals than others, and some patients who were not ventilated would have been ventilated had resources been available. This situation does not, however, invalidate the secondary analyses of ventilation or the composite outcome of death or ventilation (which is unaffected by any deaths that could have been prevented by ventilation). Moreover, heterogeneity between the collaborating countries and hospitals does not bias the comparison of study drug versus control, as all could give the allocated treatment and report the study outcomes reliably. Third, Solidarity recruitment preceded the delta and omicron variants (and widespread vaccination). For drugs such as remdesivir that act via internal non-structural proteins (NSPs), the emergence of these new viral variants might not materially affect drug efficacy. However, absolute effects on mortality might be smaller for lower-risk variants, or for patients whose risk during their current episode of hospitalisation for COVID-19 is reduced either by having previously been vaccinated, or by effective treatment during this episode with some other anti-viral drug(s), some effective immune-modulating drug(s), or good supportive care. Fourth, to maximise study size, controls did not receive placebo infusions, so the findings combine the pharmacological and non-pharmacological effects of allocation to daily remdesivir.

Lastly, the chief limitation of Solidarity is study size. Worldwide, over 10 000 inpatients have been randomly assigned to receive either remdesivir or control, including some 8000 in Solidarity. Although substantial effects on mortality can now be excluded, it is difficult to demonstrate or refute moderate effects, especially if these are only in particular subgroups. If it had been possible to randomise another 10 000 patients, there would now be better evidence on how to treat the next 10 million.

Non-randomised, so-called real-world, studies can involve larger numbers, but might be affected by biases as great as any realistically moderate effects on mortality among inpatients.^24^, ^25^ Three non-randomised studies^26^, ^27^, ^28^ involving 100 000 participants were cited by the manufacturer as showing remdesivir reduces inpatient mortality by 40%, regardless of respiratory support level.^29^ Of these, one claimed remdesivir halves mortality even in ventilated patients, which is reliably contradicted by the randomised evidence, thus illustrating how biased real-world evidence can be.^26^ Another, perhaps due to an opposite bias, found mortality per patient was actually higher with than without remdesivir. However, as patients given remdesivir stayed in hospital about one-third longer, that study reported (misleadingly, but correctly) that mortality per person-year was lower with remdesivir than without.^27^ The third found overall results similar to those suggested by the randomised trials,^28^ but even if non-randomised studies happen to get approximately the right answer, there is no way of knowing they have done so. Such studies can, however, be useful when extreme effects (such as those of some vaccines^30^) are to be assessed and moderate biases would be relatively unimportant.

Both in Solidarity alone and in the meta-analysis of all trials, the mortality findings among inpatients who were not already being ventilated at study entry indicate that daily remdesivir infusions can somewhat reduce the risk of death, but there is wide uncertainty. This uncertainty is due partly to random error, as indicated by the confidence intervals, and partly to uncertainty as to whether to focus on the findings in all patients or the findings in the subgroup of those not already ventilated.^14^ If, as an example, there is a reduction of about one-seventh in mortality among patients who are not already being ventilated, the number of individuals who would need to be treated to avoid one death (NNT) would depend strongly on the risk without treatment. An absolute reduction of 2% in mortality, from 14% down to 12%, would imply NNT=50, but an absolute reduction of 0·5% in mortality, from 3·5% down to 3%, would imply NNT=200. The proportional reduction in mortality is, however, too uncertain for these particular numbers to be of much relevance. Additionally, it is not known whether any protective effect in non-ventilated patients extends to those on high-flow oxygen.

Regardless of how these findings are interpreted, better drugs to treat COVID-19 will continue to be needed. Oral antiviral agents, various immune modulators, and monoclonal antibodies against currently circulating variants of concern are now emerging that might prove more effective, more convenient, or less expensive than daily remdesivir infusions, but large-scale randomised evidence will be needed to evaluate and compare them.

For more on the **COVID-NMA Initiative** see https://covid-nma.com/

Correspondence to: Dr Ana-Maria Henao-Restrepo, World Health Organisation, Geneva 1211, Switzerland **henaorestrepoa@who.int**

## Data sharing

After the trial has ended and its results have been reported, anonymised data sharing will occur as per WHO's policy statement on data sharing.
