
JOURNAL INFORMATION
==============================
NLM Title Abbreviation: Lancet
Journal ID: Lancet
NLM Journal ID: 2985213R

Lancet (London, England)

ISSN: 0140-6736
EISSN: 1474-547X

ARTICLE INFORMATION
==============================
PMCID: PMC10805002
PMID: 38185126
DOI: 10.1016/S0140-6736(24)00005-9
Article ID: S0140-6736(24)00005-9
Article version: 1
Subjects: Department of Error

Department of Error

Print publication date: 2024 Jan 13
Volume: 403
Issue: 10422
First page: 146
Last page: 146
Copyright: .
Copyright year: 2024
Copyright holder: Elsevier Ltd
License: This is an open access article under the CC BY license (http://creativecommons.org/licenses/by/4.0/).
License URL: https://creativecommons.org/licenses/by/4.0/

Erratum for: Remdesivir and three other drugs for hospitalised patients with COVID-19: final results of the WHO Solidarity randomised trial and updated meta-analyses 399 10339 21 5 2022 1941 1953 Lancet (London, England) 10.1016/S0140-6736(22)00519-0 PMC9060606 35512728

==============================
WHO Solidarity Trial Consortium. Remdesivir and three other drugs for hospitalised patients with COVID-19: final results of the WHO Solidarity randomised trial and updated meta-analyses. Lancet 2022; 399: 1941–53. The collaborators of this Article have now been indexed on PubMed. These corrections have been made online as of Jan 4, 2024.

