# Supplementary material for: Remdesivir and three other drugs for hospitalised patients with COVID-19: final results of the WHO Solidarity randomised trial and updated meta-analyses
Source: Lancet. 2022 May 21;399(10339):1941–53. doi: 10.1016/S0140-6736(22)00519-0 (PMC9060606; doi:10.1016/S0140-6736(22)00519-0)
Supplement: Supplementary appendix [file mmc1.pdf]

THE LANCET

Supplementary appendix

This appendix formed part of the original submission and has been peer reviewed. We post it as supplied by the authors.

Supplement to: WHO Solidarity Trial Consortium. Remdesivir and three other drugs for hospitalised patients with COVID-19: final results of the WHO Solidarity randomised trial and updated meta-analyses. *Lancet* 2022; published online May 2. https://doi.org/10.1016/S0140-6736(22)00519-0.

Contents list for supplementary online material

Click on a page to jump to it

3 Writing committee, data and safety monitoring committee, global monitoring and data management support team, statistical analysts, and WHO trial co-ordination team

4 International steering committee, and its executive group

6 National investigators and researchers

16 Other collaborators in participating countries

20 Acknowledgements

21 Funding

22 Table S1 Treatment allocation vs initiation of ventilation after entry and/or death

23 Table S2 Use of non-study drugs

24 Table S3 Multivariate analysis simultaneously estimating all 4 trial treatment effects

RR calculations include in-hospital deaths after day 28, but percentages cited in forest plots (except for meta-analyses) are from K-M analyses that exclude deaths after day 28.

25 Fig S1 Kaplan-Meier graphs of effects of trial treatment on in-hospital mortality

26 Fig S2 Kaplan-Meier graphs of effects on in-hospital mortality, by ventilation at entry

30 Fig S3 Mortality RRs, by age and by respiratory support at entry

31 Fig S4 Mortality RRs, by multiple subgroups of entry characteristics and by steroid use

35 Fig S5 RRs for ventilation initiation, by age and by respiratory support at entry

36 Fig S6 RRs for ventilation initiation, by multiple subgroups and by steroid use

40 Fig S7 RRs for death or ventilation initiation, by age and by respiratory support at entry

41 Fig S8 RRs for death or ventilation initiation, by multiple subgroups and by steroid use

45 Fig S9 Not ventilated: Effect on time to discharge alive (each drug vs its own control)

46 Fig S10 Ventilated: Effect on time to discharge alive (each drug vs its own control)

47 Fig S11 High/low-flow O2: Effect on time to discharge alive (each drug vs its own control)

48 Fig S12 No O2 support: Effect on time to discharge alive (each drug vs its own control)

49 Fig S13 Ventilated or O2: Effect on time to discharge alive (each drug vs its own control)

50 Fig S14 All patients: Effect on time to discharge alive (each drug vs its own control)

51 Fig S15 All patients: Effect on time to discharge alive (one study drug vs another)

52 Fig S16 Cardiac mortality in Solidarity, each drug vs its own control

53 Fig S17 Meta-analyses of mortality in Solidarity and all other trials of its 4 study drugs

Composition of the WHO Solidarity trial consortium

Writing committee
Prof Hongchao Pan PhD, Prof Richard Peto FRS, Prof Quarraisha Abdool Karim PhD, Prof Marissa M Alejandria MD, Ana-Maria Henao-Restrepo MD, César Hernández-García PhD, Marie-Paule Kieny PhD, Prof Reza Malekzadeh MD, Prof Srinivas Murthy MD, Marie-Pierre Preziosi PhD, Prof K Srinath Reddy DM, Mirta Roses-Periago MD, Vasee Sathiyamoorthy PhD, Prof John-Arne Røttingen PhD, Soumya Swaminathan MD, Michael Ryan MD.

Nuffield Department of Population Health and Medical Research Council Population Health Research Unit, Oxford University, Oxford, United Kingdom (HP, RP), Centre for the AIDS Programme of Research In South Africa (CAPRISA), Durban, South Africa (QAK), National Institutes of Health, University of the Philippines, Manila, Philippines (MMA), Agency of Medicine and Medical Devices, Madrid, Spain (CHG), Institut National de la Santé Et de la Recherche Médicale (INSERM), Paris, France (MPK), Digestive Disease Research Institute, Teheran University of Medical Sciences, Tehran, Iran (RM), University of British Columbia, Vancouver, Canada (SM), Public Health Foundation of India, New Delhi, India (KSR), National Academy of Sciences, Buenos Aires, Argentina (MRP), Norwegian Ministry of Foreign Affairs, Oslo, Norway (JAR), World Health Organization, Geneva, Switzerland (AMHR, MPP, VS, SS, MR).

Data and safety monitoring committee (DSMC)
Prof Aldo Maggioni (chair) PhD, Prof Abdel Babiker PhD, Prof Deborah Cook MD, Prof Arjen Dondorp PhD, Prof Gagandeep Kang PhD.

Associazione Nazionale Medici Cardiologi Ospedalieri Research Center, Florence, Italy (AM), University College London, London, United Kingdom (AB), McMaster University, Hamilton, Ontario, Canada (DC), Mahidol-Oxford Tropical Medicine Research Unit, Faculty of Tropical Medicine, Mahidol University, Bangkok (AD), Christian Medical College, Vellore, India (GK).

Global monitoring and data management support teams
University of Bern, Switzerland: Sven Trelle MD, Sybil McGinty PhD, Mattia Branca PhD, Sheila Appadoo MPH, Mamatha Sauermann PhD.
University of Bristol, United Kingdom: Jonathan AC Sterne PhD, Chris A Rogers PhD, Heike Cappel-Porter MMath, David Hutton, Samir Bellani BSc, Emma Allum MMath, Jana Kirwan MA.

Statistical analysts
University of Oxford, United Kingdom: Hongchao Pan, Richard Peto.

WHO trial coordination team
Virginia Benassi LLM, Ana-Maria Henao-Restrepo MD, Patrick Lydon MPhil, Maria Consuelo Miranda-Montoya MD, Marie-Pierre Preziosi PhD, Kolawole Salami MD, Vasee Sathiyamoorthy PhD, Soumya Swaminathan MD, Michael Ryan MD.

International Steering Committee

Albania: University Hospital Centre Mother Theresa, Tirana Nadia Como PhD*; National Agency for Medicines and Medical Devices, Tirana Narvina Sinani PharmD†.

Argentina: Fundación del Centro de Estudios Infectológicos (FUNCEI), Buenos Aires Gustavo Lopardo MD*; National Academy of Sciences of Buenos Aires, Buenos Aires Mirta Roses Periago MD†.

Brazil: Oswaldo Cruz Foundation Estevao P Nunes PhD*, Paula PS Reges MD†.

Canada: University of British Columbia Srinivas Murthy MD*; Public Health Agency of Canada Marina Salvadori MD†.

Colombia: Universidad Nacional de Colombia and Clinica Colsanitas Carlos A Alvarez- Moreno PhD*; Ministry of Health Maria Lucia MesaRubio MD†.

Egypt: National Hepatology and Tropical Medicine Research Institute Mohamed Hassany MD*; Ministry of Health and Population Hala Zaid PhD†.

Ethiopia: Armauer Hansen Research Institute, Addis Ababa, Ethiopia Mekonnen Teferi MD*, Abebe Genetu Bayih PhD†.

Finland: Helsinki University Hospital, Helsinki and South Karelian Central Hospital, Lappeenranta Kari AO Tikkinen PhD*; Finnish Institute for Health and Welfare and University of Finland, Helsinki Markus Perola PhD†.

France: Hospices Civils de Lyon, Lyon Florence Ader PhD*; Institut National de la Sante Et de la Recherche Medicale, Paris Marie-Paule Kieny PhD‡.

Georgia: Infectious Diseases, AIDS and Clinical Immunology Research Center Tengiz Tsertsvadze PhD*, Akaki Abutidze PhD*, Ministry of IDPs from the Occupied Territories, Labour, Health and Social Affairs Tamar Gabunia PhD†.

Honduras: National Autonomous University of Honduras Marco T Medina MD*; Secretaria de Salud de Honduras Nery Cerrato MD†.

India: Indian Council of Medical research (ICMR), New Delhi, Balram Bhargava; ICMR, National AIDS Research Institute, Pune Sheela Godbole MD*; Public Health Foundation of India K Srinath Reddy DM‡.

Indonesia: National Institute of Health Research and Development Irmansyah Irmansyah MD*; RSUP Persahabatan, Jakarta Menaldi Rasmin MD†.

Iran (Islamic Republic of): Digestive Disease Research Institute, Teheran University of Medical Sciences, Tehran Reza Malekzadeh MD*†‡.

Ireland: HRB Clinical Research Facility, University College Cork Joe Eustace MHS*; Department of Health Peter Lennon BBS†.

Italy: University of Verona Evelina Tacconelli PhD*; Italian Medicines Agency (AIFA) Nicola Magrini MD†.

Kuwait: Infectious Diseases Hospital Almonther Alhasawi MD*; Ministry of Health Abdullah Al-Bader PhD†.

Lebanon: Rafic Hariri University Hospital Pierre Abi Hanna MD*; Ministry of Public Health Rasha Hamra PharmD†.

Lithuania: Vilnius University, Institute of Clinical Medicine; Vilnius University Hospital Santaros Klinikos Ligita Jancoriene PhD*, Laimonas Griskevicius PhD†.

Malaysia: Penang HospitalTing Soo Chow MD*; Hospital Sungai Buloh, Jalan Hospital Sureh Kumar MB, BS†.

Mali: Center for Vaccine Development - CVD Mali Samba Sow MSc*†, Fadima Cheick Haidara MD*.

North Macedonia: University Clinic of Infectious Diseases and Febrile Conditions Milena Stevanovikj MSc*; Ministry of Health Suzana Manevska MD†.

Norway: Oslo University Hospital Pål Aukrust PhD*, Andreas Barratt-Due PhDt; Norwegian Ministry of Foreign Affairs John-Arne Røttingen PhD‡.

Oman: Royal Hospital, Mininstry of Health Faryal Kamis MD*, Ministry of Health Adhara Al Mawali PhD†.

Pakistan: Shaukat Khanum Memorial Cancer Hospital and Research Centre Aun Raza FCPS*, Mariam Hassan MBBS†.

Peru: Universidad Peruana Cayetano Heredia Patricia J García PhD*, Eduardo Gotuzzo MD†.

Philippines: National Institutes of Health, University of the Philippines, Manila Marissa M Alejandria MD*†‡.

Portugal: Hospital de Curry Cabral - CHULC - Infectious Disease Department Fernando Manuel Tavares Maltez PhD*; Agency for Clinical Research and Biomedical Innovation (AICIB) Helena Beaumont MSC†.

Saudi Arabia: Ministry for Preventive Health Athari O Alotaibi MSc*,Abdullah Asiri MB†.

South Africa: Centre for the AIDS Programme of Research In South Africa Quarraisha Abdool Karim PhD‡;University of the Witwatersrand Jeremy Nel MBChB *; Wits Reproductive Health and HIV Institute Helen Rees MRCGPt.

Spain: Hospital Clínico San Carlos, UCM, SCREN, IdISSC, Madrid Antonio Portoles PhD*; Agency of Medicine and Medical Devices César Hernández-Garcia PhD†‡.

Switzerland: Lausanne UniversityHospital Oriol Manuel MD*†.

*National Principal Investigator; †National Coordinator; ‡Executive Group;
§ Representing Discovery add-on study in France, Belgium, Austria and Luxembourg.

Executive Group of the International Steering Committee

John-Arne Røttingen (chair), Quarraisha Abdool Karim, Marissa Alejandria, César Hernández García, Marie Paule Kieny, Reza Malekzadeh, Srinivas Murthy, Richard Peto (independent DSMC statistician), K Srinath Reddy, Mirta Roses Periago, Soumya Swaminathan.

National investigators and researchers (excluding Steering Committee members) – co-authors

Albania: University Hospital Centre Mother Theresa, Tirana Nevila Gjermeni MD, Esmeralda Meta PhD.

Argentina: Health Ministry Juan Balbuena MD, Juan M Castelli MD, Analia Mykietiuk MD, Carla Vizzotti MD; Hospital Centenario, Rosario Damian Aguila MD, Sergio Lupo, MD; Hospital de Infecciosas Buenos Aires Viviana Chediack MD, Eleonora Cunto MD, Lautaro de Vedia MD, Cecilia Domínguez MD, José Fernández MD, Nicolás Lista MD, Alejandra Rodríguez MD, Francisco J Muñiz; Hospital General de Agudos José Ramos Mejía, Buenos Aires Sabrina Caimi MD, Carolina Delgado MD, Pablo Frare MD, Marcelo Losso MD, Florencia Masciottra MD, Valeria Pachioli MD, Javier Toibaro MD; Hospital General de Agudos Juan A Fernández, Buenos Aires José Barletta MD, Juan Carrillo MD, Nicolas D'Amico MD, Laura Hermida MD, Martin Jaume MD, Cecilia Luna MD, María José Padilla MD, Jazmin Patroso MD, Luz Perez Blanco MD, José Louis Presas MD, María José Rolon MD, Alicia L Sisto MD, Sandra Themines MD; Hospital Julio C Perrando, Resistencia, Chaco Veronica Arce MD, Patricia Arribillaga MD, Roxana A Ferreyra (nurse), Maria L Lescano MD, Fernando Tito MD, Luciano Verón MD; Hospital Mariano y Luciano de la Vega, Moreno Angelo Chalco MD, Javier Farina MD, Matias Provenzano MD; Hospital Nacional Profesor Alejandro Posadas, Palomar Ignacio Alonso MD, Rodrigo Alzola MD, Maria Benedetti MD, Flavia Cervellino MD; Debora Di Pilla MD, Pablo Díaz Aguiar MD, Leonor Ellero MD, Celeste Giudiche MD, Mariana Golikow MD, Mariela Jacobo MD, Diego Laplume MD, Flavia Loiacono MD, A Ana Belén López MD, Cecilia Pallavicini MD, Florencia Riveros MD, Graciela Torales MD; Hospital Prof Bernado Houssay, Vicente López, Marcos Altamirano MD, Laura Barcelona MD, Verónica Berdiñas MD, Carolina Fogar MD, Anabella Martin MD; Hospital Provincial Dr José María Cullen, Santa Fé Rafael Avila MD, Julieta Burgui Pharm, Juan Carrizo MD, María Filippi MD, Maria Georgina Gomez MD, Viviana Reichert MD; Hospital Rawson, Córdoba María Alvarez MD, Alexis Cazaux MD, Miguel Díaz MD, Mariano Hurtado MD, Leonardo Marianelli MD, Lorena Orellano MD, Lorena Lavera MD, Carolina Salvay MD, María Simonetta MD.

Austria: Through DISCOVERY add-on study. Landeskrankenhaus Salzburg Universitätsklinikum, Salzburg Alexander Egle MD, Richard Greil MD; Medizinische Universität Innsbruck, Innsbruck Michael Joannidis MD.

Belgium: Through DISCOVERY add-on study. Service d'infectiologie, CHR de la Citadelle, Liège Antoine Altdorfer MD, Vincent Fraipont MD; Cliniques Universitaires de Saint Luc, Bruxelles Leila Belkhir MD; Cliniques Universitairesde Bruxelles-Hôpital Erasme, Bruxelles Maya Hites MD.

Brazil: Fundação Universidade de Pernambuco Democrito B Miranda Filho PhD, Poliana Monteiro PhD; Hospital Couto Maia VPS Almeida, Ceuci X Nunes PhD; Hospital das Clínicas da Universidade Federal de Minas Gerais Helena Duani PhD; Hospital das Clínicas, Universidade Federal do Paraná Giovanni L Breda PhD, Sonia M Raboni PhD; Hospital Estadual de Sumaré Arthur JS Colussi MD, Marcelo C Ramos MD, Leonardo F Ruffing PhD; Hospital Federal do Estadodo Rio de Janeiro Esaú C João PhD; Hospital Regional de Mato Grosso do Sul Julio H Croda PhD; Hospital Regional de São José Gustavo A Pinto PhD; Hospital São José de Doenças Infecciosas Erico AG Arruda PhD; Hospital Sírio Libanês Miran Corradi PhD; Hospital Universitário Clementino Fraga Filho Elizabeth S Machado PhD, Fernanda CQ Mello PhD; Instituto de Infectologia Emilio Ribas Luiz Carlos Pereira Junior PhD, Tamara NL Souza MSc; Oswaldo Cruz Foundation Valdilea GV Santos PhD; Universidade Federal de Santa Maria Alexander Schwarzbold PhD.

Canada: Centre Hospitalier de l'Universite de Montreal Francois Martin-Carrier MD, Medeline Durand MD, Christophe Kolan MD; Centre Hospitalier Universitaire de Sherbrooke Fracois

Lamontagne MD; CHU de Quebec-Universite Laval David Bellemare BSc, Eve Cloutier Bsc, Tuong -Vi Tran MD, Alexis Turgeon MD; Grey Nuns Community Hospital Holly Hoang; Hôpital du Sacré-Coeur de Montréal Yiorgos Alexandros Cavayas MD ; Hopital Montfort Nicholas Chagnon MD; Lions Gate Hospital James Douglas MD; Markham Stouffville Hospital, Evadiki Fera MD; McGill University, Montreal Matthew P Cheng MD, Cecilia Costiniuk MD, Luke Harrison MD, Kosar Khwaja MD, Marina Klein MD, Nadine Kronfli MD, Todd C Lee MD, Jesse Papenburg MD, Makeda Semret MD; McMaster university Erick Duan MD; Memorial University of Newfoundland Tanweer Azher MD; Misericordia Community Hospital Matthew Munan MD; Misericordia Hospital - Covenant Health Kim Scherr MD; Niagara Health Karim Ali MD, George Farjou MD, Jennifer Tsang MD; North York General Hospital, Toronto Alexandra Lostun MD, Anna Geagea MD; Ottawa Hospital Shane English MD; Queen's University Santiago Perez-Patrigeon MD; Queensway Carleton Hospital Moira Rushton MD; Royal Alexandra Hospital Ameeta Singh MD ; Sinai Health System, Toronto Mike Fralick MD; St. Michael's Hospital Darrell Tan MD; St Paul's Hospital N Press; Sunnybrook Hospital Nick Daneman MD, Catarina Downey BSc, Rob Fowler MD, Asgar Rishu MBBS; Ottawa Hospital Marlee McGuinty MD; Thunder Bay Regional Health Centre David Gregory Gamble MD; University of Alberta Erika MacIntyre MD, Conar O'Neil MD, Wndey Sligl MD, Stephanie Smith MD, Nelson Lee MD; Trillium Health Partners C Graham; University Health Network, Toronto I Bogocj; University of Alberta N Lee, C O'Neil; University of British Columbia Jennifer Grant MD, Daniel Ovakim MD; University of Calgary, CalgaryJohn Conly MD, CD Fell, Rachel Lim MD, RAnjani Somayaji MD, Alain Tremblay MD, Erik Vakil MD, Jason Weatherald MD; University of Manitoba Lauren Kelly MD, Yoav Keynan MD, Ryan Zarychanski; Western University Seemanair Parvathy PhD, Michael Silverman MD; William Osler Health System Alexandra Binnie MD, Sergio Borgia MD, Thomas Havey MD.

Colombia: Clínica Colsanitas, Sede Clínica Iberomérica Ivan Zuluaga MD; Clínica Colsanitas, Sede Clínica Reina Sofia Julio Chacón MD, Diego Garzón MD, Fredy Guevara MD; Clínica Colsanitas, Sede Clinica Santa Maria del Lago Juan S Bravo MD; Clínica Colsanitas, Sede Clínica Sebastian de Belalcazar José M Oñate MD;Clínica Colsanitas, Sede Clínica Universitaria Colombia Silvia Lozano-González MD; José A Rojas- Gambasica MD, Carlos H Saavedra MD; Fundación Cardioinfantil-Instituto de Cardiología Eliana Váquiro-Herrera MSc, Fabio Varón-Vega MD; Fundación Hospital Universidad del Norte Hugo Macareno MD; Fundación Santa Fe de Bogotá Monica Caicedo MD; Fundación Universitaria Sanitas Claudia Aristizabal MD, Anita Montañez-Ayala Bsc; Fundación Valle de Lili Fernando Rosso MD; Hospital Universitario San Ignacio, Pontificia Universidad Javeriana Sandra L Valderrama MD.

Egypt: Ain Shams University Gehan Elassal MD; AL-Azhar University Sami Zaky MD; Assuit University Sahar Hassany MD, Ehab Moustafa MD; Cairo University, Akram Abdelbary MD, Noha Asem MD, Hossam Masoud MD, Ahmed Said MD; Ministry of Health and Population, Wagdi Amin MD, Marwa Elshesheny BDS, Mohamed Fathy PharmB, Naglaa Fathy MBBCh, Notaila Fayed MBBCh, Ahmed Hammam PharmB, Hamdy Ibrahim MD, Mohamed Solyman Kabil Masters, Maryam Mohamed PharmB, Abdullah Mohamed Gouda PharmB, Shaimaa Okasha PharmB, Ahmed Rafik MBBCh, Asmaa Sedky PharmB, SaraTarek PharmB, Ahmed Tharwat PharmB; National Hepatology and Tropical Medicine Research Institute Amin Abdel Baki MD; National Liver Institute Wael Abdel-Razek MD; National Research Center Ehab Kamal MD.

Ethiopia: Armauer Hansen Research Institute Emawayish Tesema Abegaz MD, Zelalem Mekonnen Bekele MD, Filmona Mekuria Asfaw MD, Netsanet Aragaw Tegegne MHP, Miheret Fikre Teklemariam MD, Frehiwot Tamiru Nigusse MSc, Daniel Legesse Achalu MSc; Eka Kotebe General Hospital Shewit Tesfagabr Weldegergs MD, Dawit Kebede Huluka MD, Addisu Birhanu Tereda MD.

Finland: Helsinki University Hospital, Helsinki Ville Holmberg PhD, Saana Horstia RN, Petrus Järvinen PhD, Ilkka Kalliala PhD, Tuomas Kilpeläinen PhD, Hanna-Riikka Kreivi PhD, Satu Lamminmäki PhD, Laura Mäkinen PhD, Jarkko Mäntylä MD, Tiina Mattila PhD, Marjukka Myllärniemi PhD, Juuso Paajanen PhD, Andreas Renner MD, Susanna Tuominen MD; Hyvinkää Hospital, Hyvinkää Mia Haukipää MD, Toni Jämsänen MD, Taina Nykänen PhD, Miro Nyqvist MD; Kanta-Häme Central Hospital, Hämeenlinna Iivo Hetemäki MD, Emma Reponen MD, Tuomas Rosberg MD; Kuopio University Hospital, Kuopio, Riitta-Liisa Patovirta PhD, Päivi Salonen MD, Katariina Sivenius PhD; Mikkeli Central Hospital, Mikkeli Ilari Kuitunen PhD; Oulu University Hospital, Oulu Tero Ala-Kokko PhD, Pia Holma MD, Heikki Kauma PhD, Terhi Partanen MD, Emmi Puusti MD; Porvoo Hospital, Porvoo Heikki Ekroos PhD; Seinäjoki Central Hospital, Seinäjoki Sari Risku PhD; Tampere University Hospital, Tampere Jutta Delany MD, Riina Hankkio PhD, Ville Jalkanen PhD, Juuso Järventie MD, Joni Niskanen MD, Erja-Leena Paukkeri PhD, Jarno Rutanen PhD, Petrus Säilä MD, Marjatta U Sinisalo PhD.

France: Through DISCOVERY add-on study. Amiens University, Amiens Claire Andrejak MD, Jean Philippe Lanoix MD,Yoann Zerbib MD; ANRS, Paris Alpha Diallo MD, Noemie Mercier MD; Centre hospitalier Andrée Rosemon, Cayenne, Guyane Felix Djossou MD; Centre Hospitalier Annecy Genevois, Annecy David Bougon MD, Violaine Tolsma MD; Centre Hospitalier Universitaire de Besançon, Besançon Kevin Bouiller MD, Jean Christoph Navellou MD; Centre Hospitalier Universitaire de Nantes, Nantes Benjamin Gaborit MD, Francois Raffi MD, Jean Reignier MD; Centre Hospitalier Universitaire Dijon-Bourgogne, Dijon Pascal Andreu MD, Lionel Piroth MD, Jean Pierre Quenot MD; Centre Hospitalier Universitaire Grenoble Alpes, Grenoble Olivier Epaulard MD, Nicolas Terzi MD; Centre Régional Universitaire de Nancy, Vandoeuvre Lés Nancy Francois Goehringer MD, Antoine Kimmoun MD; Centre Régional Universitaire de Nice, Nice Johan-Victor Courjon MD, Jean Dellamonica MD, Sylvie Leroy MD, Charles Hugo Marquette MD; Centre Régional Universitaire de Rennes, Rennes Fabrice Laine MD, Bruno Laviolle MD ; Georges Pompidou European Hospital, Paris Alexandre Buffet MD, Antoine Fayol MD, Jean-Sebastien Hulot MD, D Lebeaux MD, Marine Livrozet MD; Groupe Hospitalier de la région Mulhouse Sud Alsace, Mulhouse Olivier Hinschberger MD, Yoganaden Mootien MD; Groupe hospitalier La Pitié-Salpêtrière, Paris Julien Mayaux MD, Valerie Pourcher MD; Groupe Hospitalier Paris Saint Joseph, Paris Cédric Bruel MD, Benoît Pilmis MD; Henri-Mondor Hospital, Créteil Sebastien Gallien MD, Armand Mekontso Dessap MD; Hôpital Bichat, Paris Toni Alfaiate MD, Aline Dechanet MD, Axelle Dupont MD, Samira Laribi MD, Marie Capucine Tellier MD, Sarah Tubiana MD; Hôpital Bichat, Université de Paris, IAME, Inserm, Paris Drifa Belhadi MD, Lila Bouadma MS, Charles Burdet MD, Xavier Lescure MD, France Mentre MD, Nathan Peiffer-Smadja MD, Gilles Peytavin MD, Jean Francois Timsit MD, Yazdan Yazdanpanah MD; Hôpital Cochin, Paris Solen Kerneis MD, Marie Lachatre MD, Odile Launay MD; Hôpital deBicêtre, Le Kremlin Bicêtre Sami Figueiredo MD, Stephane Jauréguiberry MD; Hôpital Delafontaine, Saint Denis Jerome Aboab MD, Flora Crockett MD, Naomi Sayre MD; Hôpital d'instruction des armées Bégin, Saint Mandé Clement Dubost MD; Hôpital Marie Lannelongue, Le Plessis Robinson Jerome Le Pavec MD, Francois Stefan MD; Hôpital Saint-Antoine, Paris Karine Lacombe MD; Hôpital Saint-Louis, Paris Jean Michel Molina MD, M Noret MD; Hôpital Tenon, Paris Gilles Pialoux MD; Hospices Civils de Lyon, Lyon Jean Christoph Richard MD, Julien Textoris MD, Florent Wallet MD; Institut National de laSanté Et de la Recherche Médicale, Paris Christelle Delmas MD, Juliette Saillard MD; Lapeyronie University Hospital, Montpellier Kada Klouche MD; Lille University Hospital, Lille Karine Faure MD, Emmanuel Faure MD, Julien Poissy MD; Metz-Thionvillehospital, Ars-Laquenexy Rostane Gaci MD, Céline Robert MD; Montpellier University Hospital, Montpellier Vincent Le Moing MD, Alan Makinson MD; Pontchaillou University Hospital, Rennes Francois Benezit MD; Sorbonne Université, Inserm, Paris Dominique Costagliola MD; Strasbourg University Hospital, Strasbourg Raphael Clere-Jehl MD, Francois Danion MD, Ferhat Meziani MD, Vincent Poindron MD; Toulouse University Hospital, Toulouse Fanny Bounes MD, Guillame Martin-Blondel MD; Tourcoing Hospital, Tourcoing Vanessa Jean-Michel MS, Eric Senneville MD; Tours University Hospital, Tours D Garot MD; University Hospital Centre of Bordeaux,Bordeaux, Alexander Boyer

MD, Charles Cazanave MD, Didier Gruson MS, Denis Malvy MD; University Hospital of Martinique, Fort-de-France Cyrille Chabartier MD; University Hospital of Saint- Etienne, Saint-Etienne Elisabeth Botelho-Nevers MD, Amandine Gagneux-Brunon MD, Guillame Thiery MD; University Hospital, Reims Bruno Mourvillier MD; University Hospital, Rennes Claire Fougerou MD.

Honduras: Hospital Atlantida, la Ceiba Angel A Fiallos MD; Hospital Leonardo Martinez, San Pedro Sula Laura Erazo MD; Hospital Militar, Tegucigalpa Rosbinda Figueroa MD; Hospital San Felipe, Tegucigalpa Juan Jose Flores MD, Lesddyy Melendez MD; Instituto Cardiopulmonar, Tegucigalpa Carlos Aguilar MD, Wendy Moncada MD.

India: AIIMS, Bhopal Shubham Atal MD, Vaibhav Ingle MD, Rajnish Joshi MD, Sagar Khadanga MD, Avik Ray MD, Saurabh Saigal MD, Swati Sharma MBBS, Abhishek Singhai MD; AIIMS, Jodhpur, Pankaj Bhardwaj MD, Pradeep Bhatia MD, Jaykaran Charan MD, Nishant Chauhan MD, Naveen Dutt MD, Mahendra Garg DM, Vijaya Nag MD, Benhur Shadrach MD; AIIMS, New Delhi Richa Aggarwal MD, Dalim Kumar Baidya MD, Randeep Guleria DM, Choro Athipro Kayina MD, Souvik Maitra MD, Ankit Mittal MBBS, Neeraj Nischal MD, Manish Soneja MD, Kapil Dev Soni MD, Anjan Trikha MD, Naveet Wig MD; AIIMS, Rishikesh Gaurav Chikara MD, Paras Gupta MBBS, Ravi Kant MD,Vijay Krishnan MD, Bharathi Mohan MBBS, Prasan Panda MD; All India Institute Of Medical Sciences (Aiims), Jodhpur Avinash Babu MD; Apollo Hospitals, Greams Lane, Chennai Ramakrishnan Nagarajan MBBS, Bharatkumar Tirupakuzhi Vijayaraghavan MD, Ramasubramanian Venkatasubramanian MD; Apollo Speciality Hospitals, Vanagaram, Chennai Jayasree Krishnan MD, Ebenezer Rabindrarajan MBBS DNB, Krishnamoorthy Seetharaman MD, Suresh Kumar Dorairajan MD; Armed Forces Medical College, Pune Tilak Tejomurtula MD; Army Institute of Cardio Thoracic Sciences, Pune Gaurav Bhati MD, Vikas Marwah MD, Deepu Peter MD; B. J. Government Medical College & Sassoon General Hospital, Pune Rohidas Borse MD, Bharti Daswani MDPharm, Sujeet Divhare MDPharm, Dhananjay Ogale MD, Shashi Sangale MD, Murlidhar Tambe MD, Rakesh Waghmare MD; B.J. Medical College & New Civil Hospital, Ahmedabad Chetnay Desai MD, Devang Raval MD, Kamlesh Upadhyay MD; Bharati hospital, Pune Nishant Agrawal DM, Shivkumar Iyer MD, Kamini Reddy PharmD, Sujata Rege DNB, Jignesh Shah MD; BYL Nair Hospital, Mumbai Rakesh Bhadade MD, Rosemarie de Souza MD, Minal Harde MD; Chirayu Medical Collegeand Hospital, Bhopal Ajay Goenka MD, Aniket Goenka MD, Ashutosh Mangalgiri MS, Rohit Parate MD, Krishna Singh MD;Christian Medical College, Vellore Ooriapadickal CherianAbraham MD, Amith Balachandran MD, Thambu David Sudarsanam MD; Gandhi Hospital, Hyderabad Vinaya Sekhar Aedula MD, Thrilok Chander Bingi MD, Vaishnavi Jamalapuram MD, Hemanth Kalakuntla MD, Akhilesh Maurya MD, Rajarao Mesipogu MD, Kammili Nagmani MD, K Padma Malini MD, Tushara Rao MD, Ravindra Kumar Sudarsi MD, Dawood Suleman MD; GMERS Medical College & Hospital, Gotri, Vadodara Kedar Mehta MD, Parvati Patel MD, Chirag Rathod MD; Government Medical Collegeand New Civil Hospital, Surat Chetankumar Acharya MDPharm, Krishnakant Bhatt MD, Mayur Chaudhari MD, Vipul Chaudhary MD, Balusami Divakar MD Pharm, Amit Gamit MD, Sweta Gamit MD, Bansari Kantharia MD, Abhay Kavishvar MD, Mohmmedirfan Momin MD, Chetna Patel MD, Vitan Patel MD, Sunaina Patel MD, Harshad Patel MD, Ashwin Vasava MD, Mamta Verma MD; Government Medical College, Nagpur Sagar Khandare MD, Dipti Chand MD, Mrunalini Kalikar MD,Sajal Mitra MS, Uday Narlawar MD; Government Siddhartha Medical College, Vijayawada, Chakradhararao Rao MD, Durga Prasad MD, Kurada Venkata Seshaiah MD; ICMR- National AIDS Research Institute, Pune Rakhee Bangar M.Pharma Med, Kirti Chaudhari PGDCR, Abhijeet Kadam MD, Sayali Kalme MD, Suchit Kamble MD, Arundhati Kashikar BSc, Pranali Kerkar MBBS, Pranjali Kokate MPharm, Sumitra Krishnan MBBS, Megha Mamulwar MD, Suvarna Sane MPhil; Indian Council of Medical Research, New Delhi Nivedita Gupta MBBS PhD, Samiran Panda MD; Madras Medical College& Rajiv Gandhi Government General Hospital, Chennai Therani Rajan Ethirajan MD, Sudha Kulur Mukhyaprana MD, Gopalakrishnan Natarajan DM, Lakshmiarasimhan Ranganathan DM, Thasneem banu Subhan MD, Sudharshini Subramaniam MD, Damodaran Vasudevan MD, Rajendran Velayudham MD; Omandurar

Medical College & Hospital, Chennai Rangarajan Jayanthi FRCP, Jayavelu Komathi MD, Ayyasami Revathi MD; Pandit Deendayal Upadhyay Government Medical College, Rajkot Meghavi Bhapal MD, Shobha Misra MD, Anil Singh MD, Aarti Trivedi MD; PD Hinduja National Hospital and Medical Research Centre, Mumbai Umang Agrawal FNB, Zarir Udwadia FRCP; RCSM GMC CPRH, Kolhapur Arunita Paritekar MD, Girish Patil MD, Anushka Waikar MD; Sardar Vallabhbhai Patel Institute of Medical Sciences and Research, Ahmedabad Vyom Buch MBBS, Ashish Chandwani MBBS, Falguni Majumdar MD, Supriya Malhotra MD, Paltial Palat MD, Amee Pandya MBBS, Deep Patel MBBS, Dhara Roy MD, Sonal Shah MD, Nilay Suthar MD, Shiraj Talati MBBS; SMS Medical College & Hospital, Jaipur Abhishekh Agrawal MD, Sudhir Bhandari MD, Sunil Mahavar MD, Raman Sharma MD,Shrikant Sharma MD, Arjeet Singh MD; Voluntary Health Services-Infectious Diseases Medical Centre, Chennai N Kumarasamy FRCP, Poongulali Selvamuthu PhD; WHO-India, New Delhi Mohammad Ahmad MD, Madhur Gupta DM, Vimlesh Purohit MBA.

Indonesia: National Institute of Health Research and Development Annisa Rizky Afrilia MD, Dona Arlinda MSc, Rossa Avrina MEpid, Lois E Bang BSc, Sri Laning Driyah MD, Mila Erastuti BSc, Tetra Fajarwati MClinNut, Muhammad Karyana MPh, Nurhayati Nurhayati MPh, Cicih Opitasari MHA, Antonius Arditya Pradana BSc, Yenni Risniati MEpid, Retna Indah Sugiyono MPh, Nugroho Harry Susanto PhD, Armaji Kamaludi Syarif MPh, Aris Yulianto BSc; RS University Airlangga, Surabaya Muhammad Amin MD; RS University Udayana Bali IKA Somia, RS YARSI, Jakarta Indra Kusuma PhD; RS. Universitas Udayana Bali I Ketu Agus Somia PhD; RSJ Prof. Dr. Soerojo, Magelang Harli Amir Mahmudji MD; RSPAU Dr. Esnawan Antariksa, Jakarta Flora Sari MD; RSPI Prof. Dr. Sulianto Saroso, Jakarta Pompini Agustina Sitompul MD; RSUD Dr. Achmad Mochtar, Bukittinggi Deddy Herman MD; RSUD Dr. Moewardi, Solo Harsini Harsini PhD; RSUD Dr. Saiful Anwar, Malang Yani Jane Sugiri MD; RSUD Dr. Soetomo, Surabaya Soedarsono Soedarsono PhD; RSUP Dr. Hasan Sadikin, Bandung Yovita Hartantri MD; RSUP Dr. Kariadi, Semarang Sofyan Budi Raharjo MD; RSUP Dr. M. Djamil, Padang Irvan Medison MD; RSUP Dr. Sardjito, Yogyakarta Bambang Sigit Riyanto MD; RSUP Dr. Wahidin Sudirohusodo, Makassar Iraway Djaharuddin PhD; RSUP Fatmawati, Jakarta Aryan Djojo MD; RSUP H. Adam Malik, Medan Ade Rahmaini MD; RSUP Persahabatan, Jakarta Fathiyah Isbaniah MD; RSUP Prof. Dr. R. D Kandou Manado Agung Nugroho MD; RSUP Sanglah, Bali I Gede Ketut Sajinadiyasa MD; RSUPN Dr. Cipto Mangunkusumo, Jakarta Ceva Wicaksono Pitoyo.

Iran (Islamic Republic of): Ahvaz Jundishapur University of Medical Sciences, Ahvaz Fatemeh Amini MD,Sasan Moogahi MD, Mehran Varnasseri MD, Mohammed Jaafar Yadyad MD, Farid Yousefi MD; Alborz University of Medical Sciences, Karaj Zeinab Siami MD, Alireza Soleimani MD; Arak University of Medical Sciences, Arak Alireza Kamali MD, Behnam Mahmoodiyeh MD, Hossein Sarmadian, Daryanaz Shojaei MD, Saedeh Soltanmohammad MD; Babol University of Medical Sciences, Babol Masomeh Bayani MD, Soheil Ebrahimpour MD, Mostafa Javanian MD, Mahmoud Sadeghi Haddad Zavareh MD, Mehran Shokri MD; Golestan University of Medical Sciences, Gorgan Behnaz Khodabakhshi MD, Alireza Norouzi MD, Samaneh Tavassoli MD; Guilan University of Medical Sciences, Rasht Farahnaz Joukar MD, Lida Mahfoozi MD, Fariborz Mansour-Ghanaei MD, Aydin Pourkazemi MD; Isfahan University of Medical Sciences, Isfahan Atousa Hakamifard MD, Mehrdad Salahi MD, Kiana Shirani MD; Kermanshah University of Medical Sciences, Kermanshah Mandana Afsharian MD, Alireza Janbakhsh MD,Feizollah Mansouri MD, Ronak Miladi MD, Payam Mohamadi MD, Zeinab Mohseni Afshar MD, Babak Sayad MD, Maria Shirvani MD, Siavash Vaziri, Mohammad Hossein Zamanian MD; Mashhad University of Medical Sciences, Mashhad Mahnaz Amini MD, Fateme Barazandeh MD, Saied Hafizi Lotfabadi MD, Rozita Khodashahi MD, Mahnaz Mozdourian MD, Seyed Naeimeh Saberhosseini MD, Marzieh Saberi MD, Niloufar Saber- Moghaddam MD, Yalda Yazdanpanah MD; Mazandaran University of Medical Sciences, Sari Farhabg Baba Mahmoodi MD, Fatemeh Fallahpoor Golmaee MS; National Institute for Medical Research Development, Tehran Bita Mesgarpour MD; Qazvin University of Medical Sciences, Qazvin Amin Karampour MD, Somaieh

KianiMajd MD, Reza Najafipour PhD, Hamidreza Najari MD, Elham Zare Hoseinzade MD; Qom University of Medical Sciences, QomSeyed Yaser Foroghi Ghomi MD, Mohamad Reza Ghadir MD, Mina Gheitani MD, Shima Sadat Hashemi Madani MD, Javad Khodadadi MD; Saveh University of Medical Sciences, Saveh Atefehsadat Akhavi Mirab MD, Mehdi Mesri MD, Seyyed Hassan Mozaffar MD; Shahid Beheshti University of Medical Sciences, Tehran Parvaneh Baghaei MD, Farzaneh Dastan PhD, Payam Tabarsi MD; Shahid Sadoughi University of Medical Sciences, Yazd Seyec Alireza Mousavi Anari MD; Shiraz University of Medical Sciences, Shiraz Mohammad Javad Fallahi MD, Mohsen Moghadami MD, Shoeleh Yaghoubi MD, Farid Zand MD; Tabriz University of Medical Sciences, Tabriz Khalil Ansarin MD, Haleh Mikaeili MD, Masoud Nazemiyeh MD, Ali Taghizadieh MD; Tehran Universityof Medical Sciences, Tehran Sareh Eghtesad PhD, Fereshteh Ghiasvand MD, Hamed Hosseini MD, Nasim Khajavirad MD, Minoo Mohraz MD, Hossein Poustchi PhD, Anahita Sadeghi MD, Mohammad Ali Sahraian MD, Mohammad Reza Salehi MD, Ali Reza Sima MD.

Ireland: Beaumont Hospital and Royal College of Surgeons in Ireland Eoghan deBarra MD; Cork University Hospital Corinna Sadlier FRCPI; Department of Health Teresa Maguire PhD; Mater Misericordiae University Hospital and School of Medicine, University College Dublin Aoife Cotter PhD, Eavan Muldoon MD; Mercy University Hospital Arthur Jackson FRCPI; St James's Hospital and Trinity College, Dublin Colm Bergin FRCPI; St Vincent's University Hospital and School of Medicine, University College, Dublin Cormac McCarthy FRCPI; University Hospital Galway and National University of Ireland Galway John G Laffey FCAI.

Italy: AOU Citta della Salute e Scienza, Torino Silvia Corcione MD, Francesco Giuseppe De Rosa MD, Silvia Scabini MD; ASST di Monza, Ospedale San Gerardo, Monza Luca Bisi MD, Paolo Bonfanti MD, Giulia Gustinetti MD, Francesca Iannuzzi MD; ASST Fatebenefratelli Sacco, Milano Amedeo Capetti MD, Massimo Galli MD, Stefano Rusconi MD; ASST Santi Paolo e Carlo, Milano Francesca Bai MS, Antonella d'Arminio Monforte MD, Esther Merlini MD; ASST Valtellina e Alto Lario, Ospedale di SondaloElisabetta Menatti MD, Patrizia Zucchi MD; Azienda Ospedaliera Ospedali Riuniti Marche Nord, Pesaro Francesco Barchiesi MD, Benedatta Canovari MD; Azienda Sanitaria Universitaria Friuli Centrale, Udine Davide Pecori MD, Carlo Tascini MD, Paola Della Siega MD, Maria Merelli MD; Azienda Socio Sanitaria Territoriale di Cremona Nicolo Cocco MD, Bruno Drera MD,Chiara Fornabaio MD, Angelo Pan MS; Brescia Spedali Civili General Hospital Francesco Castelli MD, Emanuele Focà MD, Eugenia Roldan MD; Fondazione IRCCS Ca' Granda Ospedale Maggiore Policlinico, Milano Alessandra Bandera PhD, Andrea Gori MD; Fondazione Policlinico Universitario A. Gemelli IRCCS, Roma Roberto Cauda MD, Antonella Cingolani, Katleen de Gaetano Donati MD, Silvia Lamonica MD; IRCSS Ospedale Sacro Cuore – Don Calabria,Negrar Di Valpolicella (Verona) Andrea Agheben MD, NiccolòRiccardi MD, Paola Rodari MD; Ospedale Cardinal Massaia, Asti Maria Degioanni MD, Tommaso Lupia MD; Ospedale Maggiore, Trieste Stefano Di Bella MD, Donatella Giacomazzi MD, Roberto Luzzati MD; Ospedale Policlinico San Martino – IRCCS, Genova, Matteo Bassetti PhD; Ospedale SM Goretti, Latina Blerta Kertusha MD, Miriam Lichtner MD, Paola Zuccalá MD; Policlinico di S. Orsola, Bologna Caterina Campoli MD, Pierluigi Viale MD; ULSS9 Scaligera, Legnago (Verona) Pierangelo Rovere MD, Marcello Vincenzi MD; University of Campania, Luigi Vanvitelli, Napoli Federica Calò MD, Nicola Coppola MD, Margherita Macera MD, Caterina Monari MD; University of VeronaEleonora Cremonini PhD, Pasquale De Nardo MD, Diletta Pezzani PhD.

Kuwait: Infectious Diseases Hospital Moudhi Al-Roomi MD, Kelly Schrapp MD; Kuwait University Salman Al-Sabah MD.

Lebanon: Centre Hospitalier Universitaire, Notre Damedes Secours Madonna Matar MD; Hotel Dieu de France Moussa Riachi MD; Lebanese American University Medical Center-Rizk Hospital Anna Farra MD; Mount Lebanon Hospital Nadine Yared MD; Rafic Hariri University Hospital Oussaima Dbouni MD, Mahmoud Hassoun MD, Michele Saliba MD.

Lithuania: Vilnius University, Institute of Clinical Medicine; Vilnius University Hospital Santaros klinikos, Vilnius Birute Zablockiene PhD.

Luxembourg (through DISCOVERY add-on study): Centre Hospitalier de Luxembourg Jean Reuter MD,Therese Staub MD.

Malaysia: Institute for Clinical Research - National Institute of Health Chun Keat Chew MPharm, Pik Pin Goh FRCS, WenYao Mak MPharm; Kuala Lumpur Hospital Chee Loon Leong MMed; Melaka Hospital NorZaila Zaidan MMed; Queen Elizabeth Hospital Heng Gee Lee MRCP; Sarawak General Hospital Hock Hin Chua MRCP; Sultanah Bahiyah Hospital Lee Lee Low MRCP; SungaiBuloh Hospital Yasmin G Mohamed Gani MMed; Tengku Ampuan Afzan Hospital Dzawani Muhamad MMed; Tuanku Fauziah Hospital Suhaila Ab Wahab MMed.

Mali: Center for Vaccine Development - CVD Mali Camilla Ducker MSc.

North Macedonia: University Clinic of Infectious Diseases and Febrile Condition Ilir S Demiri PhD.

Norway: Akershus University Hospital Olav Dalgard PhD; Diakonhjemmet Hospital Leif Vinge PhD; Haraldsplass Deaconess Hospital Bård Reikvam Kittang PhD; Haukeland University Hospital Bjørn Blomberg PhD; Innlandet Hospital, Elverum Carl Magnus Ystrøm MD; Innlandet Hospital, Lillehammer Ragnhild Eiken MD;Nord- Trondelag Hospital Trust Nina Vibeche Skei MD; Lovisenberg Hospital Hedda Hoel PhD; Molde Hospital Birgitte Tholin MD; Møre og Romsdal Hospital Dag Arne Lihaug Hoff PhD; Oslo University Hospital Anne Ma Dyrhol-Riise PhD, Alekxander Rygh Holten PhD, Trine Kåsine MD, Katerina Nezvalova-Henriksen PhD, Inge Christoffer Olsen PhD, Marius Trøseid PhD; Østfold Hospital Saad Aballi MD; Sorlandet Hospital, Arendal Roy Bjørkolt Olsen PhD; Sorlandet Hospital, Kristiansand Metter Haugli PhD; Stavanger University Hospital Åse Berg PhD; Telemark Hospital Hilde Skudal MD; Trondheim University Hospital Ranula Hannula MD; University Hospital of North Norway Anders Benjamin Kildal PhD; Vesfold Hospital Asgeir Johannessen PhD; Vestre Viken Hospital Trust, Bærum Anders Tveita PhD; Vestre Viken Hospital Trust, Drammen Lars Heggelund PhD; VestreViken Hospital Trust, Kongsberg Gernot Ernst MD; Vestre Viken Hospital Trust, Ringerike Lars Thoresen MD.

Oman: Armed Forces Hospital Issa Al Jahdhami MD, Khalid AlNaamani MD; Royal Hospital, Ministry of Health Zakariya Al Balushi MD, Nenad Pandak MD.

Pakistan: Agha Khan University Hospital, Karachi Dilshad Begum ScN, Syed Faisal Mahmood DABIM, Nosheen Nasir FRCP; Pakistan Institute of Medical Sciences, Islamabad Nasim Akhtar MBBS, Usman Walayat MBBS; Shaukat Khanum Memorial Cancer Hospital and Research Centre Salma Abbas MD, Sadia Hassan MPhil, Shahzaib Khan MBBS, Faisal Sultan MD; Shifa International Hospital Sumeyya Azam MS, Ejaz Ahmed Khaan MD; The Indus Hospital, Karachi, Fivzia Herekar FCPS, Anum Rahim MSc, Samreen Sarfaraz MRCP, Qurat-ul-Ain Shaikh FCPS.

Peru: Centro Médico Naval Karla Hortencia Bernal-Málaga MD, Keith Cayetano Marcelino Del-Aguila-Torres MD, Dauma Yesenia Gastiaburú-Rodriguez MD, Andrés Alonso Gomero-Lopez MD, Manuel Laca-Barrera MD, Claudia Ximena Peña-Mayorga MD, Jose Pro MD, Jorge Mauro Samanez-Pérez MD, Giannilu Michelle Sotomayor-Woolcott MD; Clínica Ricardo Palma Gonzalo Ernesto Gianella-Malca MD, Oscar J Ponce MD, Kory Mirtha Rojas-Murrugarra MD, Ruben Kevin Arnold Tapia-Orihuela MD; Clínica San Pablo Carla Vanessa Luna-Wilson MD, Fatima Josefina Ortega-Monasterios MD, Alejandro Peña-Villalobos MD; Hospital Cayetano Heredia Carla Raquel Cornejo-Valdivia MD, German Málaga MD, Fernando Mejía-Cordero MD; Hospital de la Amistad Perú Corea Santa Rosa II Jesus Alberto Juárez-Eyzaguirre MD, Franco

León-Jiménez MD; Hospital III Daniel Alcides Carrión-ESSALUDLuis Guillemo Barreto-Rocchetti PharmD, Neil Flores-Valdez MD, Miguel Angel Hueda-Zavaleta MD, Miguel Angel Inquilla-Castillo MD, Juan Arturo Mendoza-Laredo MD, Jimmy Pedro Otazú-Ybáñez MD, Katherin Estefania Ponte-Fernandez MD, Orlando José Vargas- Anahua MD; Hospital María Auxiliadora Ana Maria Alva-Correa MD, Bethsabé Ángeles-Padilla MD, Rosanna Andrea Franco-Vásquez MD, Roxana Consuelo Gallegos-Lópe MD,Marco Olivera-Chaupis MD, María Angélica Paredes-Moreno MD, Walter Torres-Ninapayta MD, Ruben Dario Vásquez- Becerra MD; Hospital Nacional Alberto Sabogal Sologuren Erika Cecilia Agurto-Lescano MD, Luis Enrique Hercilla-Vásquez MD, Carlos Alberto Iberico-Barrera MD, Carmen Sara Terrazas-Obregón MD; Hospital Nacional Daniel Alcides Carrión Jhuliana Castillo- Espinoza MD, Jesus Norberto Chacaltana-Huarcaya MD, Erika Díaz-Chipana MD, César Miguel Quispe-Nolazco MD, Manuel Efrain Ramos-Samanez MD, José Gabriel Vásquez-Cerro MD, Randi Mauricio Yauri-Lazo MD; Hospital Nacional Dos de MayoHugo Cesar Arbañil-Huamán MD, Claudia Vanessa Ibarcena-Llerena MD, Gonzalo Francisco Miranda-Manrique MD, Gabriela Santos-Revilla MD, Victor Francisco Terrones Levano MD, Cesar Eduardo Ticona- Huaroto MD, Dario Y Ugarte-Mercado MD; Hospital Nacional Hipólito Unanue Alonso Soto PhD; Hospital Nacional Hipólito Unanue Andrés Martín Alcantara-Díaz MD, Johan Alexander Azañero-Haro MD, Reynaldo Javier Carazas-Chavarry MD, Augusto Cruz Chereque MD, Ricardo Manuel Sánchez-Sevillano MD; Hospital Nacional Sergio E Bernales Indira Catalina Casimiro-Porras MD, Olivia del Carmen Peña- Vásquez MD, Epifanio Sánchez-Garavito MD, Hernan Sandoval-Manrique MD, Julio Antonio Silva-Ramos MD, Oscar M Torres-Ruiz MD; Hospital Regional Lambayeque Edison Dante Meregildo-Rodríguez PhD; Hospital Regional Lambayeque José Gustavo Alvarado- Moreno MD, Pool Christopher Ávila-Reyes MD, Jorge Marko A. Benitez-Peche MD, Liliana Norma Cabrera-Portillo MD, Halbert Chrostian Sánchez-Carrillo MD, Manuel Alberto Solano-Ico MD, Miguel Villegas-Chiroque MD; UniversidadPeruana Cayetano Heredia Paloma Mariana Cárcamo MD, Ricardo Marin MD, Anna Larson Williams MPH.

Philippines: Asian Hospital and Medical Center Lenora Fernandez MD , Marion Kwek MD; Baguio General Hospital Thea Pamela Cajulao MD; Batangas Medical Center Rozelle Jade Javier MD; Cardinal Santos Medical Center Mary Shiela Ariola Ramos MD, Lourdes Gonzales Santos MD; Cebu Doctors' University Hospital Mitzi Marie Chua MD, Gerard Garcia MD; Chinese General Hospital Kingbherly Li MD; Diliman Doctors Hospital Gilly May Europa MD, Daisy Tagarda MD,; Fe Del Mundo Medical Center Katha Ngo-Sanchez MD; Lung Center of the Philippines Virginia De los Reyes MD, Mary Claire Orden MD; Makati Medical Center Janice Caoili MD, Maria Tarcela Gler MD; Manila Doctors Hospital Silverose Ann Andales-Bacolcol MD, Marisse Nepomuceno MD, Dennis Teo MD; Manila Med-Medical Center Manila Evalyn Roxas MD, Bob M. Te, MD; Perpetual Succor Hospital - Cebu Peter Blanco MD, Ma. Bernadette Chua MD, Mercedes Mujeres MD; Research Institute for Tropical Medicine Jemelyn Garcia MD, Arthur Dessi Roman MD; San Juan de Dios Educational Foundation Hospital Ruel Dionisio Paez MD, Christine Ramos Penalosa MD,; San Lazaro Hospital, Jamie Arches MD, Arlyn Awing, Rontgene Solante MD, Duane Richard Ymbong MD; Southern Philippines Medical Center Inofel Chin MD, Aileen Lee MD, Kathryn Roa MD; St. Luke's Medical Center Global Mario Panaligan MD; St. Luke's Medical Center Quezon City Ryan Llorin MD, Jenny Mae Quinvista Yoon, Jane Suaco MD, Christopher John Tibayan MD, Gelza Mae Zabat MD; The Medical City Cybele Lara Abad MD, Emily Aventura MD, Jia An Bello MD, Jorge Francisco MD, Prof Mary Ann Lansang MD; University of the East Ramon Magsaysay Memorial Medical Center Justine Cabrera MD, Victor Catambing MD, Minette Claire Rosario MD; University of the Philippines Manila Fresthel Monica Climacosa MD-PhD, Maria Elizabeth Mercado MD; University of the Philippines - Philippine General Hospital, Mary Grace Astudillo MD, Marlon Arcegono MD, Jubert Benedicto MD Susana Buno MD, Aileen David-Wang MD, , Mark Ramon Victor Llanes MD, Anna Flor Malundo MD, Ralph Elvi Villalobos MD; Vicente Sotto Memorial Medical Center Mishelle Vonnabie Bala MD, Omar Khayyam Macadato MD, Maria Philina Pablo-Villamor MD; West Visayas University Medical Center Lina Casiple-Amsua MD, Marie Grace Dawn Isidro MD, Helmar Soldevilla MD; World Citi Medical Center Sheila Marie Reyes MD, Issa Rufina Tang MD.

Portugal: Hospital de Curry Cabral – CHULC Stepanka Baktova MD, Orlando Cardoso MD, Ana-Raquel Garrote MD, Sara Lino MD, Maria- José Manat MD, Helder Pinheiro PhD, Diana Póvoas MD, Freddy Ramirez MD, Diana Seixas MD.

Saudi Arabia: Al Noor Specialist Hospital Mekkah M Al Gethamy MD, A Naji MD; Dammam Central Hospital MS AL-Mulaify MD; King Faisal Specialist Hospital and Research Centre, Riyadh A Alrajhi MD,R Al Maghraby MD; King Khaled University Hospital, Riyadh N Alotaibi MD, F AlShaharani MD, A Al Sharidi MD, M Barry MD, L Ghonem MD; Ohud Hospital Al Madinah A Khalel MD, AM Kharaba MD; Prince Mohammed Bin Abdulaziz Hospital, Riyadh L Alabdan MD, MS AlAbdullah MD; Qatif Central Hospital A Al Shabib MD.

South Africa: 3 Military Hospital Cloete Janse van Vuuren MBChB; Charlotte Maxeke Academic Hospital Ismail Kalla PhD, Jacquie Venturas MBBch; Chris Hani Baragwanath Academic Hospital Colin Menezes PhD, Sarah Alex van Blydenstein MBBCh, Merika Tsitsi MBBCh, Michelle Venter MBChB; Groote Schuur Hospital Mark Mendelson PhD, Bianca Sossen MBChC, Aimee Lifson MBchB, Mark Mendlson PhD; Sefako Makgatho Health Sciences University Vongani L Maluleke MBChB, Nathi Mdladla MBChB, Maphosphane Nchabeleng MBChB; Wits Health Consortium University of the Witwatersrand Jacklyn Bennet MBchB, Nokuphiwa Mbhele MBChB, Thando Mwelase MBChB, Victoria Parker MBChB, Pauline Howell MBBCh; Helen Joseph Hospital Mohammed Rassool MBChB; Wits Reproductive Healthand HIV Institute Thesla Palanee-Phillips PhD; Inkosi Albert Lethuli Central Hospital Nombulelo Magua PhD; Nelson Mandela Academic Hospital Thozama Dubula MbChB; Pelonomi Regional Hospital Dewald Steyn PhD; Steve Biko Academic Hospital Paul Reeder PhD; Universitas Private Hopsital Shaun Maasdorp MBChB; University of KwaZulu-Natal Simangele Bengu BSN.

Spain: Araba University Hospital, Vitoria-Gasteiz Juan-Carlos Gainzarain-Arana MD, Miguel-Angel Moran-Rodriguez MD, Zuriñe Ortiz-De-Zarate-Ibarra MD, Joseva Portu-Zapirain PhD, Ester Saez-De-Adana MD; Complejo Asistencial de Segovia Eva Maria Ferreira Pasos MD; Complejo Hospitalario de Toledo Julio González Moraleja PhD, MP Toledano; Hospital Clínic-IDIBAPS, University of Barcelona, Barcelona , Felipe Garcia PhD,; Hospital Clinico San Carlos, UCM, IdISSC, Madrid Ana Ascaso MD, , Noemí Cabello-Clotet MD, Vincente Estrada PhD, Antonio Leone MD, Daniel Lozano-Martin MD, Maria Jose Nuñez Orantos MD, Ana Belen Rivas Paterna MD, Raquel Sandoval MSN,Emilio Vargas PhD; Hospital Clínico Universitario Lozano Blesa IIS Aragón, Zaragoza María José Esquillor-Rodrigo PhD, Jesús Guzmán PhD, José Ramón Paño-Pardo PhD, Carla Toyas-Miazza PhD; Hospital Comarcal de Blanes Eva Armero Garrigos PhD; Hospital Comarcal SantJaume de Calella Arturo Juan Arribas MD;; Hospital Consorcio General Universitario Valencia Miguel GarcíaDeltoro PhD, Francesco Puchades PhD,; Hospital de Manises Koen Jerusalem MD; Hospital de Mérida AM Pérez Fernández PhD; Hospital General de Tomelloso Afredo J Lucendo PhD, ; Hospital General Universitario de Alicante, Esperanza Merino MD, Hospital General Universitario de Elche, Alicante Felix Gutierrez PhD,; Hospital General Universitario Gregorio Marañón Juan Berenguer PhD, Cristina Diez PhD , Chiara Fanciulli MD; Hospital La Paz.IdIPAZ Jose Ramon Arribas PhD, Fernando de la Calle MD, Beatriz Díaz Pollán MD,; Hospital Puerta de Hierro Elena Muñez Rubio PhD,; Hospital Quirónsalud Córdoba Rafael Cuenca-Acevedo MD; ; Hospital Universitari Sagrat Cor Rosario Salas MD; Universitario Basurto,Iñigo Lopez Azkarreta MD, Josefa Muñoz Sanchez PhD, Victor Polo San Ricardo MD; Hospital Universitario de Badajoz Francisco F Rodríguez Vidigal PhD; Hospital Universitario de Cruces Ane Josune Goikoetxea Agirre MD; Hospital Universitario de Getafe Daniel Abad Pérez PhD, , Lucía Fernández de Orueta MD, Mariella Luengo López MD; Hospital Universitario de Jaén Carolina Alarcón-Payer BSc, Gerardo Pérez Chica MD; Hospital Universitario de Salamanca JA Martín Oterino PhD; Hospital Universitario Donostia Instituto de Investigación BioDonostia Maialen Ibarguren MD, Jose Antonio Iribarren MD, Miguel Angel Von Wichmann MD; Hospital Universitario Fundación Alcorcón Carlos Guijarro PhD, Maria Velasco PhD; Hospital Universitario Infanta Leonor Pablo Ryan PhD,Jorge Valencia PhD; Hospital Universitario Infanta Sofía Maria José Abenza MD, Barbara Pagán-

Muñoz PhD, Pilar Ruiz-Seco PhD; Hospital Universitario Río Hortegade Valladolid Julia Gómez Barquero MD; Hospital Universitario Son Espases Francisco Fanjul MD, Adrian Ferre MD, Maria Peñaranda PhD; Hospital Universitario Virgen de la Victoria, Málaga Enrique Nuño MD, Carmen Pérez-López MD; Hospital Universitario Virgen Macarena Maria Dolores Del Toro PhD, Jesus Rodríguez-Baño PhD; Hospital Universitario y Politécnico La Fe, Valencia Miguel Salavert Lletí MD; Hospital Universitario12 de Octubre, Madrid A Lalueza MD;; Ramón y Cajal Hospital, Madrid Sandra Chamorro Tojeiro MD, Begoña Monge-Maillo PhD; Instituto Universitario de Investigación Biosanitaria de Extremadura (INUBE) JF Masa PhD.

Switzerland: Campus SLB, Lindenhofgruppe Bern Andreas Bosshard MD, Jan Wiegand MD; Clinic of Infectiologyand Infection Control, Kantonsspital Baden Michael Greiner MD; Department of Internal Medicine, Kantonsspital Frauenfeld Salome Gastberger MD; Hôpital du Jura Tiago Castro MD, Yvonne Schmiedel; Hôpital du Valais Sion Nicolas Desbaillets MD, Stephane Emonet MD, Myriam Eyer MD, Pierre-Auguste Petignat MD, Elisabeth Schaefer MD, Elisavet Stavropoulou MD; Hôpital fribourgeois Fribourg Veronique Erard MD; Hôpital Riviera-Chablais Rennaz Francois Duss MD, Nicolas Garin MD; Hôpitaux universitaires de Genève Alexandra Calmy MD, Yvonne Flammer MD, Annalisa Marinosci MD,Virginie Prendki MD; Inselspital Bern Hansjakob Furrer MD, Maria Christine Thurnher MD; Kantonsspital Aarau Anna Conen MD, Sebastian Haubitz MD, Egon Isenring MD, Barbara Jakopp MD, Emily West MD; Kantonsspital Baden Andrée Friedl MD, Jonas Rutishauser MD, Benedikt Wiggli MD; Kantonsspital Olten Matthias Hoffmann MD, Markus Lampet PharmD, Rein Jan Piso MD; Lausanne University Hospital F Matthias Cavassini MD, Florian Desgranges MD, Aurelie Fayet-Mello PhD, David Haefliger MD, Veronique Suttels MD,Lorena van den Bogaart MD; Réseau hospitalier neuchâtelois Neuchâtel Olivier Clerc MD; Spital Thurgau AG,Kantonsspital Münsterlingen Rosamaria Fulchini MD, Yvonne Martin MD; Universitätsspital Basel Marcel Stoeckle MD.

Other collaborators in participating countries
Special recognition to all the research staff and medical teams in hospitals in

Argentina: Hospital Ramos Mejía Buenos Aires (S Timpano, RF Fernandez Deu, A Guida, H Pandullo, P Rossini, C Gestoso), and Hospital Rawson Córdoba (K Lassen, F Silva, C Toledo, A Zamora,L Zappia);

Austria: AGMT Arbeitsgemeinschaft Medikamentöse Tumortherapie, Salzsburg (S Esmaeilzadeh-Leithner, B Lamprecht, D Wolkersdorfer);

Belgium: Cliniques Universitaires de Bruxelles-Hôpital Erasme, Université Libre de Bruxelles, Bruxelles (Z Khalil);

Brazil: Oswaldo Cruz Foundation (B Grinsztejn, MA Krieger, N Lima);

Canada: Centre Hospitalier del'Université de Montréal (S Matte), CHU de Québec-Université Laval (O Costerousse), Eastern Regional Health Authority (P Daley), Grand River Hospital, McMaster University (S Giilck), Hôpital Charles-Le Moyne (G Poirier),Dr Evert Chalmers Hospital (Z Aslam), Humber River Hospital (S Go, K Mandelzweig, S Manocha), Institut universitaire de cardiologie et de pneumologie de Québec (F Lellouche), Interior Health, Royal Inland Hospital (C Marek), Island Health - Victoria, BC (F Auld), Kingston Health Sciences Centre/ Queen's University (B Antuna-Puente), Markham-Stouffvile Hospital (A Ladelfa, V Sales), Michael Garron Hospital (C Kandel, M Taylor), Niagara Health (L Patterson), North York General Hospital (E Arhanchiague, E Owen), Royal Inland Hospital (K Gupta, E Parfitt), Royal University Hospital /University of Saskatchewan (S Peermohamed), Royal Victoria Regional Health Centre (G DiDiodato), Scarborough Health Network (S John), St. Mary's General Hospital (B Guy), St. Paul's Hospital, Vancouver, Canada (N Press), Sunnybrook Hospital (P Kiiza E Shadowitz, R Pinto), Trillium Health Partners (C Graham), University Health Network, Sinai Health, University of Toronto (SM Poutanen), University Health Network, Toronto (I Bogoch), University of British Columbia (V Chaubey), University of British Columbia, Saint Pauls Hospital (W Connora), University of Calgary (C Fell, P Mitchell), University of Manitoba (A Heendeniya) and William Osler Health System (M Bagi);

Colombia: Clínica Iberoamerica (R Conreras, C Rebolledo), Clínica Reina Sofia, (M Choconta, L Martínez), Clínica Santa María del Lago (J de La Hoz, S Peñalosa, M Salazar, A Valencia), Clínica Universitaria Colombia (Y Gil,M Jiménez, A Montañez, O Córdoba), Clínica Sebastián de Belalcázar (A Muriel, J Villabon), Clínica Universitaria Colombia (Y Gill, M Jiménez), Clínica Universitaria Colombia Colsanitas (O Córdoba), Fundación Cardio infantil Instituto de Cardiología (LD Sáenz, JC Villar), Fundación Santa fe de Bogotá (S Bello), Fundación Valle del Lili (K Gómez, A Martínez, A Sotomayor, J Yara), Fundación Universitaria Sanitas (D Castro, M Isaza, P Marín, C Orjuela), Hospital Universitario San Ignacio (V Méndez, C Gómez), Hospital Universidad del Norte (S Aguilera), Ministry of Health (A Moscoso,F Ruiz), PAHO (L Ramírez, G Tambini), INVIMA (J Aldana, P Pulgarín); National University of Colombia (M Jimenez);

Ethiopia: Eka Kotebe General Hospital (EA Weldeab, FA Kenea, AB Seyoum, WT Beyene, MF Abeera, TA Belay);

Finland: Helsinki University Hospital (M Saalasti), Occupational HealthHelsinki (J Mustonen), Oulu University Hospital (S Säikiö), Päijät-Häme Central Hospital (A Külmäsu), Satakunta Central Hospital (R Uusitalo-Seppälä), Tampere University Hospital (V Virtanen, J Antonen, G Määttä, J Jouppila, R Komulainen) & University of Helsinki (C Nystén);

France: ANRS (J Balssa, C Birkle, C Cagnot, S Gibowski, E Landry, A le Goff S Le Mestre, D Lebrasseur, L Moachon, C Moins, C Paul, V Petrov-Sanchez, L Wadouachi), ANSES (C Semaille), Centre de Ressources Biologiques, Bichat Hospital (S Tubiana), Centre hospitalier universitaire de Martinique (A Cabie), Georges Pompidou European Hospital (JL Diehl), Hôpital Avicenne Paris (O Bouchad), Hôpital Bichat Paris (B Basli, A Chair, C Laouenan, J Level, M Schneider), Hôpital d'Instruction des Armées Bégin (C Ficko), Hospices Civils de Lyon (B Leveau), Infective Agents Institute Lyon (M Bouscambert-Duchamp, A Gaymard, V Icard, B Lina, F Morfin-Sherpa), INSERM (S Couffin-Cadiergues, E D'Ortenzio, H Esperou, B Hmaze, P Puechal), Inserm ANRS Villejuif ()Y Netzer, E Riault), Institut Paris (L Abel), Pontchaillou University Hospital (M Revest), Reims University Hospital (F Beni- Sadr), Rennes University Hospital (C Cameli, A Caro, MJ Ngo Um Tegue), Tours University Hospital (L Bernard), Université Bordeaux Inserm (A Gelley, L Moinot, L Wittkop), Université Paris Saclay Inserm (M Brossard, A Essat, M Ghislain), Université Sorbonne Inserm (L Beniguel, M Genin);

Honduras: Agencia de Regulación Sanitaria (F Contreras), Hospital Atlantida la Ceiba (M Juarez); Hospital Leonardo Martinez San Pedro Sula (J Samara), Hospital Militar Tegucigalpa (TL Moreno), Hospital San Felipe Tegucigalpa (E Cruz, H Rodriguez), Instituto Cardiopulmonar Tegucigalpa (N Maradiaga), National Autonomous University of Honduras (F Herrera, S Moncada, W Murillo), Secretaria de Salud de Honduras (A Flores, R Aplicano), and PAHO (P Huerta);

India: AIIMS Rishikesh (A Chauhan, M Singh), AIIMS New Delhi (S Bhatnagar, S Bhoi, L Dar, A Kumar, P Mathur, VP Meena, A Mohan, R Subramaniam, P Tiwari), AIIMS, Jodhpur (D Mathur), Apollo Hospitals Greams Lane Chennai (A Ahammed, E Elvira, K Krishnamurthy, P Parthasarathy, S Pavithra, S Solomon, S Swaminathan), Apollo Speciality Hospitals Vanagaram Chennai (S Hilda, J Swaminathan), Apollo Speciality Hospitals Vanagaram Chennai (G Gnanamuthu), Army Institute of Cardio Thoracic Sciences Pune (V Mangal), Bharati hospital Pune (S Palkar), BYL Nair Hospital Mumbai (A Bhamare, R Singh), Christian Medical College Vellore (KPP Abhilash, B Chacko, S Chandy, R Charles, T George, K Gunasekaran, E Inbarani, A Jacob, A Lenin, D Mathew, M Moorhty, R Moses, SC Nair, P Rupali, N Stanley, B Thangakunam, G Varghese, S Viggeswarpu), Gandhi Hospital Hyderabad (MD Ahmed, B Billa), Government Medical College Nagpur (P Agrawal, N Agrawal, S Bhelekar, M Faisal, P Gomase, P Gosavi, R Sabu), Indian Council of Medical Research New Delhi (S Agrawal), Madras Medical College Chennai (G Arathi, G Jayashree, T Meenakshi, S Gomathi), Omandurar Medical College Chennai (C R Anuradha, M Jayakumar, C Praveen Kumar, R Pravin Kumar, T Ramesh Kumar, S Sai Vishal, A Subashree); Omandurar Medical College & Hospital, Chennai KP Manimaran; PD Hinduja National Hospital and Medical Research Centre Mumbai (S Mehendale, R Raju, A Sunavala), SMS Medical College & Hospital Jaipur (B Gupta), VHS Infectious Diseases Medical Centre Chennai (F Beulah, S Ramu, N Govindarajan);

Indonesia: RSUP Prof. Dr. R.D. Kandou, Manado (E Prasetyo,FNK Fujiyanto, R Adiwinate, E Kristanto); RS Univ. Airlangga, Surabaya (HW Setiawan, PA Wulaningrum, AN Rosyid, NA Ramadhan); RS Univ. Udayana, Bali (CAW Purnamasidhi, IW Aryabiantara, IK Suyasa, DPGP Samatra); RS YARSI,Jakarta (D Bachtiar, E Sastria, M Rusmana, E Yuliana); RSJ Prof. Dr. Soerojo, Magelang (V Otifa, W Sabaan, S Sumawan, I Nopiasardani); RSPAU Dr. Esnawan Antariksa, Jakarta R (Sitepu, R Pratama, S Siswandi, P Parman), RSPI Prof. Sulianti Saroso, Jakarta (N Mariana, A Rusli, T Sundari,R Rosamarlina); RSUD Dr. Achmad Mochtar, Bukittinggi (S Suyastri); RSUD Dr. Saiful Anwar, Malang (R Tantular, UA Setyawan, A Christanto, N Ichsan); RSUD Dr. Soetomo, Surabaya (T Kusmiati,M Qibtiyah, A Bachtiar, A Febriani), RSUD Moewardi, Solo (YF Dewi, J Aphridasari, A Adhiputri. E Pramudyaningsih), RSUP Dr. Hasan Sadikin (AY Soeroto,B Andriyoko, R Winarni, NH Chairunnisa), RSUP Dr. Kariadi, Semarang (MAU Sofro, FN Kholis, N Farkhanah,T Handoyo), RSUP Dr. M. Djamil, Padang (R Russilawati, A Anggrainy, S Ermayanti, O Khairsyaf), RSUP Dr. Sardjito, Yogyakarta

(F Dayi, A Riswiyanti, I Trisnawati, NR Ananda), RSUP Dr. Wahidin Sudirohusodo, Makassar (A Nurulita, M Ilyas, N Lihawa,NA Tabri, N Mayasari), RSUP Fatmawati,Jakarta (LT Yudhorini, J Nasarudin, MA Taufik, L Utami), RSUP H. Adam Malik, Medan (M Muntasir, A Agustina, D Panjaitan, FR Ananda), RSUP Persahabatan, Jakarta (AC Byantoro, R Sutarto,AS Asmara, T Kusumaeni), RSUP Sanglah, Bali (IAJD Kusumawardani, NW Candrawati, NLPE Arisanti, IMS Utama), RSUPN Dr. Cipto Mangunkusumo, Jakarta (A Susilo, G Singh)

Italy: Azienda Ospedaliera Integrata of Verona and the Servizio di Farmacia, Azienda Ospedaliera Integrata, Verona (P Marini, M Cesca, I Bolcato, L Scardoni);

Lithuania: Vilnius University, Institute of Clinical Medicine (M Paulauskas); Vilnius University Hospital Santaros klinikos, Vilnius (U Sakalauskiene);

Luxembourg: Clinical and Epidemiological Investigation Center, Strassen (M Alexandre), Hôpitaux Robert Schuman, Luxembourg (M Berna)

Mali: Center for Vaccine Development - CVD Mali (F Diallo, C Okello, U Onwuchekwa);

North Macedonia: University Clinic of Infectious Diseases and Febrile Conditions (S Marinkovikj, B Petreska, K Spasovska);

Pakistan: Shaukat Khanum Memorial Cancer Hospital (R Khan), Shifa International Hospitals (A Sohail, M Rafique), The Indus Hospital, Karachi (A Rehman, M Hussain, S Mustafa);

Philippines: Asian Hospital and Medical Center (MIL Fernandez), Baguio General Hospital (MLF de Leon, RG Dagwasi), Batangas Medical Center (ML Almero, M Mercado), Cardinal Santos Medical Center (A Vergara), Cebu Doctors' University Hospital (F Repunte), Chinese General Hospital (SO Tan, MK Ong-Tantuco, RC Reyes, PLG Co, ALG Gabriel-Chan, AO Reyes-Addatu, JT Li-Yu, SA Ang), Diliman Doctors Hospital (KI Del Ayre), Fe Del Mundo Medical Center (SMA Santos, A Torrico), Lung Center of the Philippines (H Basobas, Z Del rosario), Makati Medical Center (KM Taladua, HF Ricaforte-Docuyanan), Manila Doctors Hospital (JS Ramos- Precilla, SA Limson), Medical Center Manila (RC Genaro, TAE Nunez), The Medical City (A Santiago), Perpetual Succor Hospital, Cebu (TR Cuevas), Philippine Council for Health Research and Development (JC Montoya), Philippine General Hospital (JP Benedicto, M Llanes, G Astudillo, P Nala, R Abaya), Philippine Clinical Research Professionals (G Mendoza, J Arellano, AR Baniqued),Research Institute for Tropical Medicine (A Yabut), San Juan de Dios Educational Foundation Hospital (JD Cruz), San Lazaro Hospital (SM Ligutan), Southern Philippines Medical Center (EA Sibal), St. Luke’s Medical Center Global (G Dy-Arga, R Enecilla, CE Villavicencio, D David-Ona, S Unson, JD Gargar, BM Samonte), St. Lukes Medical Center Quezon City (AR Cumpas), University of the East Ramon Magsaysay Memorial Medical Center (MTF Sumagaysay), University of the Philippines (CDA Rozul, P Tagle, M Recana-Nieva), Vicente Sotto Memorial Medical Center (M Bagano, GM Aquino Jr, JD Bancat), West Visayas State University Medical Center (P Palmes, AJ Villaflor, H Soldevilla, MH Montalban, AM Jaen, EJ Berame, EV Razon-Gonzales, AA Gacutan-Liwag, M Altillero, PV Cruz), Department of Health Philippines (FT Duque III, MR Vergeire), Food and Drug Administration Philippines (RE Domingo), andWHO WPRO (JP Tonolete), and World Citi Medical Center (DJD Reotita);

Portugal: Centro Hospitalar e Universitário de Lisboa Central (CHULC) (M Ferreira), Agency for Clinical Research and Biomedical Innovation (AICIB) (F Luz);

Saudi Arabia: King Faisal Specialist Hospital and Research Centre, Riyadh (N Alorayyidh, R Moslmani), King Khaled University Hospital, Riyadh (A Abdurrahman, D Bintaleb);

South Africa: 3 Military Hospital (E Vorster), Charlotte Maxeke Johannesburg Academic Hospital (L Mabuza), Chris Hani Baragwanath Academic Hospital (D Kalambay, WBT Lechuti), Groote Schuur Hospital (S Koekemoer, S Moosa, T Morar), King Edward VII Hospital (S Matibela), Sefako Makgatho Health Sciences University (V Ramothwala, S Shaku), Steve Biko Academic Hospital (K Ma), Wits Health Consortium (C Barker, J Chellian, J Ferreira, Y Kilian, M Knight, L Koeberg, S Naidoo, A Rama, D Strydom), and Wits Reproductive Health and HIV Institute (R Boikanya, S Cornell, F Docrat, A Jacques, K Moodley, T Msomi);

Spain: Complejo Asistencial de Segovia (M.T. Criado Illana, P. Bachiller Luque, A. Carrero Gras, Lydia Iglesias Gómez, Paula Goicoechea Núñez); Complejo Hospitalario de Toledo (V Cano, J Largo Pau, MA Sepúlveda, MP Toledano), Hospital Clínic-IDIBAPS, University of Barcelona, Barcelona (A Carrillo, M Chumbita, L De La Mora, F Etcheverry, M Hernandez, A Inciarte, L Leal, O Miró, E Moreno, P Puerta, M Solà, A Soriano, A Tomé), Hospital Clinico San Carlos, UCM,IdISSC, Madrid (V Alvarez, O Astasio- Gonzalez, O Bueno, I BUrruezo, S Garica-Gomez, A Jimenez-Ortega, FJ Martin-Sanchez, C Perez-Ingidua, N Perez-Macias, MJ Tellez, D Uribe-Lopez), Hospital Clínico Universitario Lozano Blesa, IIS Aragón, Zaragoza (M de la Rica, L Díez-Galán), Hospital Comarcal de Blanes (P Pena Villanueva), Hospital Comarcal Sant Jaume de Calella (J Algarra Vento, Del Rio Pérez, A Marcia Paredes, D Pelleja Munné, A Razskeivicz, N Sabaté Frias, S Valero Rovira), Hospital Consorcio General Universitario Valencia (P Ortega, F Sanz, J Tamarit), Hospital General de Tomelloso (MI Elices-Calzón, J González-Cervera, G López-Larramona, MM Maestre-Muñiz, M Martin-Toledano, S Masegosa-Casanova, AM Ruiz-Chicote), Hospital General Universitario de Alicante (I Agea, V Boix, R García, J Gil, P Llorens, S Reus, R Sánchez, D Torrús-Tendero), Hospital General Universitario de Elche, Alicante (M Masia, S Padilla), Hospital General Universitario Gregorio Marañón (T Aldamiz, P Diez, I Gutiérrez, I Miguens, L Pérez-Latorre, M Ramirez), Hospital La Paz, Madrid (A Borobia, RM Torres), Hospital Puerta de Hierro (G Adolfo Centeno, AF Cabalero Bermejo, A Diaz De Santiago, A Fernandez Cruz, I Pintos Pascual, A Ramos Martinez), Hospital Regional Universitario de Malaga (MD Lopez-Carmona, I Perez-Camacho), Hospital Universitario 12 de Octubre, Madrid (L Pérez-Ordoño), Hospital Universitario Basurto (MM Alvarez Lavin, JM Baraiaetxaburu Artetxe, M De La Peña Trigueros, J De Miguel Landiribar, M Erburu Iriarte, O Ferrero Beneitez, S Ibarra Ugarte, M Intxausti Urrutibeaskoa, JI Larruscain Zorroza, I Lombide Aguirre, M López Martínez, R Martinez de Bourio Uriarte, C Moreno Muñoz, M Ramiz Martinez, M Rodriguez Miguel, A Sagarna Aguirrezabala, I Torre Salaberri, M Zubero Sulibarria), Hospital Universitario de Badajoz (JDD Arrebola Benítez, AM Castañar Jover, MN Nogales Muñoz), Hospital Universitario de Cruces (A Basterretxea Ozamiz, MJ Blanco Vidal, M Del Alamo Martinez, A García de Vicuña Melendez, M Ibarrola Hierro, J Isasi Otaolea, J Nieto Arana), Hospital Universitario de Getafe (E Aranda Rife, M Balado Rico, E Conde Senovilla, M Del Cerro Saélices, A Herrera Rodríguez, N López Muñoz, E Manzone, B Martínez Cifre, M Muñoz Flores, S Odeh Santana, G Pérez Caballero), Hospital Universitario de Jaén (MJ Barbero Hernández, C Herrero Rodríguez, F Horno Ureña, FJ La Rosa Salas), Hospital Universitario de Salamanca (J García Criado, M Marcos Martín), Hospital Universitario Donostia (E Agirre, I Alvarez, MC Andueza, A Berroeta, MJ Bustinduy, X Camino, A Couto, A Fuertes, MA Goenaga, X Kortajarena, JJ Zubeldia, B Zubelzzu, A Zufiaurre), Hospital Universitario Fundación Alcorcón (JJ Martínez-Simón, O Martin-Segarra, A Pablo-Esteban, G Sierra-Torres), Hospital Universitario Infanta Leonor (E Alvaro-Alonso), Hospital Universitario Infanta Sofía (I Fortuny-Esterri, R Garcia-Caballero, J Llorente Guitierrez, C Mansilla Paco, G Navarro Jimenez, A Nogales-García, I Sánchez-Rivero, MA Vázquez-Ronda), Hospital Universitario Son Espases (J Asensio, MI Fullana, L Ramon), Hospital Universitario Virgen de la Victoria, Málaga (R Jiménez-López, J Sánchez-Lora, E Sánchez-Yáñez), Hospital Universitario Virgen Macarena (M Gutiérrez-Moreno, I Jiménez-Varo, Z Palacios-Baena, N Palazón-Carrión, P Retamar, E Salamanca-Rivera, M Sevillano, A Valiente-Méndez, D Vicente-Baz), Hospital Universitario y Politécnico La Fe, Valencia (P Berrocal Gil), Ramón y Cajal Hospital, Madrid (Y Aranda García, P Borque, B Comeche, N Diaz Garcia, R Escudero-Sanchez, F Giogia, S Moreno Guillen, R Ron Gonzalez,

P Vizcarra), Hospital Universitario Virgen del Rocío, Sevilla (MA Lobo-Acosta, CM Rosso-Acosta);

Switzerland: Campus SLB Lindenhofgruppe Bern (C Groen, J Evison), Hôpital du Valais Sion (M Savet, A Luyet), Kantonsspital Aarau (K Bärtschi), Kantonsspital Baden (A Friedl, F Rutz), Kantonsspital Frauenfeld (P Rochat, P Hackman, P Wiesli, A Kistler, R Ursprung, S Danioth, R Werner, S Dias, M Schuster), Kantonsspital Münsterlingen (M Krause, D Vuichard, S Majer, D Rescigno, F Borer, A Meyer, M Köhler), Kantonsspital Olten (G Schenker), Hôpitaux Universitaires de Genève (P Vazquez, Y Gosmain), Lausanne University Hospital (L Vallotton, L Warpelin-Decrausaz, V Sormani, D Niksch, A Voidey, I Sommer), Réseau hospitalier neuchâtelois Neuchâtel (M Grosjean), and Hôpitaux universitaires de Genève et Faculté de Médecine (Clinical Research Center and HIV research team);

WHO teams: AFRO (C Garapo, JP Okeibunor), EMRO (A Hashish, C Kodama, A Mandil), EURO (C Butu, M Dara, A Kuli, A Mesi, N Mamulashvili, I Zurlyte), PAHO/AMRO (L Reveiz), SEARO (T Azim, M Gupta, R Takahashi), WPRO (A Cawthorne, YR Lo, JP Tonolette) and Headquarters (S Benitez, A Borges, T Bouquet, S Chuffart, E Egorova, R Embaye, S Kone, C Merle, P Molinaro, R La Rotta, N Mafunga, A Mazur, G Queyras).

Acknowledgments

The Ministries of Health of the participating Member States and their institutions provided critical support in the implementation of the trial.

Castor EDC donated and managed their cloud-based clinical data capture and management system. Anonymized data handling and analysis was at the Universities of Berne, Bristol and Oxford. Remdesivir was donated by Gilead Sciences, Hydroxychloroquine by Mylan, Lopinavir-Ritonavir by Abbvie, Cipla and Mylan and Interferon β-1a by Merck KGaA (subcutaneous) and Faron (intravenous). Add-on studies were conducted in Canada, France, India and Norway.

The chief acknowledgement is to the thousands of patients and their families who participated in this trial and made it possible, and to the hundreds of medical staff who randomised and cared for the patients.

Funding: Solidarity was supported by in-kind effort from many sources, and by:

Brazil: Oswaldo Cruz Foundation (Fiocruz); Evandro Chagas National Institute of Infectious Diseases (INI/Fiocruz); Laboratory of Clinical Research on Aids and STDs at Evandro Chagas National Institute of Infectious Diseases (INI/Fiocruz);

Canada: Canadian Institutes of Health Research; Vancouver Coastal Health Research Institute; Northern Alberta Clinical Trials and Research Centre; Covenant Health Research Centre; St. Joseph's Health Care Foundation; The London Health Sciences Foundation, London, Ontario; The Calgary Health Trust and Calgary Centre for Clinical Research COVID-19 Fund, and the Covenant Health Research Centre; The McGill University Health Centre was supported by a grant from the McGill Interdisciplinary Initiative in Infection and Immunity (MI4) with Funding from the MUHC Foundation;

Finland: Funding support from the Academy of Finland (309387)

France: EU-RESPONSE has received funding support from the European Union's Horizon 2020 research and innovation programme under grant agreement No 101015736;

India: the Indian Council of Medical Research under RFC No ECD/NTF-1-20-21; HETEROLABS Limited Hyderabad donated Remdesivir;

Iran: funding from the Iran Ministry of Health and Medical Education;

Ireland: The Department of Health, Republic of Ireland, in collaboration with the Health Research Board;

Italy: funding and in kind support from the Azienda OspedalieraIntegrata of Verona and the Servizio di Farmacia, Azienda Ospedaliera Integrata, Verona;

Malaysia: funding support from the Ministry of Health NIH Research Grant;

Norway: funding support from the Clinical Therapy Research in the Specialist Health Services (KLINBEFORSK);

Philippines: the Department of Science and Technology, Philippine Council for Health Research and Development; Department of Health

Portugal: Agency for Clinical Research and Biomedical Innovation (AICIB); the Ministry of Health

South Africa: the South African Medical Research Council with funds received from the Department of Science and Innovation;

Spain: the Spanish Clinical Research Network (SCReN, Institute of Health Carlos III, through PT17/0017/0018 - integrated in the State Plan I+D+I 2013-2016 and co-financed by FEDER), Research Project COV20/00612 (CTE-COVID-19, ISCIII), and the Agencia Española de Medicamentos y Productos Sanitarios (AEMPS); Hospital Universitario Virgen Macarena, Sevilla, Spain, has received funding support from the Instituto de Salud Carlos III, Spanish Network for Research in Infectious Diseases (REIPI; RD16/0016/0001), co-financed by European Development Regional Fund "A way to achieve Europe", Operative program IntelligentGrowth 2014-2020;

Switzerland: the Swiss National Science Foundation (NSF) in collaboration with the Federal Office of Public Health (FOPH).

WHO: supported through its COVID-19 Strategic Preparedness and Response Plan, including funding from the United Kingdom's Department for International Development (DFID, now replaced by the Foreign, Commonwealth & Development Office (FCDO); the German Federal Ministry of Health (BMG); the Ministry of Foreign Affairs of Denmark; the Kingdom of Saudi Arabia (the King Salman Humanitarian Aid and Relief Center); and the Government of the State of Kuwait.

Table S1. Treatment allocation vs death and/or initiation of ventilation

Ventilation includes invasive or non-invasive mechanical ventilation, or extra-corporeal membrane oxygenation (ECMO). Follow-up for mortality ceased at (first) discharge from hospital.

	Remdesivir vs its control		Hydroxy-chloroquine vs its control		Lopinavir vs its control		Interferon vs its control	
	**Active**	**Control**	**Active**	**Control**	**Active**	**Control**	**Active**	**Control**
**Not ventilated at entry**	n=3787	n=3782	n=864	n=818	n=1292	n=1253	n=2000	n=2011
Ventilation initiated after entry; died	242	251	29	19	52	46	142	113
Ventilation initiated after entry; lived	293	342	46	46	72	76	114	132
**Ventilation initiated after entry**	**535 14.1%**	**593 15.7%**	**75 8.7%**	**65 7.9%**	**124 9.6%**	**122 9.7%**	**256 12.8%**	**245 12.2%**
**P-value (from figure S5)**	**p=0.04**		**p=0.65**		**p=0.76**		**p=0.53**	
Never ventilated; died	209	258	40	42	65	71	105	102
**Died, or ventilation initiated after entry**	**744 19.6%**	**851 22.5%**	**115 13.3%**	**107 13.1%**	**189 14.6%**	**193 15.4%**	**361 18.0%**	**347 17.3%**
**P-value (from figure S7 analyses)**	**p=0.001**		**p=0.98**		**p=0.34**		**p=0.77**	
**Already ventilated at entry**	n=359	n=347	n=84	n=82	n=112	n=115	n=144	n=136
Died	151 42.1%	134 38.6%	35 41.7%	28 34.1%	34 30.4%	36 31.3%	69 47.9%	51 37.5%
P-value (from figure S7 analyses)	p=0.32		p=0.38		p=0.88		p=0.07	
**All patients**	n=4146	n=4129	n=948	n=900	n=1404	n=1368	n=2144	n=2147
**Died, or ventilation initiated after entry**	**895 21.6%**	**985 23.9%**	**150 15.8%**	**135 15.0%**	**223 15.9%**	**229 16.7%**	**430 20.1%**	**398 18.5%**
**P-value (from figure S7)**	**p=0.009**		**p=0.66**		**p=0.42**		**p=0.16**	

All p-values are stratified for age and, where relevant, for respiratory support at entry

Table S2. Numbers (and percentages) using selected non-study drugs

	Remdesivir vs its control		Hydroxychloroquine vs its control		Lopinavir vs its control		Interferon vs its control	
	Active N=4146	Control N=4129	Active N=948	Control N=900	Active N=1404	Control N=1368	Active N=2144	Control N=2147
Corticosteroids	2782<br>67.1	2820<br>68.3	190<br>20.0	197<br>21.9	367<br>26.1	387<br>28.3	1232<br>57.5	1286<br>59.9
Convalescent plasma	125<br>3.0	151<br>3.7	7<br>0.7	3<br>0.3	23<br>1.6	15<br>1.1	54<br>2.5	51<br>2.4
Anti-IL-6 medication	174<br>4.2	199<br>4.8	21<br>2.2	18<br>2.0	42<br>3.0	42<br>3.1	66<br>3.1	88<br>4.1
Non-trial interferon	5<br>0.1	30<br>0.7	1<br>0.2	1<br>0.1	4<br>0.3	0<br>0.0	2<br>0.1	31<br>1.4
Non-trial antiviral	115<br>2.8	262<br>6.4	76<br>8.0	73<br>8.1	111<br>7.9	111<br>8.1	122<br>5.7	179<br>8.3

Table S3. Multivariate analysis simultaneously estimating all 4 effects

The pre-planned primary analyses in the main text involved 4 pairwise comparisons (using log-rank methods), one between each treatment group and its controls, as indicated in the flowchart (Figure 1). These 4 primary analyses were stratified by age and by whether the patient was already ventilated at the time of randomisation, and found no definitely favorable or definitely unfavorable effect of any of the 4 study drugs on all-cause in-hospital mortality (Table 1, Figure S1). The RRs in these 4 pre-planned pairwise comparisons were:

Remdesivir vs its control (by pre-planned log-rank analysis) RR=0.91 (95% CI 0.82-1.02),

Hydroxychloroquine vs its control (by pre-planned log-rank analysis) RR=1.12 (0.84-1.48),

Lopinavir vs its control (by pre-planned log-rank analysis) RR=0.94 (0.75-1.18), and

Interferon vs its control (by pre-planned log-rank analysis) RR=1.21 (1.03-1.42, p=0.02).

As there was some overlap between the 4 control groups, an exploratory sensitivity analysis used multivariate Cox regression to fit all 4 treatment effects simultaneously, assuming the independence of any effects of lopinavir and of interferon. This multivariate analysis was stratified by the set of study drugs that was locally available at randomisation (13 occupied strata). Hence, no reduction of the dataset was needed to ensure that comparisons were only between concurrently randomised treatments, and that they were not subject to any selective biases. It was adjusted for several of the prognostic factors listed in Table 1: age (<40, 40-49, 50-59, 60-69, 70-79, 80+ years), sex, diabetes, bilateral lung lesions at entry (no, yes, not imaged at entry), and respiratory support at entry (no oxygen, oxygen but no ventilation, ventilation). This multivariate sensitivity analysis had not been pre-planned as a primary or a secondary analysis. For each of the 4 study drugs the multivariate analysis yielded RRs for active treatment vs local standard of care (SoC) that were similar to those in the pre-planned primary pairwise comparisons (except that the conventionall adverse effect of interferon ceased to be conventionally significant, again finding no definitely favorable or unfavorable effect of any of the 4 study drugs:

Remdesivir vs local SoC (by multivariate analysis) RR=0.93 (95% CI 0.84-1.04),

Hydroxychloroquine vs local SoC (by multivariate analysis) RR=1.09 (0.86-1.40),

Lopinavir vs local SoC (by multivariate analysis) RR=0.93 (0.76-1.14), and

Interferon vs local SoC (by multivariate analysis) RR=1.15 (0.99-1.34, p=0.06).

Figure S1A-S1D. Effects on in-hospital mortality of (A) remdesivir, (B) hydroxychloroquine, (C) lopinavir, and (D) interferon

Kaplan-Meier graphs, not standardised. Rate ratios (RR, 95% CI) include later in-hospital deaths, and standardise for age and ventilation at entry.

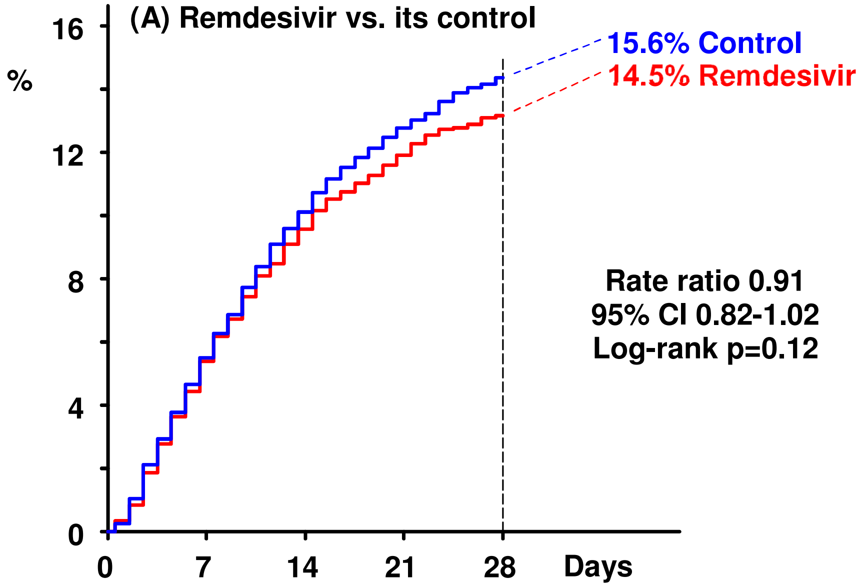

Weekly denominators (all not yet dead/lost), and numbers dying in hospital

Remdesivir	4146	222	3878	171	3703	96	3606	51	3554	62
Control	4129	225	3861	189	3665	108	3557	65	3490	56

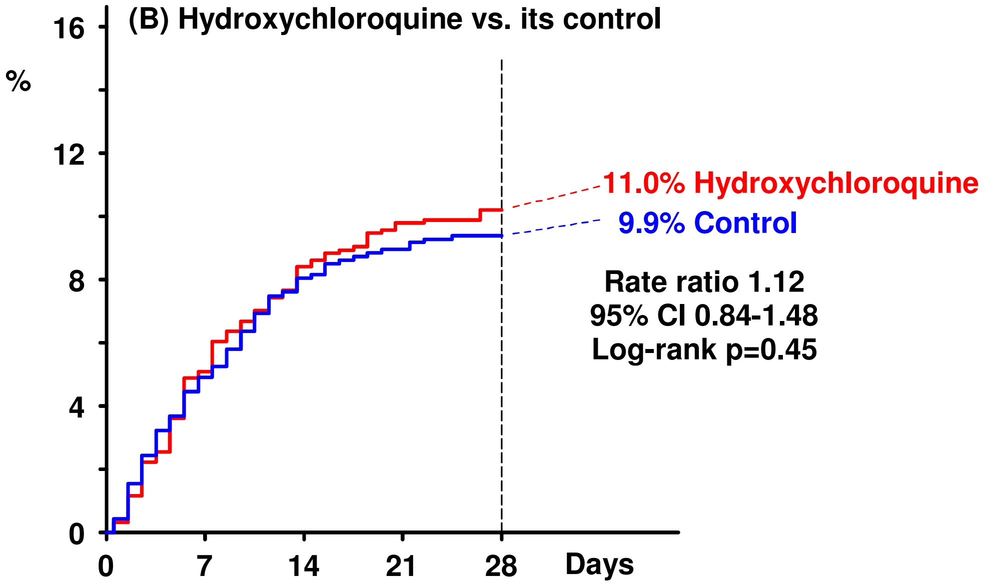

Weekly denominators (all not yet dead/lost), and numbers dying in hospital

Hydroxychlor.	948	48	890	31	858	13	845	4	841	8
Control	900	44	848	28	820	8	812	4	808	5

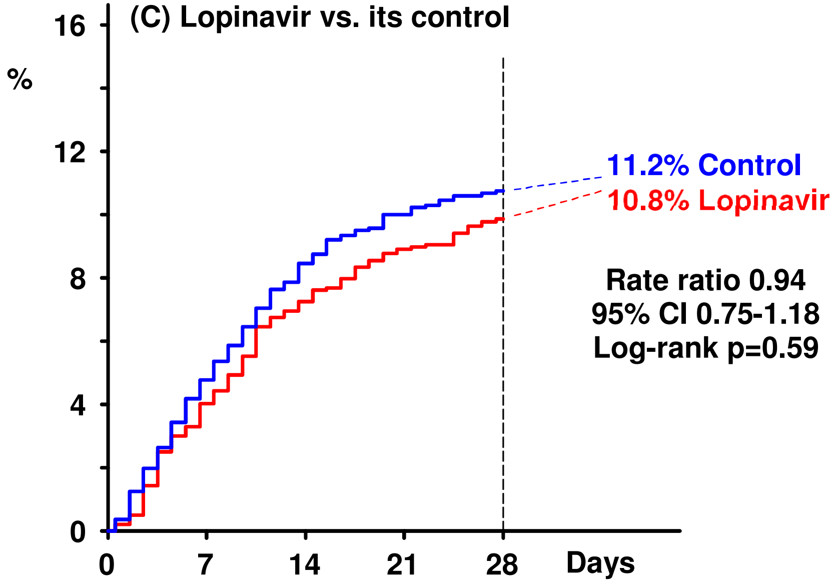

Weekly denominators (all not yet dead/lost), and numbers dying in hospital

Lopinavir	1404	56	1334	45	1286	23	1263	13	1250	14
Control	1368	65	1292	50	1242	21	1220	10	1208	7

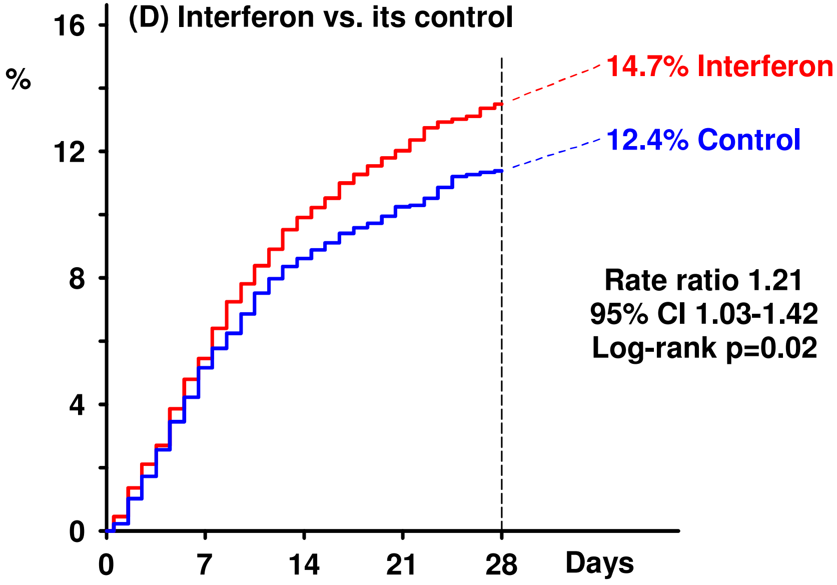

Weekly denominators (all not yet dead/lost), and numbers dying in hospital

Interferon	2144	116	1999	94	1904	45	1859	31	1828	30
Control	2147	110	2015	73	1939	35	1904	24	1880	24

Figure S2A. Subdivision by ventilation at randomisation of the apparent effects of remdesivir on the probability of death in hospital from any cause

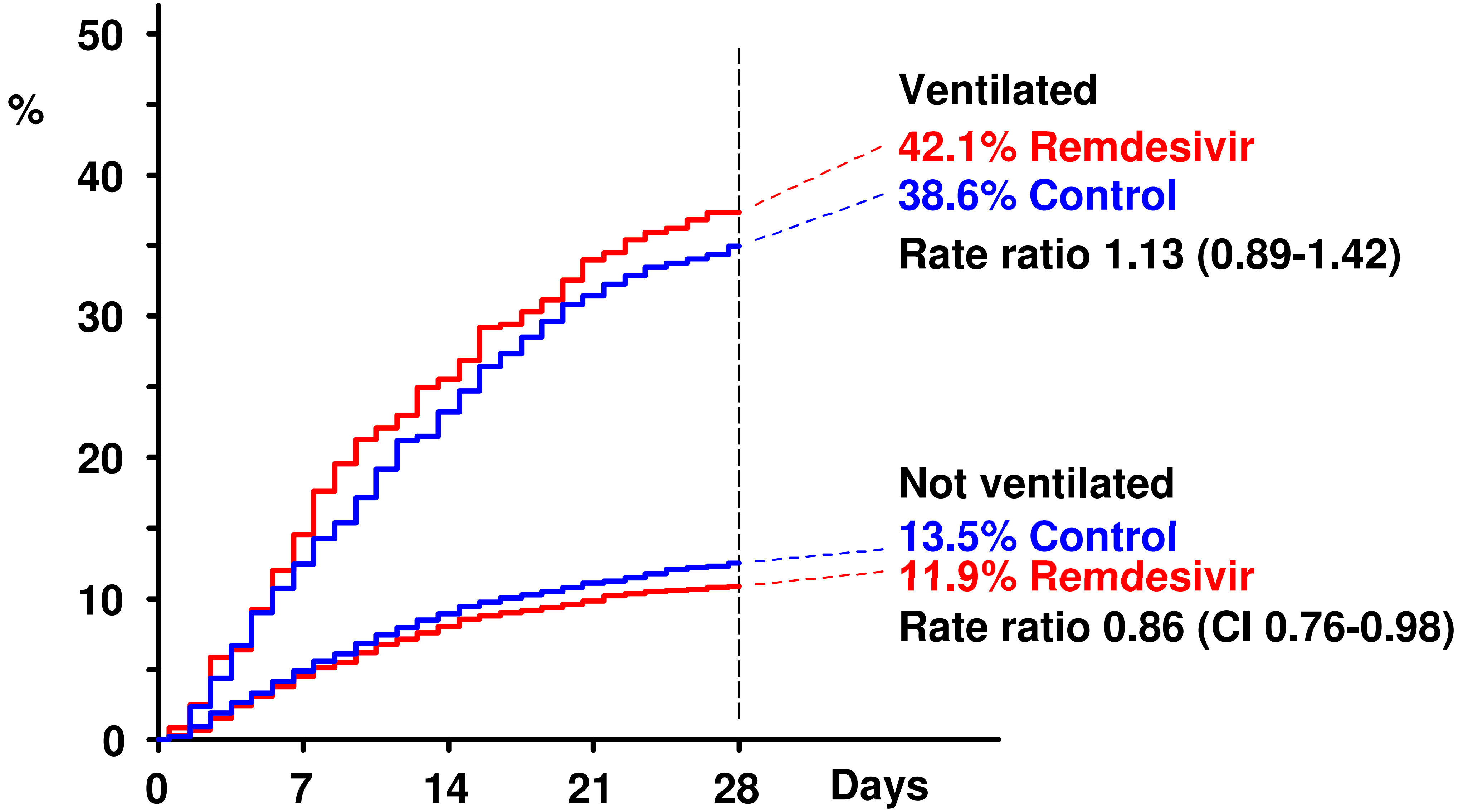

Weekly denominators (all not yet dead/lost), and numbers dying in hospital

Remdesivir	359	52	305	39	264	30	234	12	222	18	Ventilated
Control	347	43	301	37	263	28	235	12	222	14	
Remdesivir	3787	170	3573	132	3439	66	3372	39	3332	44	Not ventilated
Control	3782	182	3560	152	3402	80	3322	53	3268	42	

Figure S2B. Subdivision by ventilation at randomisation of the apparent effects of hydroxychloroquine on the probability of death in hospital from any cause

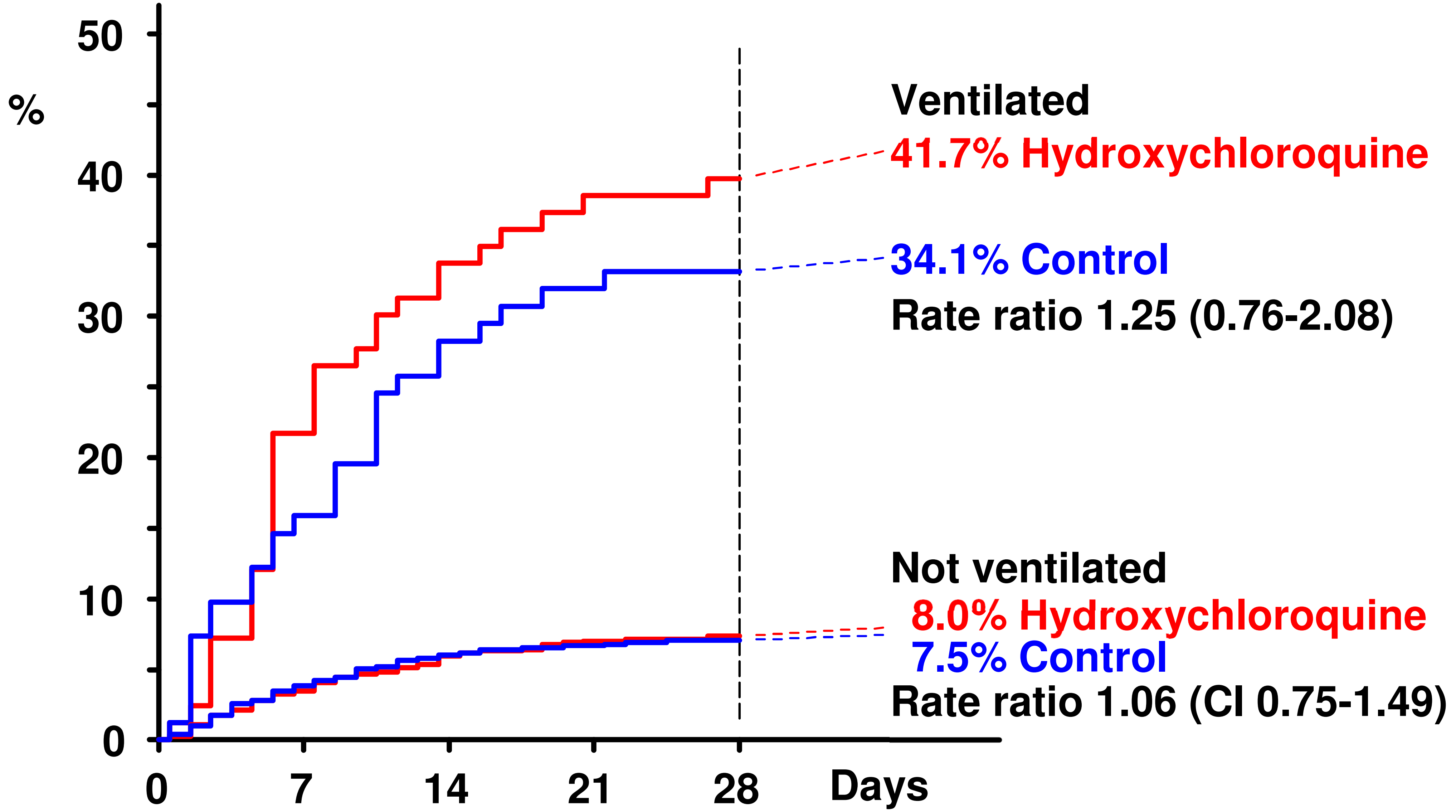

Weekly denominators (all not yet dead/lost), and numbers dying in hospital

Hydroxychlor.	84	18	65	10	55	4	51	1	50	2	Ventilated
Control	82	13	68	10	58	3	55	1	54	1	
Hydroxychlor.	864	30	825	21	803	9	794	3	791	6	Not ventilated
Control	818	31	780	18	762	5	757	3	754	4	

Figure S2C. Subdivision by ventilation at randomisation of the apparent effects of lopinavir on the probability of death in hospital from any cause

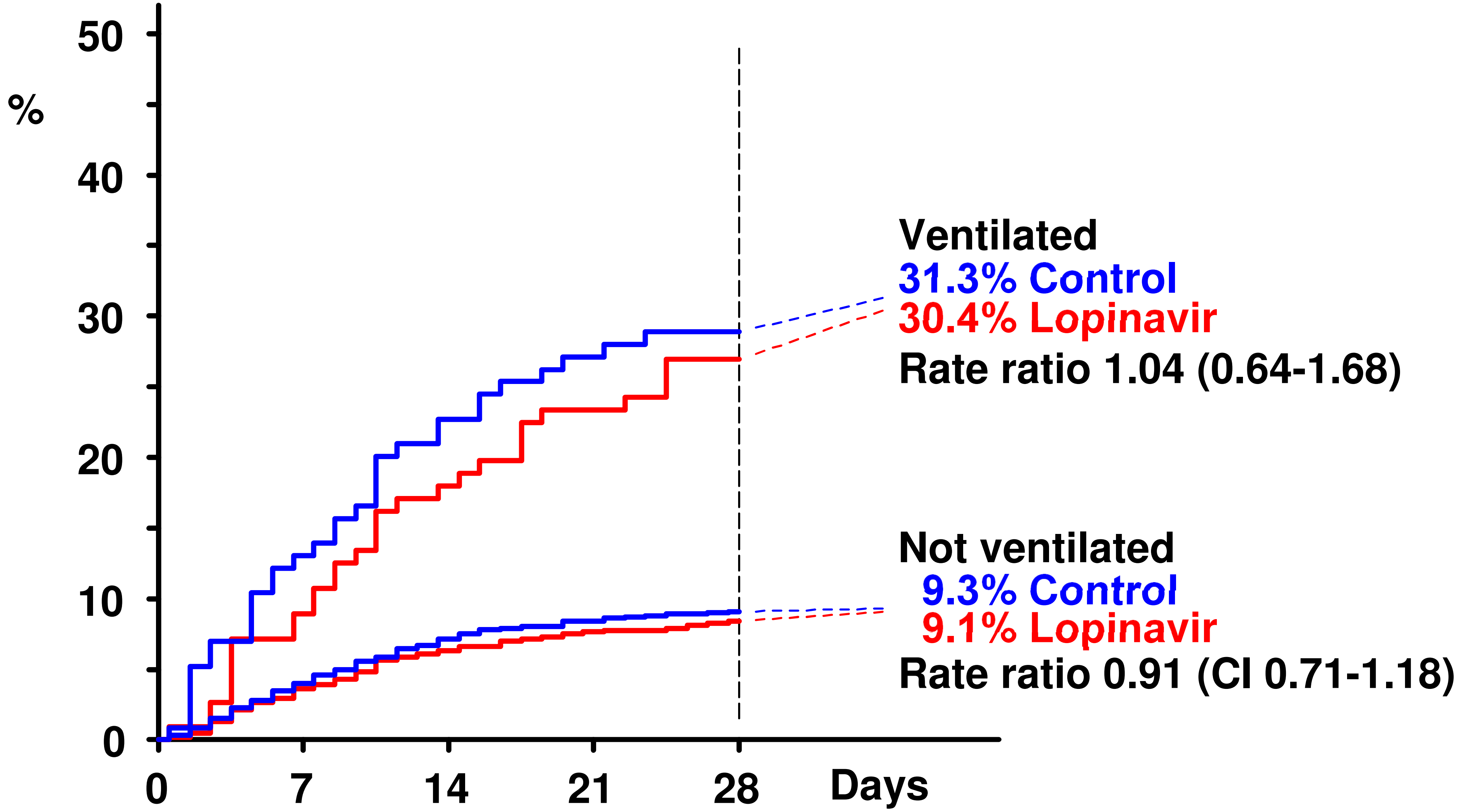

Weekly denominators (all not yet dead/lost), and numbers dying in hospital

Lopinavir	112	10	101	10	91	6	85	4	81	4	Ventilated
Control	115	15	99	11	88	5	83	2	80	3	
Lopinavir	1292	46	1233	35	1195	17	1178	9	1169	10	Not ventilated
Control	1253	50	1193	39	1154	16	1137	8	1128	4	

Figure S2D. Subdivision by ventilation at randomisation of the apparent effects of interferon on the probability of death in hospital from any cause

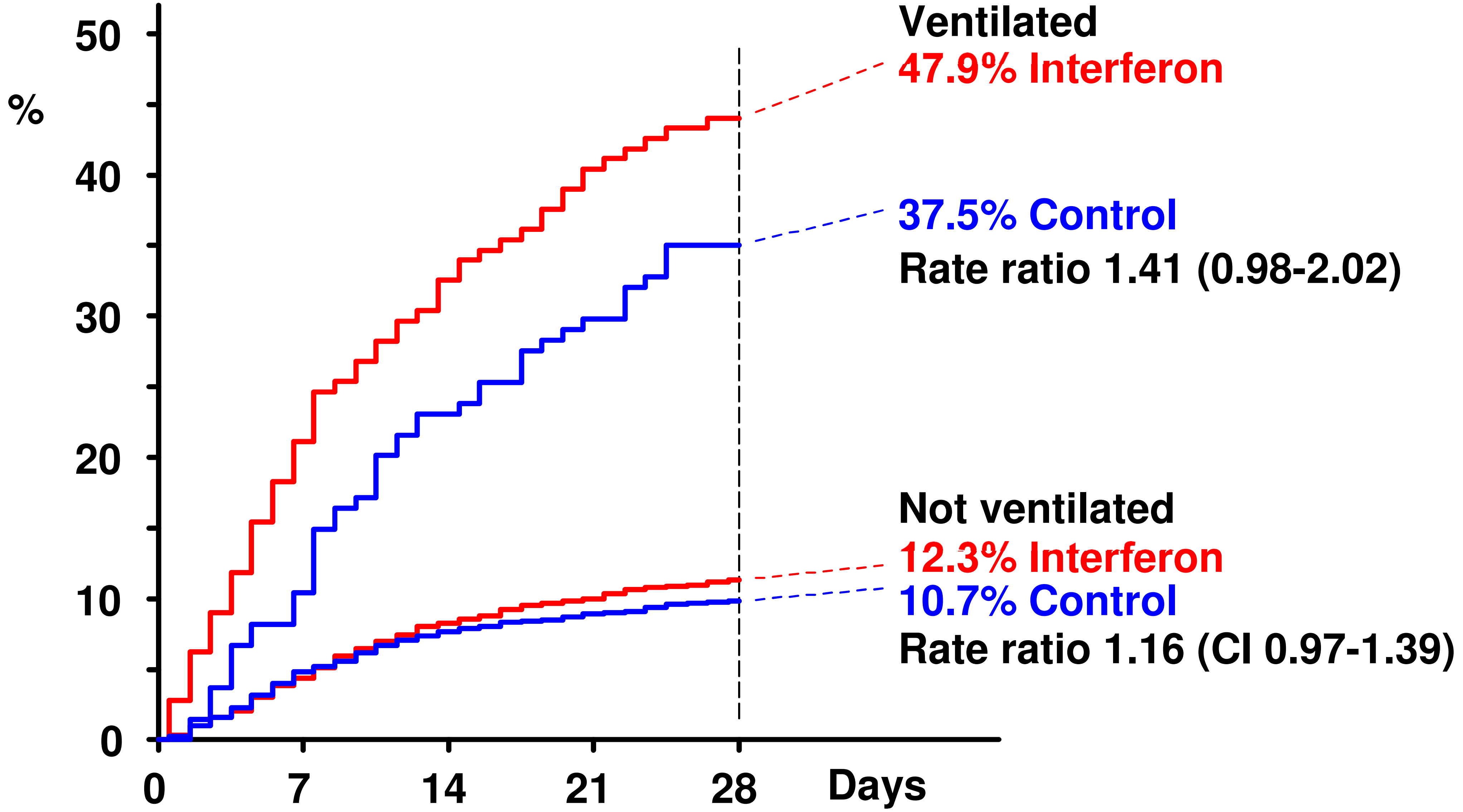

Weekly denominators (all not yet dead/lost), and numbers dying in hospital

Interferon	144	30	111	16	94	11	83	5	78	7	Ventilated
Control	136	14	120	17	103	9	94	7	87	4	
Interferon	2000	86	1888	78	1810	34	1776	26	1750	23	Not ventilated
Control	2011	96	1895	56	1836	26	1810	17	1793	20	

Figure S3A-S3D. Death rate ratios, stratified by age and respiratory support at entry, for (A) remdesivir, (B) hydroxychloroquine, (C) lopinavir, (D) interferon, each vs its control

Analyses in subgroups of age are stratified by ventilation, and vice-versa, so each total is stratified for both factors.

	Deaths reported / Patients in ITT analyses (%)		Active-group deaths: log-rank statistics		Death rate ratio RR, & 95% CI
	Active	Control	O-E	Variance	Active : Control
**(A) Remdesivir**					
**Age at entry**					
<50	105/1310 (8.0)	95/1326 (7.2)	8.0	49.6	1.18 [0.89-1.55]
50-69	275/1920 (14.3)	287/1908 (15.0)	-12.3	139.2	0.92 [0.78-1.08]
70+	222/916 (24.2)	261/895 (29.2)	-23.4	118.9	0.82 [0.69-0.98]
**Respiratory support at entry**					
Not ventilated	451/3787 (11.9)	509/3782 (13.5)	-35.9	237.9	0.86 [0.76-0.98]
Ventilated	151/359 (42.1)	134/347 (38.6)	8.3	69.8	1.13 [0.89-1.42]
**Total**	**602/4146 (14.5)**	**643/4129 (15.6)**	**-27.6**	**307.7**	**0.91 [0.82-1.02]**
**Heterogeneity around total χ^2_3: 8.4**					**p = 0.12**
**(B) Hydroxychloroquine**					
**Age at entry**					
<50	19/336 (5.7)	21/316 (6.6)	0.0	9.6	1.00 [0.53-1.89]
50-69	55/410 (13.4)	33/394 (8.4)	8.8	21.6	1.50 [0.99-2.29]
70+	30/202 (14.9)	35/190 (18.4)	-3.7	16.0	0.80 [0.49-1.30]
**Respiratory support at entry**					
Not ventilated	69/864 (8.0)	61/818 (7.5)	1.8	32.3	1.06 [0.75-1.49]
Ventilated	35/84 (41.7)	28/82 (34.1)	3.4	15.0	1.25 [0.76-2.08]
**Total**	**104/948 (11.0)**	**89/900 (9.9)**	**5.2**	**47.3**	**1.12 [0.84-1.48]**
**Heterogeneity around total χ^2_3: 4.2**					**p = 0.45**
**(C) Lopinavir**					
**Age at entry**					
<50	22/513 (4.3)	30/504 (6.0)	-3.0	12.9	0.79 [0.46-1.37]
50-69	68/602 (11.3)	59/594 (9.9)	1.5	31.4	1.05 [0.74-1.49]
70+	61/289 (21.1)	64/270 (23.7)	-3.2	30.3	0.90 [0.63-1.29]
**Respiratory support at entry**					
Not ventilated	117/1292 (9.1)	117/1253 (9.3)	-5.3	57.8	0.91 [0.71-1.18]
Ventilated	34/112 (30.4)	36/115 (31.3)	0.6	16.7	1.04 [0.64-1.68]
**Total**	**151/1404 (10.8)**	**153/1368 (11.2)**	**-4.7**	**74.5**	**0.94 [0.75-1.18]**
**Heterogeneity around total χ^2_3: 1.0**					**p = 0.59**
**(D) Interferon**					
**Age at entry**					
<50	60/759 (7.9)	43/731 (5.9)	8.0	25.6	1.37 [0.93-2.01]
50-69	165/970 (17.0)	131/1017 (12.9)	23.3	73.3	1.37 [1.09-1.73]
70+	91/415 (21.9)	92/399 (23.1)	-4.2	45.1	0.91 [0.68-1.22]
**Respiratory support at entry**					
Not ventilated	247/2000 (12.3)	215/2011 (10.7)	17.1	114.6	1.16 [0.97-1.39]
Ventilated	69/144 (47.9)	51/136 (37.5)	10.0	29.3	1.41 [0.98-2.02]
**Total**	**316/2144 (14.7)**	**266/2147 (12.4)**	**27.1**	**143.9**	**1.21 [1.03-1.42]**
**Heterogeneity around total χ^2_3: 6.0**					**p = 0.02**

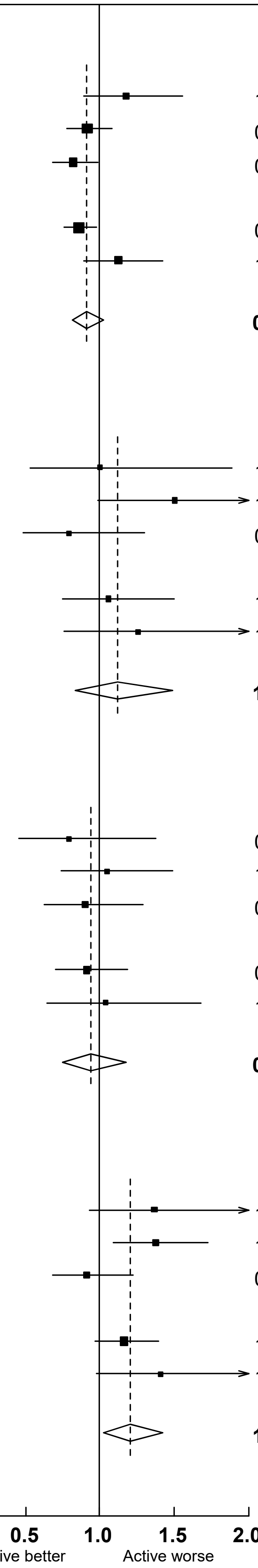

Figure S4A. In-hospital mortality rate ratios, stratified by age and respiratory support at entry, remdesivir vs its control, by entry characteristics and by steroid use at any time*

	Deaths reported / Patients in ITT analyses (%)		Remdesivir deaths: log-rank statistics		Death rate ratio RR, & 95% CI
	Remdesivir	Control	O-E	Variance	Remdesivir : Control
**Sex**					
Male	398/2601 (15.3)	450/2639 (17.1)	-28.9	209.4	0.87 [0.76-1.00]
Female	204/1545 (13.2)	193/1490 (13.0)	2.9	98.0	1.03 [0.84-1.26]
**Age at entry**					
<50	105/1310 (8.0)	95/1326 (7.2)	8.0	49.6	1.18 [0.89-1.55]
50-69	275/1920 (14.3)	287/1908 (15.0)	-12.3	139.2	0.92 [0.78-1.08]
70+	222/916 (24.2)	261/895 (29.2)	-23.4	118.9	0.82 [0.69-0.98]
**Days from hospital admission to randomisation**					
0	114/888 (12.8)	120/892 (13.5)	-6.6	57.1	0.89 [0.69-1.16]
1	164/1462 (11.2)	201/1459 (13.8)	-22.1	90.3	0.78 [0.64-0.96]
2+	324/1796 (18.0)	322/1778 (18.1)	0.5	158.7	1.00 [0.86-1.17]
**Respiratory support at entry**					
No O_2	25/869 (2.9)	33/861 (3.8)	-3.9	14.4	0.76 [0.46-1.28]
Low-flow or high-flow O_2	426/2918 (14.6)	476/2921 (16.3)	-32.0	223.5	0.87 [0.76-0.99]
Ventilation	151/359 (42.1)	134/347 (38.6)	8.3	69.8	1.13 [0.89-1.42]
**Bilateral lung lesions**					
Not imaged at entry	70/399 (17.5)	59/417 (14.1)	4.2	31.8	1.14 [0.81-1.61]
Yes	502/3326 (15.1)	545/3341 (16.3)	-21.6	258.4	0.92 [0.81-1.04]
No	30/421 (7.1)	39/371 (10.5)	-8.6	16.8	0.60 [0.37-0.97]
**Current smoking**					
Yes	30/247 (12.1)	40/233 (17.2)	-7.4	16.7	0.64 [0.40-1.04]
No	572/3899 (14.7)	603/3896 (15.5)	-19.3	290.4	0.94 [0.83-1.05]
**Diabetes**					
Yes	194/1129 (17.2)	210/1120 (18.8)	-14.5	99.4	0.86 [0.71-1.05]
No	408/3017 (13.5)	433/3009 (14.4)	-14.6	207.9	0.93 [0.81-1.07]
**Heart disease**					
Yes	163/929 (17.5)	215/935 (23.0)	-27.9	92.5	0.74 [0.60-0.91]
No	439/3217 (13.6)	428/3194 (13.4)	-0.5	214.3	1.00 [0.87-1.14]
**Chronic liver disease**					
Yes	14/57 (24.6)	15/72 (20.8)	0.9	6.5	1.14 [0.53-2.47]
No	588/4089 (14.4)	628/4057 (15.5)	-28.7	300.4	0.91 [0.81-1.02]
**Chronic lung disease**					
Yes	62/284 (21.8)	65/281 (23.1)	-2.7	31.0	0.92 [0.64-1.30]
No	540/3862 (14.0)	578/3848 (15.0)	-25.1	276.4	0.91 [0.81-1.03]
**Asthma**					
Yes	31/247 (12.6)	35/242 (14.5)	-1.0	15.9	0.94 [0.57-1.53]
No	571/3899 (14.6)	608/3887 (15.6)	-27.1	291.2	0.91 [0.81-1.02]
**Corticosteroids at entry or later (ignoring any biases*)**					
Yes	490/2782 (17.6)	539/2820 (19.1)	-23.0	254.4	0.91 [0.81-1.03]
No	112/1363 (8.2)	104/1309 (7.9)	-3.3	52.4	0.94 [0.72-1.23]
**Geographic location‡**					
Europe or Canada	196/1649 (11.9)	229/1594 (14.4)	-22.0	105.2	0.81 [0.67-0.98]
Latin America	116/558 (20.8)	145/593 (24.5)	-8.6	63.0	0.87 [0.68-1.12]
Asia and Africa	290/1939 (15.0)	269/1942 (13.9)	1.7	135.8	1.01 [0.86-1.20]
**Total**	**602/4146 (14.5)**	**643/4129 (15.6)**	**-27.6**	**307.7**	**0.91 [0.82-1.02] p = 0.12**

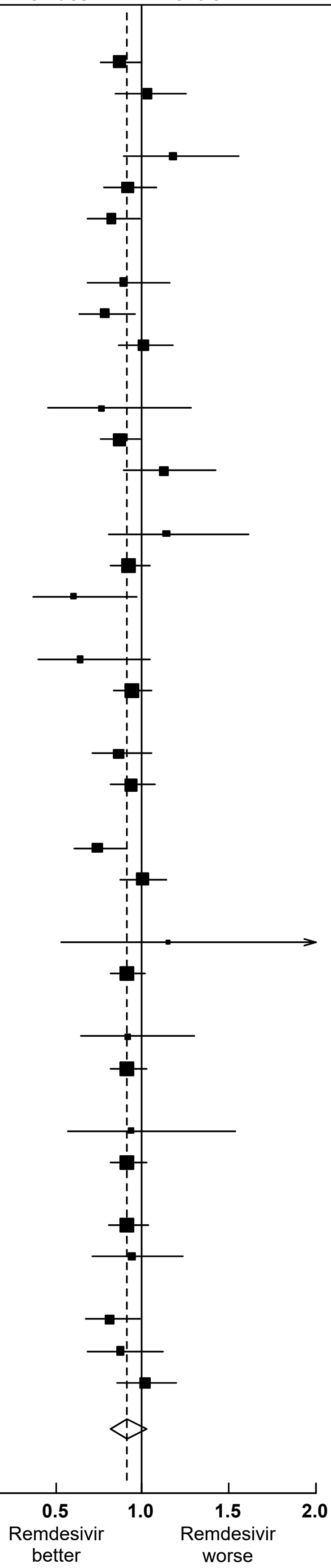

Heterogeneity test: $\chi^2_{18} = 30.4$; $p = 0.03$

* RRs in steroid subgroups may be somewhat biased by later steroid use.

‡ Wastefully fine stratification by country and, within country, by the 9 strata of age and ventilation yielded RR=0.94, 95% CI 0.84-1.06, with no good evidence of between-country RR heterogeneity.

Figure S4B. In-hospital mortality rate ratios, stratified by age and respiratory support at entry, hydroxychloroquine vs its control, by entry characteristics and by steroid use at any time*

	Deaths reported / Patients in ITT analyses (%)		Hydroxychlor. deaths: log-rank statistics		Death rate ratio RR, & 95% CI
	Hydroxychloroquine	Control	O-E	Variance	Hydroxychlor. : Control
**Sex**					
Male	80/574 (13.9)	54/532 (10.2)	10.9	32.7	1.40 [0.99-1.97]
Female	24/374 (6.4)	35/368 (9.5)	-5.8	14.5	0.67 [0.40-1.12]
**Age at entry**					
<50	19/336 (5.7)	21/316 (6.6)	0.0	9.6	1.00 [0.53-1.89]
50-69	55/410 (13.4)	33/394 (8.4)	8.8	21.6	1.50 [0.99-2.29]
70+	30/202 (14.9)	35/190 (18.4)	-3.7	16.0	0.80 [0.49-1.30]
**Days from hospital admission to randomisation**					
0	23/296 (7.8)	19/280 (6.8)	1.5	10.3	1.16 [0.63-2.14]
1	39/319 (12.2)	31/307 (10.1)	1.6	16.5	1.10 [0.68-1.79]
2+	42/333 (12.6)	39/313 (12.5)	0.5	19.8	1.02 [0.66-1.59]
**Respiratory support at entry**					
No O_2	8/346 (2.3)	6/338 (1.8)	0.9	3.5	1.29 [0.45-3.69]
Low-flow or high-flow O_2	61/518 (11.8)	55/480 (11.5)	0.9	28.8	1.03 [0.72-1.49]
Ventilation	35/84 (41.7)	28/82 (34.1)	3.4	15.0	1.25 [0.76-2.08]
**Bilateral lung lesions**					
Not imaged at entry	18/116 (15.5)	17/106 (16.0)	-0.3	8.5	0.96 [0.49-1.88]
Yes	82/714 (11.5)	72/670 (10.7)	3.4	37.4	1.09 [0.79-1.51]
No	4/118 (3.4)	0/124 (0.0)	2.0	1.0	7.57 [1.03-55.57]
**Current smoking**					
Yes	14/93 (15.1)	8/82 (9.8)	3.0	4.0	2.14 [0.80-5.71]
No	90/855 (10.5)	81/818 (9.9)	2.3	41.8	1.06 [0.78-1.43]
**Diabetes**					
Yes	36/201 (17.9)	21/205 (10.2)	7.9	13.5	1.79 [1.05-3.05]
No	68/747 (9.1)	68/695 (9.8)	-1.8	33.4	0.95 [0.68-1.33]
**Heart disease**					
Yes	25/194 (12.9)	21/194 (10.8)	1.7	11.1	1.16 [0.65-2.09]
No	79/754 (10.5)	68/706 (9.6)	2.8	35.8	1.08 [0.78-1.50]
**Chronic liver disease**					
Yes	2/15 (13.3)	3/14 (21.4)	-1.5	0.9	0.19 [0.02-1.46]
No	102/933 (10.9)	86/886 (9.7)	5.9	46.1	1.14 [0.85-1.52]
**Chronic lung disease**					
Yes	9/63 (14.3)	7/65 (10.8)	0.8	3.9	1.23 [0.45-3.32]
No	95/885 (10.7)	82/835 (9.8)	3.9	43.3	1.09 [0.81-1.47]
**Asthma**					
Yes	3/44 (6.8)	4/47 (8.5)	0.1	1.2	1.06 [0.18-6.33]
No	101/904 (11.2)	85/853 (10.0)	5.0	45.5	1.12 [0.83-1.49]
**Corticosteroids at entry or later (ignoring any biases*)**					
Yes	41/190 (21.6)	38/197 (19.3)	3.6	18.8	1.21 [0.77-1.91]
No	63/758 (8.3)	51/703 (7.3)	2.5	27.7	1.09 [0.75-1.59]
**Geographic location‡**					
Europe or Canada	24/287 (8.4)	18/266 (6.8)	2.7	10.4	1.30 [0.71-2.39]
Latin America	27/97 (27.8)	21/96 (21.9)	3.8	11.1	1.41 [0.78-2.54]
Asia and Africa	53/564 (9.4)	50/538 (9.3)	1.3	24.7	1.05 [0.71-1.56]
**Scheduled Hydroxychloroquine dose**					
Full dose	89/799 (11.1)	76/751 (10.1)	4.8	39.5	1.13 [0.83-1.54]
Half dose (Discovery)	15/149 (10.1)	13/149 (8.7)	0.9	6.9	1.14 [0.54-2.41]
**Total**	**104/948 (11.0)**	**89/900 (9.9)**	**5.2**	**47.3**	**1.12 [0.84-1.48]** p = 0.45

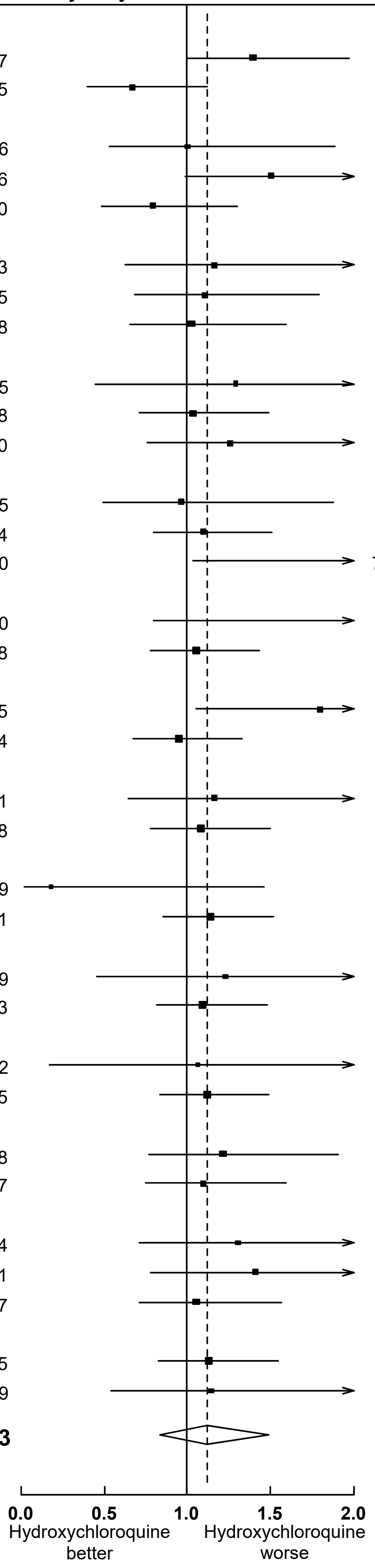

Heterogeneity test: χ^2_{19} = 23.2; p = 0.23

* RRs in steroid subgroups may be somewhat biased by later steroid use.

‡ Wastefully fine stratification by country and, within country, by the 9 strata of age and ventilation yielded RR=1.19, 95% CI 0.88-1.62, with no good evidence of between-country RR heterogeneity.

Figure S4C. In-hospital mortality rate ratios, stratified by age and respiratory support at entry, lopinavir vs its control, by entry characteristics and by steroid use at any time*

	Deaths reported / Patients in ITT analyses (%)		Lopinavir deaths: log-rank statistics		Death rate ratio RR, & 95% CI
	Lopinavir	Control	O-E	Variance	Lopinavir : Control
**Sex**					
Male	96/852 (11.3)	96/800 (12.0)	-3.4	46.8	0.93 [0.70-1.24]
Female	55/552 (10.0)	57/568 (10.0)	-1.2	27.3	0.96 [0.66-1.39]
**Age at entry**					
<50	22/513 (4.3)	30/504 (6.0)	-3.0	12.9	0.79 [0.46-1.37]
50-69	68/602 (11.3)	59/594 (9.9)	1.5	31.4	1.05 [0.74-1.49]
70+	61/289 (21.1)	64/270 (23.7)	-3.2	30.3	0.90 [0.63-1.29]
**Days from hospital admission to randomisation**					
0	34/422 (8.1)	34/401 (8.5)	-0.6	16.9	0.97 [0.60-1.56]
1	53/450 (11.8)	48/444 (10.8)	0.5	24.4	1.02 [0.69-1.52]
2+	64/532 (12.0)	71/523 (13.6)	-1.5	32.2	0.95 [0.67-1.35]
**Respiratory support at entry**					
No O_2	21/527 (4.0)	17/535 (3.2)	2.5	9.4	1.31 [0.69-2.48]
Low-flow or high-flow O_2	96/765 (12.5)	100/718 (13.9)	-7.8	48.3	0.85 [0.64-1.13]
Ventilation	34/112 (30.4)	36/115 (31.3)	0.6	16.7	1.04 [0.64-1.68]
**Bilateral lung lesions**					
Not imaged at entry	27/163 (16.6)	22/157 (14.0)	0.7	11.9	1.06 [0.60-1.87]
Yes	120/1048 (11.5)	121/1001 (12.1)	-3.4	59.0	0.94 [0.73-1.22]
No	4/193 (2.1)	10/210 (4.8)	-2.0	3.0	0.50 [0.16-1.57]
**Current smoking**					
Yes	13/137 (9.5)	12/121 (9.9)	-0.1	5.3	0.98 [0.42-2.29]
No	138/1267 (10.9)	141/1247 (11.3)	-4.9	68.6	0.93 [0.73-1.18]
**Diabetes**					
Yes	46/346 (13.3)	33/326 (10.1)	1.9	18.9	1.10 [0.70-1.73]
No	105/1058 (9.9)	120/1042 (11.5)	-7.0	54.8	0.88 [0.68-1.15]
**Heart disease**					
Yes	45/292 (15.4)	39/291 (13.4)	2.9	20.1	1.15 [0.75-1.79]
No	106/1112 (9.5)	114/1077 (10.6)	-7.5	54.1	0.87 [0.67-1.14]
**Chronic liver disease**					
Yes	2/16 (12.5)	4/23 (17.4)	-0.6	1.0	0.57 [0.08-3.98]
No	149/1388 (10.7)	149/1345 (11.1)	-4.2	73.0	0.94 [0.75-1.19]
**Chronic lung disease**					
Yes	15/94 (16.0)	12/86 (14.0)	1.0	6.3	1.18 [0.54-2.57]
No	136/1310 (10.4)	141/1282 (11.0)	-5.9	67.8	0.92 [0.72-1.16]
**Asthma**					
Yes	7/68 (10.3)	7/57 (12.3)	0.2	2.0	1.10 [0.27-4.41]
No	144/1336 (10.8)	146/1311 (11.1)	-4.3	71.2	0.94 [0.75-1.19]
**Corticosteroids at entry or later (ignoring any biases*)**					
Yes	77/367 (21.0)	87/387 (22.5)	-1.1	39.9	0.97 [0.71-1.33]
No	74/1036 (7.1)	66/981 (6.7)	-1.0	34.0	0.97 [0.69-1.36]
**Geographic location‡**					
Europe or Canada	45/348 (12.9)	42/353 (11.9)	1.7	21.0	1.09 [0.71-1.67]
Latin America	30/147 (20.4)	34/148 (23.0)	-0.2	15.3	0.99 [0.60-1.63]
Asia and Africa	76/909 (8.4)	77/867 (8.9)	-4.0	37.0	0.90 [0.65-1.24]
**Total**	**151/1404 (10.8)**	**153/1368 (11.2)**	**-4.7**	**74.5**	**0.94 [0.75-1.18]** p = 0.59

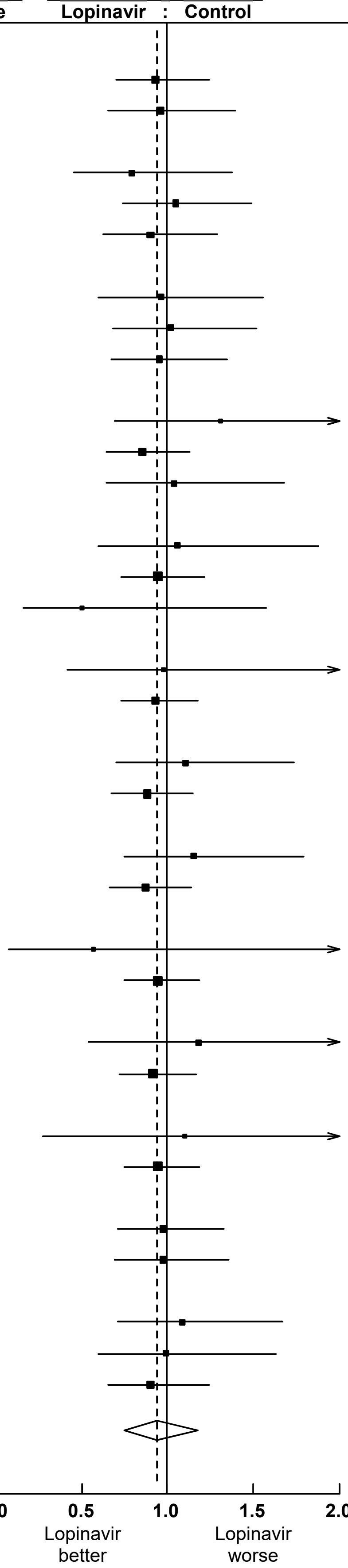

Heterogeneity test: χ^2_{18}= 6.9; p = 0.99

* RRs in steroid subgroups may be somewhat biased by later steroid use.

‡ Wastefully fine stratification by country and, within country, by the 9 strata of age and ventilation yielded RR=0.93, 95% CI 0.73-1.18, with no good evidence of between-country RR heterogeneity.

Figure S4D. In-hospital mortality rate ratios, stratified by age and respiratory support at entry, interferon vs its control, by entry characteristics and by steroid use at any time*

	Deaths reported / Patients in ITT analyses (%)		Interferon deaths: log-rank statistics		Death rate ratio RR, & 95% CI
	Interferon	Control	O-E	Variance	Interferon : Control
**Sex**					
Male	215/1342 (16.0)	177/1331 (13.3)	19.4	96.9	1.22 [1.00-1.49]
Female	101/802 (12.6)	89/816 (10.9)	7.3	46.5	1.17 [0.88-1.56]
**Age at entry**					
<50	60/759 (7.9)	43/731 (5.9)	8.0	25.6	1.37 [0.93-2.01]
50-69	165/970 (17.0)	131/1017 (12.9)	23.3	73.3	1.37 [1.09-1.73]
70+	91/415 (21.9)	92/399 (23.1)	-4.2	45.1	0.91 [0.68-1.22]
**Days from hospital admission to randomisation**					
0	87/707 (12.3)	85/702 (12.1)	3.7	41.6	1.09 [0.81-1.48]
1	90/700 (12.9)	89/699 (12.7)	-1.4	44.1	0.97 [0.72-1.30]
2+	139/737 (18.9)	92/746 (12.3)	25.4	56.2	1.57 [1.21-2.04]
**Respiratory support at entry**					
No O_2	12/503 (2.4)	16/508 (3.1)	-1.8	7.0	0.78 [0.37-1.63]
Low-flow or high-flow O_2	235/1497 (15.7)	199/1503 (13.2)	18.9	107.6	1.19 [0.99-1.44]
Ventilation	69/144 (47.9)	51/136 (37.5)	10.0	29.3	1.41 [0.98-2.02]
**Bilateral lung lesions**					
Not imaged at entry	29/159 (18.2)	26/160 (16.2)	1.8	13.4	1.14 [0.67-1.95]
Yes	283/1857 (15.2)	235/1865 (12.6)	24.4	128.0	1.21 [1.02-1.44]
No	4/128 (3.1)	5/122 (4.1)	-0.1	1.8	0.92 [0.21-3.98]
**Current smoking**					
Yes	18/142 (12.7)	22/147 (15.0)	-2.2	9.3	0.79 [0.41-1.50]
No	298/2002 (14.9)	244/2000 (12.2)	29.0	134.1	1.24 [1.05-1.47]
**Diabetes**					
Yes	98/517 (19.0)	71/563 (12.6)	18.0	41.0	1.55 [1.14-2.11]
No	218/1627 (13.4)	195/1584 (12.3)	7.9	101.7	1.08 [0.89-1.31]
**Heart disease**					
Yes	82/457 (17.9)	75/487 (15.4)	5.5	37.8	1.16 [0.84-1.59]
No	234/1687 (13.9)	191/1660 (11.5)	22.0	105.0	1.23 [1.02-1.49]
**Chronic liver disease**					
Yes	6/14 (42.9)	5/24 (20.8)	1.5	1.3	3.10 [0.56-17.04]
No	310/2130 (14.6)	261/2123 (12.3)	25.7	141.2	1.20 [1.02-1.42]
**Chronic lung disease**					
Yes	30/120 (25.0)	27/110 (24.5)	2.7	13.4	1.22 [0.72-2.09]
No	286/2024 (14.1)	239/2037 (11.7)	25.1	129.9	1.21 [1.02-1.44]
**Asthma**					
Yes	6/79 (7.6)	12/105 (11.4)	-1.5	3.9	0.68 [0.25-1.84]
No	310/2065 (15.0)	254/2042 (12.4)	27.9	139.4	1.22 [1.03-1.44]
**Corticosteroids at entry or later (ignoring any biases*)**					
Yes	249/1232 (20.2)	207/1286 (16.1)	26.3	112.3	1.26 [1.05-1.52]
No	67/912 (7.3)	59/861 (6.9)	2.0	30.9	1.07 [0.75-1.52]
**Geographic location‡**					
Europe or Canada	29/268 (10.8)	25/256 (9.8)	1.4	13.2	1.11 [0.65-1.91]
Latin America	132/493 (26.8)	101/495 (20.4)	17.3	57.1	1.35 [1.05-1.76]
Asia and Africa	155/1383 (11.2)	140/1396 (10.0)	9.4	72.6	1.14 [0.90-1.43]
**Control Type**					
Lopinavir	86/648 (13.3)	69/677 (10.2)	10.5	37.8	1.32 [0.96-1.82]
Standard care	230/1496 (15.4)	197/1470 (13.4)	18.4	105.1	1.19 [0.98-1.44]
**Scheduled Interferon regimen**					
Subcutaneous	181/1679 (10.8)	149/1683 (8.9)	15.1	81.2	1.20 [0.97-1.50]
Intravenous†	135/465 (29.0)	117/464 (25.2)	10.1	61.9	1.18 [0.92-1.51]
**Total**	**316/2144 (14.7)**	**266/2147 (12.4)**	**27.1**	**143.9**	**1.21 [1.03-1.42] p = 0.02**

Heterogeneity test: χ^2_{20} = 24.2; p = 0.23

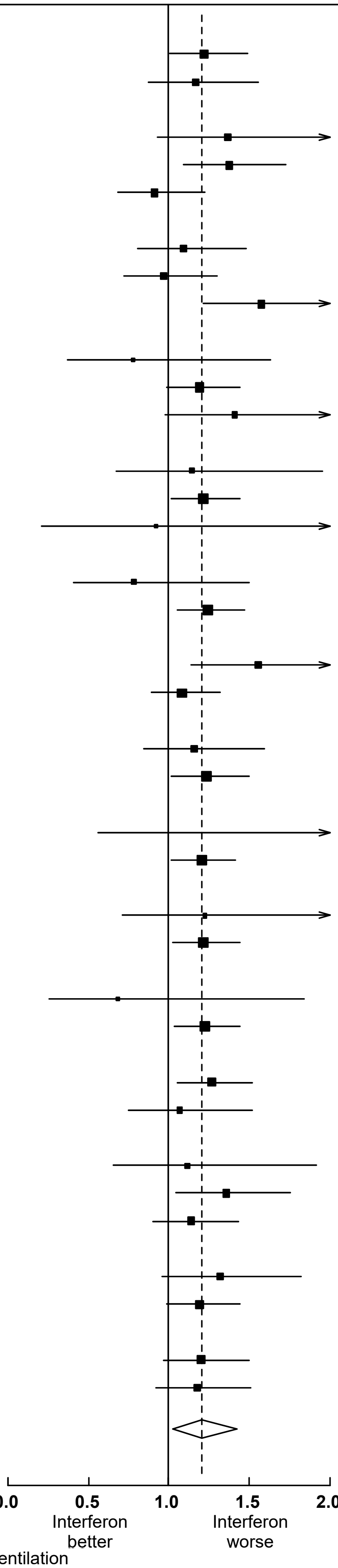

* RRs in steroid subgroups may be somewhat biased by later steroid use.

† Possible only if high-flow oxygen or ventilation was being given (and the country had been supplied with intravenous interferon)

‡ Wastefully fine stratification by country and, within country, by the 9 strata of age and ventilation yielded RR=1.25, 95% CI 1.06-1.49, with no good evidence of between-country RR heterogeneity.

Figure S5A-S5D. Rate ratios for initiation of ventilation among those not already ventilated at entry, stratified by age and respiratory support at entry, for (A) remdesivir, (B) hydroxy-chloroquine, (C) lopinavir, (D) interferon, each vs its control

Analyses in subgroups of age are stratified by oxygen use, and vice-versa, so each total is stratified for both factors.

	Events reported / Patients in ITT analyses (%)		Active-group events: log-rank statistics		Event rate ratio RR, & 95% CI
	Active	Control	O-E	Variance	Active : Control
**(A) Remdesivir**					
**Age at entry**					
<50	114/1228 (9.3)	143/1246 (11.5)	-11.0	55.9	0.82 [0.63-1.07]
50-69	258/1722 (15.0)	274/1727 (15.9)	-9.8	110.0	0.91 [0.76-1.10]
70+	163/837 (19.5)	176/809 (21.8)	-9.7	66.2	0.86 [0.68-1.10]
**Respiratory support at entry**					
No O_2	39/869 (4.5)	40/861 (4.6)	-0.4	18.6	0.98 [0.62-1.54]
Low-flow or high-flow O_2	496/2918 (17.0)	553/2921 (18.9)	-30.2	213.5	0.87 [0.76-0.99]
**Total**	**535/3787 (14.1)**	**593/3782 (15.7)**	**-30.6**	**232.1**	**0.88 [0.77-1.00] p = 0.04**
**Heterogeneity around total: χ^2_3 = 0.7; p = 0.87**					
**(B) Hydroxychloroquine**					
**Age at entry**					
<50	15/324 (4.6)	16/294 (5.4)	-1.3	7.1	0.83 [0.40-1.74]
50-69	38/366 (10.4)	31/359 (8.6)	2.1	15.1	1.15 [0.69-1.90]
70+	22/174 (12.6)	18/165 (10.9)	1.7	8.8	1.22 [0.63-2.36]
**Respiratory support at entry**					
No O_2	11/346 (3.2)	5/338 (1.5)	2.8	3.8	2.07 [0.76-5.63]
Low-flow or high-flow O_2	64/518 (12.4)	60/480 (12.5)	-0.3	27.1	0.99 [0.68-1.44]
**Total**	**75/864 (8.7)**	**65/818 (7.9)**	**2.5**	**30.9**	**1.08 [0.76-1.54] p = 0.65**
**Heterogeneity around total: χ^2_3 = 2.5; p = 0.48**					
**(C) Lopinavir**					
**Age at entry**					
<50	20/486 (4.1)	32/469 (6.8)	-5.8	11.8	0.61 [0.35-1.09]
50-69	70/539 (13.0)	57/549 (10.4)	5.9	27.5	1.24 [0.85-1.80]
70+	34/267 (12.7)	33/235 (14.0)	-2.3	14.2	0.85 [0.50-1.43]
**Respiratory support at entry**					
No O_2	19/527 (3.6)	20/535 (3.7)	0.2	9.2	1.03 [0.54-1.96]
Low-flow or high-flow O_2	105/765 (13.7)	102/718 (14.2)	-2.4	44.3	0.95 [0.70-1.27]
**Total**	**124/1292 (9.6)**	**122/1253 (9.7)**	**-2.2**	**53.5**	**0.96 [0.73-1.25] p = 0.76**
**Heterogeneity around total: χ^2_3 = 4.4; p = 0.22**					
**(D) Interferon**					
**Age at entry**					
<50	49/727 (6.7)	63/705 (8.9)	-8.1	25.1	0.73 [0.49-1.07]
50-69	139/893 (15.6)	123/938 (13.1)	11.3	55.0	1.23 [0.94-1.60]
70+	68/380 (17.9)	59/368 (16.0)	3.3	25.5	1.14 [0.77-1.68]
**Respiratory support at entry**					
No O_2	14/503 (2.8)	16/508 (3.1)	-0.8	7.2	0.89 [0.43-1.84]
Low-flow or high-flow O_2	242/1497 (16.2)	229/1503 (15.2)	7.4	98.4	1.08 [0.88-1.31]
**Total**	**256/2000 (12.8)**	**245/2011 (12.2)**	**6.5**	**105.7**	**1.06 [0.88-1.29] p = 0.53**
**Heterogeneity around total: χ^2_3 = 5.2; p = 0.16**					

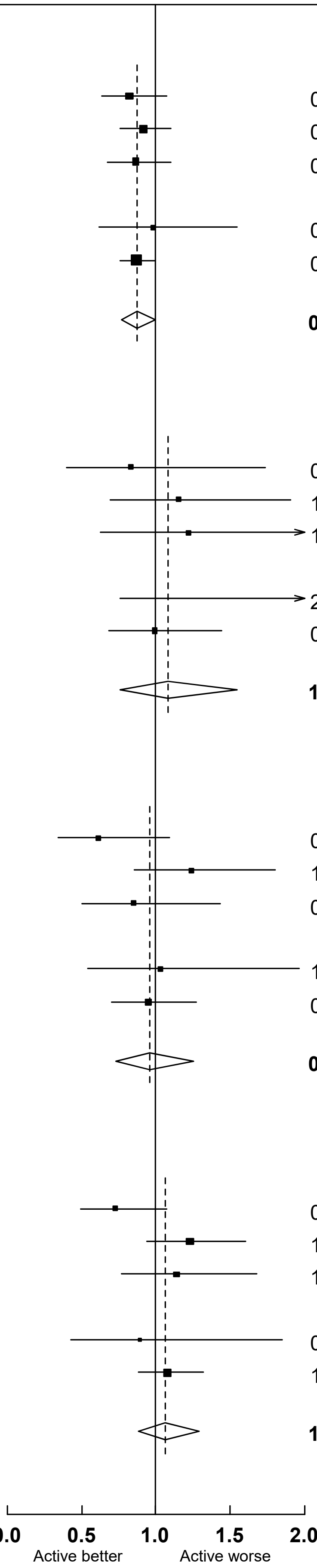

Figure S6A. Rate ratios for initiation of ventilation among those not already ventilated at entry, stratified by age and respiratory support at entry, remdesivir vs its control, by entry characteristics and by steroid use at any time*

	Events reported / Patients in ITT analyses (%)		Remdesivir events: log-rank statistics		Event rate ratio RR, & 95% CI
	Remdesivir	Control	O-E	Variance	Remdesivir : Control
**Sex**					
Male	372/2352 (15.8)	420/2408 (17.4)	-19.3	159.0	0.89 [0.76-1.03]
Female	163/1435 (11.4)	173/1374 (12.6)	-9.4	71.9	0.88 [0.70-1.11]
**Age at entry**					
<50	114/1228 (9.3)	143/1246 (11.5)	-11.0	55.9	0.82 [0.63-1.07]
50-69	258/1722 (15.0)	274/1727 (15.9)	-9.8	110.0	0.91 [0.76-1.10]
70+	163/837 (19.5)	176/809 (21.8)	-9.7	66.2	0.86 [0.68-1.10]
**Days from hospital admission to randomisation**					
0	114/833 (13.7)	121/863 (14.0)	-1.5	48.9	0.97 [0.73-1.28]
1	180/1373 (13.1)	238/1371 (17.4)	-28.5	85.8	0.72 [0.58-0.89]
2+	241/1581 (15.2)	234/1548 (15.1)	-0.5	97.4	0.99 [0.82-1.21]
**Respiratory support at entry**					
No O_2	39/869 (4.5)	40/861 (4.6)	-0.4	18.6	0.98 [0.62-1.54]
Low-flow or high-flow O_2	496/2918 (17.0)	553/2921 (18.9)	-30.2	213.5	0.87 [0.76-0.99]
**Bilateral lung lesions**					
Not imaged at entry	62/316 (19.6)	60/347 (17.3)	0.6	23.9	1.03 [0.69-1.53]
Yes	448/3060 (14.6)	498/3073 (16.2)	-24.6	195.0	0.88 [0.77-1.01]
No	25/411 (6.1)	35/362 (9.7)	-7.0	12.8	0.58 [0.33-1.00]
**Current smoking**					
Yes	32/221 (14.5)	38/211 (18.0)	-5.2	14.2	0.69 [0.41-1.17]
No	503/3566 (14.1)	555/3571 (15.5)	-25.5	217.8	0.89 [0.78-1.02]
**Diabetes**					
Yes	158/1011 (15.6)	197/1018 (19.4)	-21.0	71.3	0.75 [0.59-0.94]
No	377/2776 (13.6)	396/2764 (14.3)	-9.6	160.7	0.94 [0.81-1.10]
**Heart disease**					
Yes	130/846 (15.4)	155/830 (18.7)	-15.1	57.7	0.77 [0.59-1.00]
No	405/2941 (13.8)	438/2952 (14.8)	-15.5	174.4	0.91 [0.79-1.06]
**Chronic liver disease**					
Yes	11/50 (22.0)	12/64 (18.8)	-0.3	4.0	0.94 [0.35-2.49]
No	524/3737 (14.0)	581/3718 (15.6)	-31.0	227.3	0.87 [0.77-0.99]
**Chronic lung disease**					
Yes	53/253 (20.9)	46/252 (18.3)	3.4	19.8	1.19 [0.77-1.85]
No	482/3534 (13.6)	547/3530 (15.5)	-34.5	212.2	0.85 [0.74-0.97]
**Asthma**					
Yes	33/230 (14.3)	40/229 (17.5)	-1.7	14.7	0.89 [0.54-1.49]
No	502/3557 (14.1)	553/3553 (15.6)	-28.8	217.3	0.88 [0.77-1.00]
**Corticosteroids at entry or later (ignoring any biases*)**					
Yes	448/2512 (17.8)	517/2530 (20.4)	-33.1	192.8	0.84 [0.73-0.97]
No	87/1274 (6.8)	76/1252 (6.1)	4.1	36.4	1.12 [0.81-1.55]
**Geographic location**					
Europe or Canada	257/1488 (17.3)	311/1436 (21.7)	-30.7	111.7	0.76 [0.63-0.91]
Latin America	83/514 (16.1)	99/522 (19.0)	-7.4	37.0	0.82 [0.59-1.13]
Asia and Africa	195/1785 (10.9)	183/1824 (10.0)	6.2	81.5	1.08 [0.87-1.34]
**Total**	**535/3787 (14.1)**	**593/3782 (15.7)**	**-30.6**	**232.1**	**0.88 [0.77-1.00]** **p = 0.04**

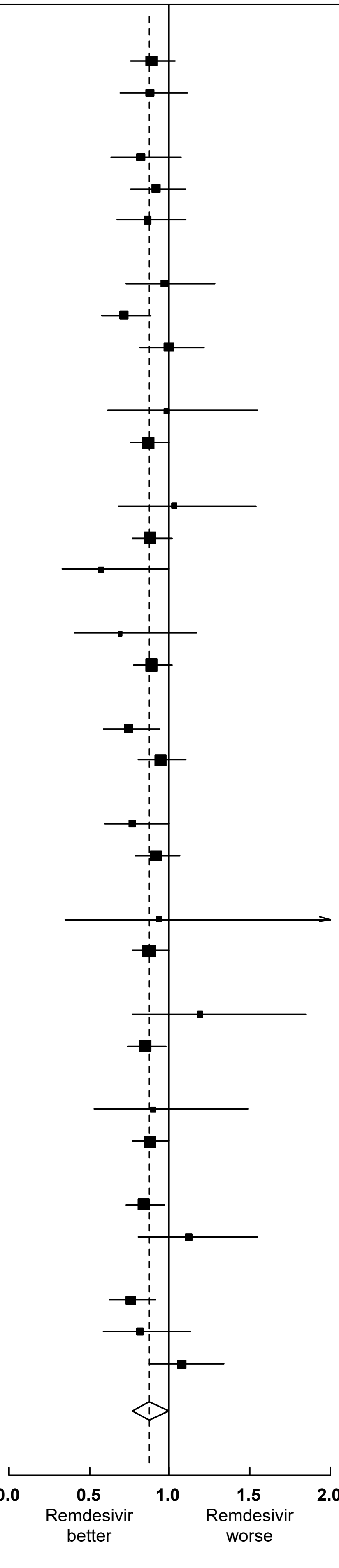

Heterogeneity test: χ^2_{17} = 24.3; p = 0.11

* RRs in steroid subgroups may be somewhat biased by later steroid use.

Figure S6B. Rate ratios for initiation of ventilation in those not already ventilated at entry, hydroxychloroquine vs its control, by entry characteristics and by steroid use at any time*

	Events reported / Patients in ITT analyses (%): Hydroxychloroquine	Events reported / Patients in ITT analyses (%): Control	Hydroxychlor. events: log-rank statistics: O-E	Hydroxychlor. events: log-rank statistics: Variance	Event rate ratio RR, & 95% CI Hydroxychlor. : Control
**Sex**					
Male	49/515 (9.5)	38/473 (8.0)	3.3	19.0	1.19 [0.76-1.86]
Female	26/349 (7.4)	27/345 (7.8)	-1.0	11.9	0.92 [0.52-1.62]
**Age at entry**					
<50	15/324 (4.6)	16/294 (5.4)	-1.3	7.1	0.83 [0.40-1.74]
50-69	38/366 (10.4)	31/359 (8.6)	2.1	15.1	1.15 [0.69-1.90]
70+	22/174 (12.6)	18/165 (10.9)	1.7	8.8	1.22 [0.63-2.36]
**Days from hospital admission to randomisation**					
0	18/285 (6.3)	10/270 (3.7)	3.6	6.5	1.75 [0.81-3.78]
1	29/284 (10.2)	33/276 (12.0)	-1.7	13.2	0.88 [0.51-1.51]
2+	28/295 (9.5)	22/272 (8.1)	2.0	10.7	1.21 [0.66-2.20]
**Respiratory support at entry**					
No O_2	11/346 (3.2)	5/338 (1.5)	2.8	3.8	2.07 [0.76-5.63]
Low-flow or high-flow O_2	64/518 (12.4)	60/480 (12.5)	-0.3	27.1	0.99 [0.68-1.44]
**Bilateral lung lesions**					
Not imaged at entry	21/87 (24.1)	15/76 (19.7)	1.4	6.7	1.23 [0.57-2.62]
Yes	52/662 (7.9)	48/622 (7.7)	0.4	22.6	1.02 [0.67-1.54]
No	2/115 (1.7)	2/120 (1.7)	0.0	0.9	0.98 [0.13-7.57]
**Current smoking**					
Yes	4/83 (4.8)	5/72 (6.9)	-0.1	1.8	0.94 [0.22-3.97]
No	71/781 (9.1)	60/746 (8.0)	3.4	28.9	1.12 [0.78-1.62]
**Diabetes**					
Yes	14/174 (8.0)	16/180 (8.9)	-1.3	6.7	0.82 [0.39-1.76]
No	61/690 (8.8)	49/638 (7.7)	4.5	24.1	1.20 [0.81-1.79]
**Heart disease**					
Yes	9/170 (5.3)	16/167 (9.6)	-3.9	5.7	0.50 [0.22-1.13]
No	66/694 (9.5)	49/651 (7.5)	6.5	25.1	1.30 [0.88-1.92]
**Chronic liver disease**					
Yes	0/12 (0.0)	1/13 (7.7)	-0.5	0.2	
No	75/852 (8.8)	64/805 (8.0)	2.9	30.7	1.10 [0.77-1.57]
**Chronic lung disease**					
Yes	4/54 (7.4)	4/56 (7.1)	0.1	1.8	1.05 [0.24-4.54]
No	71/810 (8.8)	61/762 (8.0)	2.3	29.1	1.08 [0.75-1.56]
**Asthma**					
Yes	5/44 (11.4)	2/41 (4.9)	0.9	1.5	1.84 [0.37-9.17]
No	70/820 (8.5)	63/777 (8.1)	1.4	29.4	1.05 [0.73-1.51]
**Corticosteroids at entry or later (ignoring any biases*)**					
Yes	20/155 (12.9)	33/162 (20.4)	-5.7	10.8	0.59 [0.32-1.07]
No	55/709 (7.8)	32/656 (4.9)	9.0	19.7	1.58 [1.01-2.46]
**Geographic location**					
Europe or Canada	32/245 (13.1)	31/226 (13.7)	-0.4	13.1	0.97 [0.56-1.66]
Latin America	14/88 (15.9)	10/85 (11.8)	1.1	5.0	1.25 [0.52-3.00]
Asia and Africa	29/531 (5.5)	24/507 (4.7)	1.2	12.2	1.10 [0.63-1.93]
**Scheduled Hydroxychloroquine dose**					
Full dose	55/753 (7.3)	43/708 (6.1)	4.0	22.2	1.20 [0.79-1.82]
Half dose (Discovery)	20/111 (18.0)	22/110 (20.0)	-1.4	8.5	0.85 [0.43-1.66]
**Total**	**75/864 (8.7)**	**65/818 (7.9)**	**2.5**	**30.9**	**1.08 [0.76-1.54]** p = 0.65

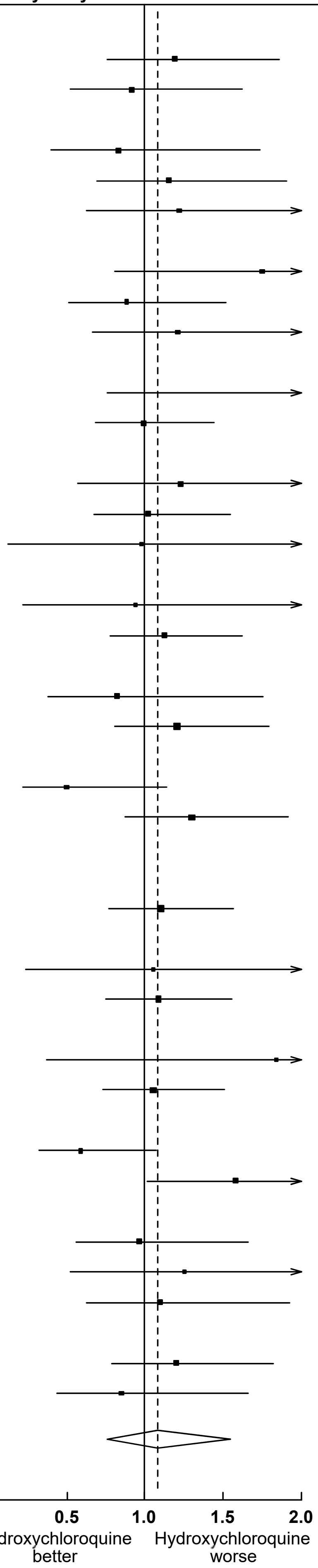

Heterogeneity test: χ^2_{18} = 19.4; p = 0.37

* RRs in steroid subgroups may be somewhat biased by later steroid use.

Figure S6C. Rate ratios for initiation of ventilation among those not already ventilated at entry, stratified by age and respiratory support at entry, lopinavir vs its control, by entry characteristics and by steroid use at any time*

	Events reported / Patients in ITT analyses (%)		Lopinavir events: log-rank statistics		Event rate ratio RR, & 95% CI
	Lopinavir	Control	O-E	Variance	Lopinavir : Control
**Sex**					
Male	89/778 (11.4)	81/720 (11.2)	0.0	35.9	1.00 [0.72-1.39]
Female	35/514 (6.8)	41/533 (7.7)	-2.8	17.2	0.85 [0.53-1.37]
**Age at entry**					
<50	20/486 (4.1)	32/469 (6.8)	-5.8	11.8	0.61 [0.35-1.09]
50-69	70/539 (13.0)	57/549 (10.4)	5.9	27.5	1.24 [0.85-1.80]
70+	34/267 (12.7)	33/235 (14.0)	-2.3	14.2	0.85 [0.50-1.43]
**Days from hospital admission to randomisation**					
0	36/409 (8.8)	22/389 (5.7)	6.6	12.9	1.67 [0.97-2.88]
1	40/416 (9.6)	46/405 (11.4)	-5.0	18.5	0.76 [0.48-1.20]
2+	48/467 (10.3)	54/459 (11.8)	-2.8	21.8	0.88 [0.58-1.34]
**Respiratory support at entry**					
No O_2	19/527 (3.6)	20/535 (3.7)	0.2	9.2	1.03 [0.54-1.96]
Low-flow or high-flow O_2	105/765 (13.7)	102/718 (14.2)	-2.4	44.3	0.95 [0.70-1.27]
**Bilateral lung lesions**					
Not imaged at entry	23/128 (18.0)	24/127 (18.9)	-1.5	9.3	0.85 [0.45-1.62]
Yes	96/972 (9.9)	91/922 (9.9)	0.1	41.1	1.00 [0.74-1.36]
No	5/192 (2.6)	7/204 (3.4)	-1.0	2.8	0.69 [0.21-2.24]
**Current smoking**					
Yes	10/127 (7.9)	9/109 (8.3)	-0.2	4.2	0.95 [0.36-2.46]
No	114/1165 (9.8)	113/1144 (9.9)	-1.9	49.2	0.96 [0.73-1.27]
**Diabetes**					
Yes	40/316 (12.7)	31/301 (10.3)	2.1	15.1	1.15 [0.69-1.90]
No	84/976 (8.6)	91/952 (9.6)	-4.3	38.2	0.89 [0.65-1.23]
**Heart disease**					
Yes	32/265 (12.1)	26/255 (10.2)	1.1	12.5	1.09 [0.63-1.90]
No	92/1027 (9.0)	96/998 (9.6)	-3.3	40.9	0.92 [0.68-1.25]
**Chronic liver disease**					
Yes	2/15 (13.3)	2/21 (9.5)	1.0	0.7	4.28 [0.39-47.24]
No	122/1277 (9.6)	120/1232 (9.7)	-2.9	52.7	0.95 [0.72-1.24]
**Chronic lung disease**					
Yes	10/88 (11.4)	7/74 (9.5)	0.7	3.6	1.22 [0.43-3.44]
No	114/1204 (9.5)	115/1179 (9.8)	-2.7	49.7	0.95 [0.72-1.25]
**Asthma**					
Yes	6/67 (9.0)	4/47 (8.5)	0.1	2.0	1.04 [0.26-4.13]
No	118/1225 (9.6)	118/1206 (9.8)	-1.8	51.4	0.96 [0.73-1.27]
**Corticosteroids at entry or later (ignoring any biases*)**					
Yes	64/324 (19.8)	83/329 (25.2)	-8.8	27.9	0.73 [0.50-1.06]
No	60/967 (6.2)	39/924 (4.2)	8.4	22.6	1.45 [0.96-2.19]
**Geographic location**					
Europe or Canada	48/291 (16.5)	51/296 (17.2)	-0.8	19.6	0.96 [0.62-1.50]
Latin America	18/144 (12.5)	24/137 (17.5)	-3.2	8.7	0.69 [0.36-1.34]
Asia and Africa	58/857 (6.8)	47/820 (5.7)	3.2	23.9	1.14 [0.77-1.71]
**Total**	**124/1292 (9.6)**	**122/1253 (9.7)**	**-2.2**	**53.5**	**0.96 [0.73-1.25]** p = 0.76

Heterogeneity test: χ^2_{17} = 20.5; p = 0.25

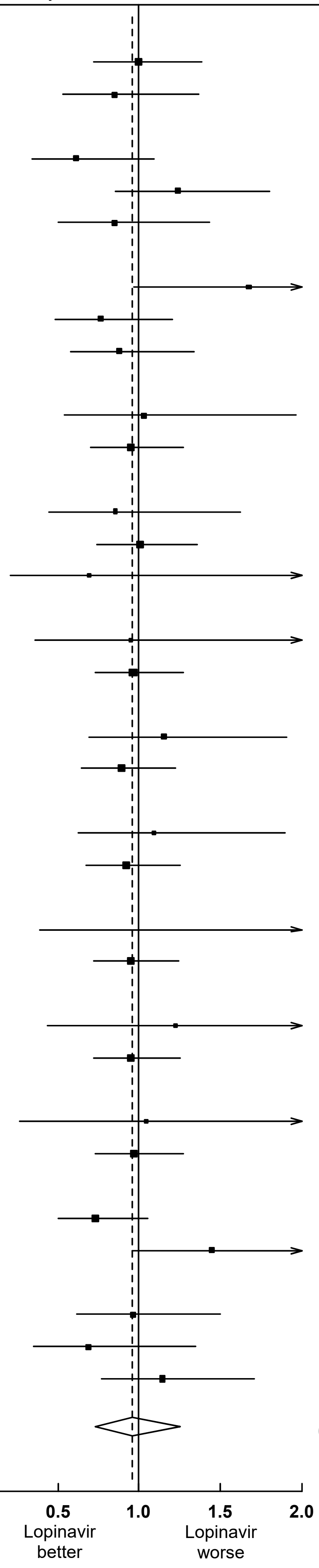

* RRs in steroid subgroups may be somewhat biased by later steroid use.

Figure S6D. Rate ratios for initiation of ventilation among those not already ventilated at entry, stratified by age and respiratory support at entry, interferon vs its control, by entry characteristics and by steroid use at any time*

	Events reported / Patients in ITT analyses (%)		Interferon events: log-rank statistics		Event rate ratio RR, & 95% CI
	Interferon	Control	O-E	Variance	Interferon : Control
**Sex**					
Male	176/1248 (14.1)	171/1244 (13.7)	2.9	71.5	1.04 [0.83-1.31]
Female	80/752 (10.6)	74/767 (9.6)	3.7	33.7	1.12 [0.80-1.56]
**Age at entry**					
<50	49/727 (6.7)	63/705 (8.9)	-8.1	25.1	0.73 [0.49-1.07]
50-69	139/893 (15.6)	123/938 (13.1)	11.3	55.0	1.23 [0.94-1.60]
70+	68/380 (17.9)	59/368 (16.0)	3.3	25.5	1.14 [0.77-1.68]
**Days from hospital admission to randomisation**					
0	66/688 (9.6)	74/678 (10.9)	-3.8	30.1	0.88 [0.62-1.26]
1	101/658 (15.3)	106/662 (16.0)	-2.5	41.9	0.94 [0.70-1.28]
2+	89/654 (13.6)	65/671 (9.7)	12.5	33.3	1.45 [1.04-2.04]
**Respiratory support at entry**					
No O_2	14/503 (2.8)	16/508 (3.1)	-0.8	7.2	0.89 [0.43-1.84]
Low-flow or high-flow O_2	242/1497 (16.2)	229/1503 (15.2)	7.4	98.4	1.08 [0.88-1.31]
**Bilateral lung lesions**					
Not imaged at entry	25/131 (19.1)	23/129 (17.8)	1.3	9.1	1.15 [0.60-2.21]
Yes	229/1743 (13.1)	219/1762 (12.4)	5.8	94.7	1.06 [0.87-1.30]
No	2/126 (1.6)	3/120 (2.5)	0.0	1.1	0.96 [0.15-6.18]
**Current smoking**					
Yes	18/133 (13.5)	19/136 (14.0)	-1.9	6.7	0.76 [0.35-1.61]
No	238/1867 (12.7)	226/1875 (12.1)	7.6	98.3	1.08 [0.89-1.32]
**Diabetes**					
Yes	74/482 (15.4)	69/519 (13.3)	4.6	29.6	1.17 [0.81-1.67]
No	182/1518 (12.0)	176/1492 (11.8)	2.3	75.8	1.03 [0.82-1.29]
**Heart disease**					
Yes	68/434 (15.7)	54/448 (12.1)	5.8	25.4	1.25 [0.85-1.85]
No	188/1566 (12.0)	191/1563 (12.2)	0.2	79.7	1.00 [0.80-1.25]
**Chronic liver disease**					
Yes	3/12 (25.0)	5/23 (21.7)	0.3	1.3	1.25 [0.23-6.98]
No	253/1988 (12.7)	240/1988 (12.1)	6.9	104.1	1.07 [0.88-1.30]
**Chronic lung disease**					
Yes	24/108 (22.2)	17/97 (17.5)	2.7	8.0	1.40 [0.70-2.79]
No	232/1892 (12.3)	228/1914 (11.9)	3.6	97.5	1.04 [0.85-1.27]
**Asthma**					
Yes	4/76 (5.3)	9/102 (8.8)	-1.2	2.8	0.65 [0.20-2.07]
No	252/1924 (13.1)	236/1909 (12.4)	6.4	102.3	1.06 [0.88-1.29]
**Corticosteroids at entry or later (ignoring any biases*)**					
Yes	206/1133 (18.2)	192/1182 (16.2)	9.1	80.6	1.12 [0.90-1.39]
No	50/867 (5.8)	53/829 (6.4)	-1.8	23.3	0.93 [0.62-1.39]
**Geographic location**					
Europe or Canada	50/227 (22.0)	39/215 (18.1)	4.7	17.0	1.32 [0.82-2.12]
Latin America	85/465 (18.3)	79/469 (16.8)	2.8	33.3	1.09 [0.77-1.53]
Asia and Africa	121/1308 (9.3)	127/1327 (9.6)	-1.8	53.6	0.97 [0.74-1.26]
**Control Type**					
Lopinavir	69/584 (11.8)	68/625 (10.9)	4.9	28.6	1.19 [0.82-1.71]
Standard care	187/1416 (13.2)	177/1386 (12.8)	1.7	76.8	1.02 [0.82-1.28]
**Scheduled Interferon regimen**					
Subcutaneous	116/1585 (7.3)	106/1604 (6.6)	6.5	50.4	1.14 [0.86-1.50]
Intravenous†	140/415 (33.7)	139/407 (34.2)	-1.7	45.1	0.96 [0.72-1.29]
**Total**	**256/2000 (12.8)**	**245/2011 (12.2)**	**6.5**	**105.7**	**1.06 [0.88-1.29]**

Heterogeneity test: χ^2_{19} = 16.8; p = 0.6

p = 0.53

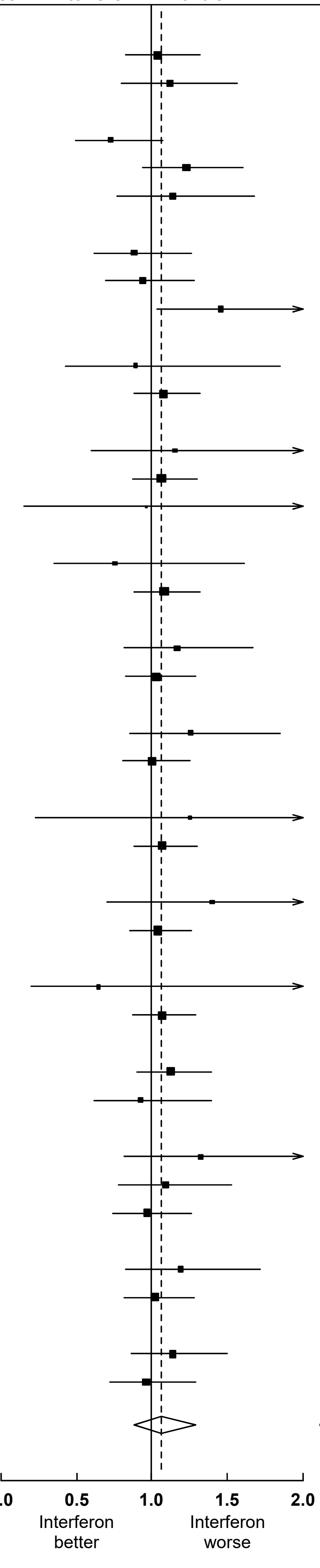

* RRs in steroid subgroups may be somewhat biased by later steroid use.

† Possible only if high-flow oxygen or ventilation was being given (and the country had been supplied with intravenous interferon)

Figure S7A-D. RRs for the composite of death in hospital or initiation of ventilation: effects of (A) remdesivir, (B) hydroxychloroquine, (C) lopinavir, (D) interferon, each vs its control

	Events reported / Patients in ITT analyses (%) Active	Control	Active-group events: log-rank statistics O-E	Variance	Event rate ratio RR, & 95% CI (Active : Control)
**(A) Remdesivir**					
**Age at entry**					
<50	177/1310 (13.5)	200/1326 (15.1)	-6.5	85.5	0.93 [0.75-1.15]
50-69	423/1920 (22.0)	440/1908 (23.1)	-16.0	191.2	0.92 [0.80-1.06]
70+	295/916 (32.2)	345/895 (38.5)	-30.8	139.6	0.80 [0.68-0.95]
**Respiratory support at entry**					
Not ventilated	744/3787 (19.6)	851/3782 (22.5)	-61.5	346.6	0.84 [0.75-0.93]
Ventilated	151/359 (42.1)	134/347 (38.6)	8.3	69.8	1.13 [0.89-1.42]
**Total**	**895/4146 (21.6)**	**985/4129 (23.9)**	**-53.3**	**416.4**	**0.88 [0.80-0.97]**
**Heterogeneity around total χ^2_3: 6.9**					**p = 0.009**
**(B) Hydroxychloroquine**					
**Age at entry**					
<50	29/336 (8.6)	32/316 (10.1)	-1.3	14.2	0.91 [0.54-1.54]
50-69	79/410 (19.3)	55/394 (14.0)	9.0	30.9	1.34 [0.94-1.90]
70+	42/202 (20.8)	48/190 (25.3)	-4.2	21.0	0.82 [0.53-1.26]
**Respiratory support at entry**					
Not ventilated	115/864 (13.3)	107/818 (13.1)	0.2	51.0	1.00 [0.76-1.32]
Ventilated	35/84 (41.7)	28/82 (34.1)	3.4	15.0	1.25 [0.76-2.08]
**Total**	**150/948 (15.8)**	**135/900 (15.0)**	**3.6**	**66.1**	**1.06 [0.83-1.34]**
**Heterogeneity around total χ^2_3: 3.9**					**p = 0.66**
**(C) Lopinavir**					
**Age at entry**					
<50	37/513 (7.2)	56/504 (11.1)	-8.4	21.9	0.68 [0.45-1.04]
50-69	112/602 (18.6)	93/594 (15.7)	6.5	46.6	1.15 [0.86-1.53]
70+	74/289 (25.6)	80/270 (29.6)	-6.3	34.9	0.83 [0.60-1.16]
**Respiratory support at entry**					
Not ventilated	189/1292 (14.6)	193/1253 (15.4)	-8.9	86.7	0.90 [0.73-1.11]
Ventilated	34/112 (30.4)	36/115 (31.3)	0.6	16.7	1.04 [0.64-1.68]
**Total**	**223/1404 (15.9)**	**229/1368 (16.7)**	**-8.2**	**103.4**	**0.92 [0.76-1.12]**
**Heterogeneity around total χ^2_3: 4.9**					**p = 0.42**
**(D) Interferon**					
**Age at entry**					
<50	84/759 (11.1)	86/731 (11.8)	-2.6	39.5	0.94 [0.68-1.28]
50-69	227/970 (23.4)	197/1017 (19.4)	22.6	94.8	1.27 [1.04-1.55]
70+	119/415 (28.7)	115/399 (28.8)	-0.8	51.3	0.99 [0.75-1.30]
**Respiratory support at entry**					
Not ventilated	361/2000 (18.0)	347/2011 (17.3)	9.1	156.3	1.06 [0.91-1.24]
Ventilated	69/144 (47.9)	51/136 (37.5)	10.0	29.3	1.41 [0.98-2.02]
**Total**	**430/2144 (20.1)**	**398/2147 (18.5)**	**19.1**	**185.6**	**1.11 [0.96-1.28]**
**Heterogeneity around total χ^2_3: 5.6**					**p = 0.16**

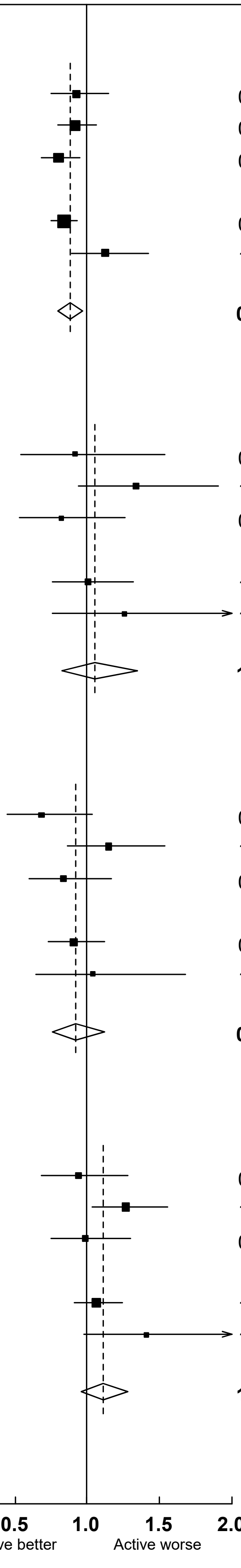

Figure S8A. RRs for the composite of death in hospital or initiation of ventilation, stratified by age and respiratory support at entry: remdesivir vs its control, by entry characteristics and by steroid use at any time*

	Events reported / Patients in ITT analyses (%)		Remdesivir events: log-rank statistics		Event rate ratio RR, & 95% CI
	Remdesivir	Control	O-E	Variance	Remdesivir : Control
**Sex**					
Male	601/2601 (23.1)	691/2639 (26.2)	-44.4	281.1	0.85 [0.76-0.96]
Female	294/1545 (19.0)	294/1490 (19.7)	-6.2	133.8	0.95 [0.81-1.13]
**Age at entry**					
<50	177/1310 (13.5)	200/1326 (15.1)	-6.5	85.5	0.93 [0.75-1.15]
50-69	423/1920 (22.0)	440/1908 (23.1)	-16.0	191.2	0.92 [0.80-1.06]
70+	295/916 (32.2)	345/895 (38.5)	-30.8	139.6	0.80 [0.68-0.95]
**Days from hospital admission to randomisation**					
0	178/888 (20.0)	182/892 (20.4)	-3.4	78.9	0.96 [0.77-1.19]
1	275/1462 (18.8)	356/1459 (24.4)	-45.2	138.3	0.72 [0.61-0.85]
2+	442/1796 (24.6)	447/1778 (25.1)	-5.1	197.6	0.97 [0.85-1.12]
**Respiratory support at entry**					
No O_2	52/869 (6.0)	58/861 (6.7)	-2.8	26.3	0.90 [0.61-1.32]
Low-flow or high-flow O_2	692/2918 (23.7)	793/2921 (27.1)	-58.8	320.3	0.83 [0.75-0.93]
Ventilation	151/359 (42.1)	134/347 (38.6)	8.3	69.8	1.13 [0.89-1.42]
**Bilateral lung lesions**					
Not imaged at entry	104/399 (26.1)	101/417 (24.2)	-0.2	44.1	1.00 [0.74-1.34]
Yes	749/3326 (22.5)	828/3341 (24.8)	-39.5	349.3	0.89 [0.80-0.99]
No	42/421 (10.0)	56/371 (15.1)	-11.6	22.0	0.59 [0.39-0.90]
**Current smoking**					
Yes	47/247 (19.0)	57/233 (24.5)	-7.7	22.0	0.70 [0.46-1.07]
No	848/3899 (21.7)	928/3896 (23.8)	-44.1	393.7	0.89 [0.81-0.99]
**Diabetes**					
Yes	275/1129 (24.4)	311/1120 (27.8)	-25.8	127.4	0.82 [0.69-0.97]
No	620/3017 (20.6)	674/3009 (22.4)	-28.7	288.4	0.91 [0.81-1.02]
**Heart disease**					
Yes	222/929 (23.9)	286/935 (30.6)	-35.3	111.2	0.73 [0.60-0.88]
No	673/3217 (20.9)	699/3194 (21.9)	-18.6	304.2	0.94 [0.84-1.05]
**Chronic liver disease**					
Yes	18/57 (31.6)	24/72 (33.3)	-1.5	8.2	0.83 [0.42-1.64]
No	877/4089 (21.4)	961/4057 (23.7)	-52.2	406.8	0.88 [0.80-0.97]
**Chronic lung disease**					
Yes	82/284 (28.9)	90/281 (32.0)	-5.2	37.3	0.87 [0.63-1.20]
No	813/3862 (21.1)	895/3848 (23.3)	-48.6	378.6	0.88 [0.80-0.97]
**Asthma**					
Yes	49/247 (19.8)	60/242 (24.8)	-4.1	23.2	0.84 [0.56-1.26]
No	846/3899 (21.7)	925/3887 (23.8)	-49.9	392.5	0.88 [0.80-0.97]
**Corticosteroids at entry or later (ignoring any biases*)**					
Yes	722/2782 (26.0)	830/2820 (29.4)	-52.3	336.7	0.86 [0.77-0.95]
No	173/1363 (12.7)	155/1309 (11.8)	1.1	75.9	1.01 [0.81-1.27]
**Geographic location‡**					
Europe or Canada	372/1649 (22.6)	442/1594 (27.7)	-44.8	172.1	0.77 [0.66-0.90]
Latin America	161/558 (28.9)	199/593 (33.6)	-12.3	79.2	0.86 [0.69-1.07]
Asia and Africa	362/1939 (18.7)	344/1942 (17.7)	1.7	159.5	1.01 [0.87-1.18]
**■ Total**	**895/4146 (21.6)**	**985/4129 (23.9)**	**-53.3**	**416.4**	**0.88 [0.80-0.97] p = 0.009**

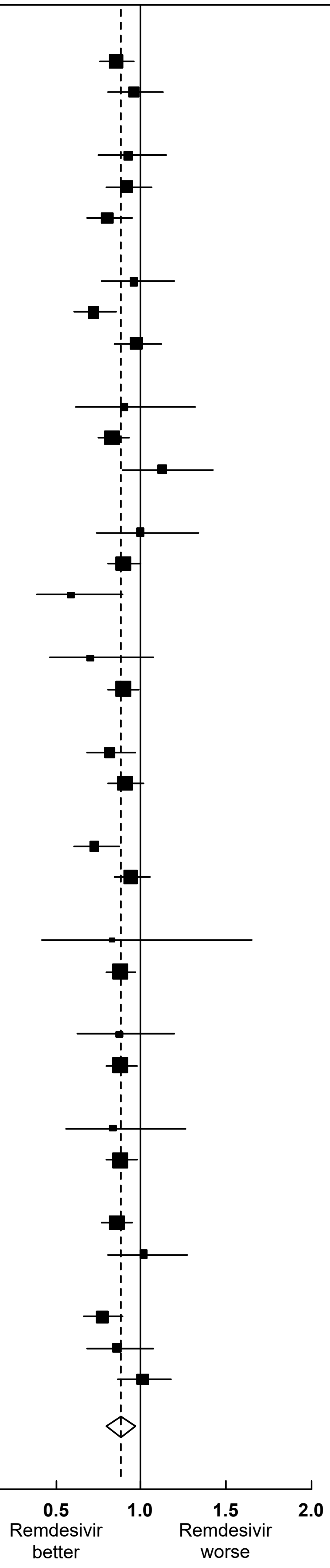

Heterogeneity test: χ^2_{18} = 36.0; p = 0.007

* RRs in steroid subgroups may be somewhat biased by later steroid use.

‡ Wastefully fine stratification by country and, within country, by the 9 strata of age and ventilation yielded RR=0.89, 95% CI 0.81-0.98, with no good evidence of between-country RR heterogeneity.

Figure S8B. RRs for the composite of death in hospital or initiation of ventilation, stratified by age and respiratory support at entry: hydroxychloroquine vs its control, by entry characteristics and by steroid use at any time*

	Events reported / Patients in ITT analyses (%)		Hydroxychlor. events: log-rank statistics		Event rate ratio RR, & 95% CI
	Hydroxychloroquine	Control	O-E	Variance	Hydroxychlor. : Control
**Sex**					
Male	106/574 (18.5)	82/532 (15.4)	8.5	43.3	1.22 [0.90-1.64]
Female	44/374 (11.8)	53/368 (14.4)	-5.0	22.6	0.80 [0.53-1.21]
**Age at entry**					
<50	29/336 (8.6)	32/316 (10.1)	-1.3	14.2	0.91 [0.54-1.54]
50-69	79/410 (19.3)	55/394 (14.0)	9.0	30.9	1.34 [0.94-1.90]
70+	42/202 (20.8)	48/190 (25.3)	-4.2	21.0	0.82 [0.53-1.26]
**Days from hospital admission to randomisation**					
0	35/296 (11.8)	27/280 (9.6)	3.2	14.7	1.25 [0.75-2.08]
1	55/319 (17.2)	54/307 (17.6)	-1.8	24.0	0.93 [0.62-1.39]
2+	60/333 (18.0)	54/313 (17.3)	1.7	26.2	1.07 [0.73-1.56]
**Respiratory support at entry**					
No O_2	16/346 (4.6)	11/338 (3.3)	2.2	6.6	1.40 [0.65-3.01]
Low-flow or high-flow O_2	99/518 (19.1)	96/480 (20.0)	-2.0	44.5	0.96 [0.71-1.28]
Ventilation	35/84 (41.7)	28/82 (34.1)	3.4	15.0	1.25 [0.76-2.08]
**Bilateral lung lesions**					
Not imaged at entry	34/116 (29.3)	30/106 (28.3)	-0.1	13.4	0.99 [0.58-1.69]
Yes	111/714 (15.5)	103/670 (15.4)	1.5	49.9	1.03 [0.78-1.36]
No	5/118 (4.2)	2/124 (1.6)	1.3	1.6	2.24 [0.49-10.34]
**Current smoking**					
Yes	14/93 (15.1)	13/82 (15.9)	1.2	4.9	1.27 [0.52-3.08]
No	136/855 (15.9)	122/818 (14.9)	3.6	59.5	1.06 [0.82-1.37]
**Diabetes**					
Yes	45/201 (22.4)	35/205 (17.1)	4.8	18.4	1.29 [0.82-2.04]
No	105/747 (14.1)	100/695 (14.4)	0.2	47.1	1.00 [0.75-1.34]
**Heart disease**					
Yes	29/194 (14.9)	34/194 (17.5)	-3.2	14.7	0.81 [0.48-1.34]
No	121/754 (16.0)	101/706 (14.3)	6.4	50.9	1.13 [0.86-1.49]
**Chronic liver disease**					
Yes	2/15 (13.3)	4/14 (28.6)	-2.0	1.1	0.18 [0.03-1.12]
No	148/933 (15.9)	131/886 (14.8)	4.8	64.6	1.08 [0.84-1.37]
**Chronic lung disease**					
Yes	11/63 (17.5)	10/65 (15.4)	0.4	4.9	1.08 [0.45-2.63]
No	139/885 (15.7)	125/835 (15.0)	2.7	61.0	1.05 [0.81-1.34]
**Asthma**					
Yes	6/44 (13.6)	5/47 (10.6)	0.8	2.0	1.51 [0.37-6.11]
No	144/904 (15.9)	130/853 (15.2)	2.5	63.5	1.04 [0.81-1.33]
**Corticosteroids at entry or later (ignoring any biases*)**					
Yes	52/190 (27.4)	60/197 (30.5)	-2.6	24.5	0.90 [0.61-1.34]
No	98/758 (12.9)	75/703 (10.7)	6.8	40.3	1.18 [0.87-1.61]
**Geographic location‡**					
Europe or Canada	49/287 (17.1)	43/266 (16.2)	1.7	20.2	1.09 [0.70-1.69]
Latin America	35/97 (36.1)	29/96 (30.2)	2.6	13.9	1.21 [0.72-2.04]
Asia and Africa	66/564 (11.7)	63/538 (11.7)	0.8	30.2	1.03 [0.72-1.47]
**Scheduled Hydroxychloroquine dose**					
Full dose	120/799 (15.0)	104/751 (13.8)	5.3	51.8	1.11 [0.84-1.45]
Half dose (Discovery)	30/149 (20.1)	31/149 (20.8)	-1.1	13.2	0.92 [0.54-1.58]
**Total**	**150/948 (15.8)**	**135/900 (15.0)**	**3.6**	**66.1**	**1.06 [0.83-1.34]** p = 0.66

Heterogeneity test: χ^2_{19} = 17.1; p = 0.58

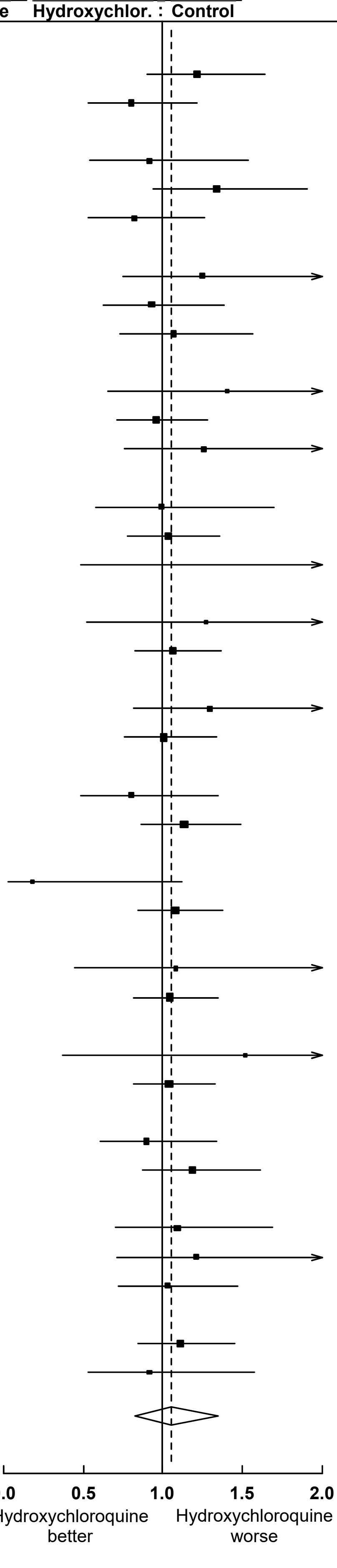

* RRs in steroid subgroups may be somewhat biased by later steroid use.

‡ Wastefully fine stratification by country and, within country, by the 9 strata of age and ventilation yielded RR=1.09, 95% CI 0.85-1.41, with no good evidence of between-country RR heterogeneity.

Figure S8C. RRs for the composite of death in hospital or initiation of ventilation, stratified by age and respiratory support at entry: lopinavir vs its control, by entry characteristics and by steroid use at any time*

	Events reported / Patients in ITT analyses (%)		Lopinavir events: log-rank statistics		Event rate ratio RR, & 95% CI
	Lopinavir	Control	O-E	Variance	Lopinavir : Control
**Sex**					
Male	149/852 (17.5)	145/800 (18.1)	-3.0	65.6	0.95 [0.75-1.22]
Female	74/552 (13.4)	84/568 (14.8)	-5.5	37.0	0.86 [0.62-1.19]
**Age at entry**					
<50	37/513 (7.2)	56/504 (11.1)	-8.4	21.9	0.68 [0.45-1.04]
50-69	112/602 (18.6)	93/594 (15.7)	6.5	46.6	1.15 [0.86-1.53]
70+	74/289 (25.6)	80/270 (29.6)	-6.3	34.9	0.83 [0.60-1.16]
**Days from hospital admission to randomisation**					
0	55/422 (13.0)	46/401 (11.5)	4.0	23.4	1.19 [0.79-1.78]
1	76/450 (16.9)	78/444 (17.6)	-4.8	34.7	0.87 [0.62-1.22]
2+	92/532 (17.3)	105/523 (20.1)	-4.3	44.1	0.91 [0.68-1.22]
**Respiratory support at entry**					
No O_2	31/527 (5.9)	30/535 (5.6)	1.5	14.7	1.11 [0.66-1.84]
Low-flow or high-flow O_2	158/765 (20.7)	163/718 (22.7)	-10.3	72.0	0.87 [0.69-1.09]
Ventilation	34/112 (30.4)	36/115 (31.3)	0.6	16.7	1.04 [0.64-1.68]
**Bilateral lung lesions**					
Not imaged at entry	43/163 (26.4)	42/157 (26.8)	-2.1	18.4	0.89 [0.56-1.41]
Yes	173/1048 (16.5)	175/1001 (17.5)	-4.9	80.0	0.94 [0.76-1.17]
No	7/193 (3.6)	12/210 (5.7)	-1.5	4.0	0.69 [0.26-1.84]
**Current smoking**					
Yes	17/137 (12.4)	18/121 (14.9)	-1.4	7.2	0.82 [0.40-1.70]
No	206/1267 (16.3)	211/1247 (16.9)	-7.1	95.4	0.93 [0.76-1.13]
**Diabetes**					
Yes	64/346 (18.5)	56/326 (17.2)	-1.6	26.5	0.94 [0.64-1.38]
No	159/1058 (15.0)	173/1042 (16.6)	-6.9	76.0	0.91 [0.73-1.14]
**Heart disease**					
Yes	58/292 (19.9)	54/291 (18.6)	0.9	25.1	1.04 [0.70-1.54]
No	165/1112 (14.8)	175/1077 (16.2)	-9.5	78.0	0.88 [0.71-1.10]
**Chronic liver disease**					
Yes	3/16 (18.8)	6/23 (26.1)	-0.3	1.5	0.81 [0.16-4.04]
No	220/1388 (15.9)	223/1345 (16.6)	-7.7	101.3	0.93 [0.76-1.13]
**Chronic lung disease**					
Yes	18/94 (19.1)	16/86 (18.6)	0.4	7.5	1.06 [0.52-2.17]
No	205/1310 (15.6)	213/1282 (16.6)	-8.5	95.4	0.92 [0.75-1.12]
**Asthma**					
Yes	10/68 (14.7)	10/57 (17.5)	0.0	3.2	0.99 [0.33-2.94]
No	213/1336 (15.9)	219/1311 (16.7)	-7.2	99.0	0.93 [0.76-1.13]
**Corticosteroids at entry or later (ignoring any biases*)**					
Yes	109/367 (29.7)	133/387 (34.4)	-8.9	50.8	0.84 [0.64-1.10]
No	114/1036 (11.0)	96/981 (9.8)	3.2	49.3	1.07 [0.81-1.41]
**Geographic location‡**					
Europe or Canada	81/348 (23.3)	83/353 (23.5)	0.2	35.2	1.01 [0.72-1.40]
Latin America	39/147 (26.5)	48/148 (32.4)	-3.7	19.1	0.82 [0.53-1.29]
Asia and Africa	103/909 (11.3)	98/867 (11.3)	-1.4	46.7	0.97 [0.73-1.29]
**Total**	**223/1404 (15.9)**	**229/1368 (16.7)**	**-8.2**	**103.4**	**0.92 [0.76-1.12]** p = 0.42

Heterogeneity test: χ^2_{18}= 10.5; p = 0.91

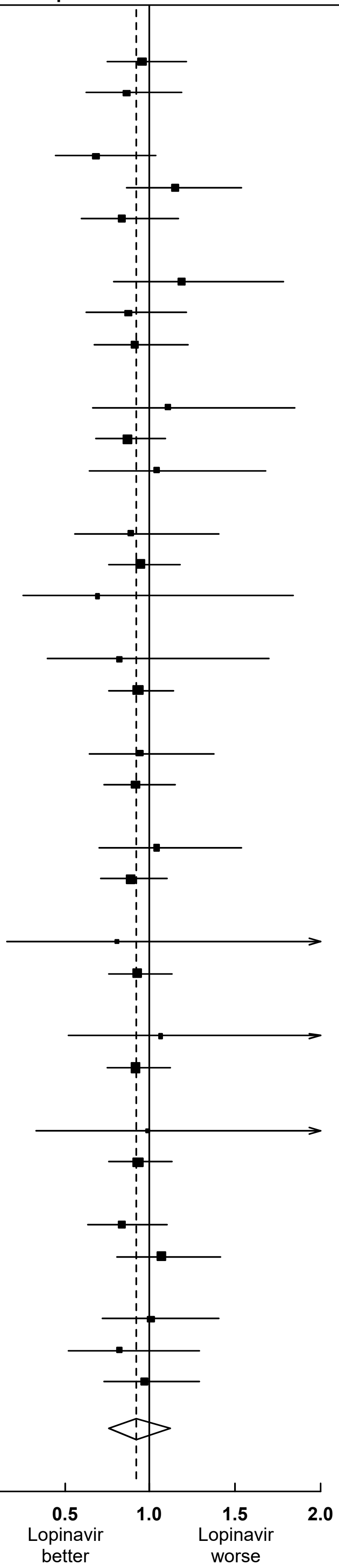

* RRs in steroid subgroups may be somewhat biased by later steroid use.

‡ Wastefully fine stratification by country and, within country, by the 9 strata of age and ventilation yielded RR=0.91, 95% CI 0.75-1.12, with no good evidence of between-country RR heterogeneity.

Figure S8D. RRs for the composite of death in hospital or initiation of ventilation, stratified by age and respiratory support at entry: interferon vs its control, by entry characteristics and by steroid use at any time*

	Events reported / Patients in ITT analyses (%)		Interferon events: log-rank statistics		Event rate ratio RR, & 95% CI
	Interferon	Control	O-E	Variance	Interferon : Control
**Sex**					
Male	288/1342 (21.5)	272/1331 (20.4)	9.0	123.5	1.08 [0.90-1.28]
Female	142/802 (17.7)	126/816 (15.4)	10.0	61.0	1.18 [0.92-1.51]
**Age at entry**					
<50	84/759 (11.1)	86/731 (11.8)	-2.6	39.5	0.94 [0.68-1.28]
50-69	227/970 (23.4)	197/1017 (19.4)	22.6	94.8	1.27 [1.04-1.55]
70+	119/415 (28.7)	115/399 (28.8)	-0.8	51.3	0.99 [0.75-1.30]
**Days from hospital admission to randomisation**					
0	117/707 (16.5)	120/702 (17.1)	0.6	53.1	1.01 [0.77-1.32]
1	141/700 (20.1)	150/699 (21.5)	-6.1	62.1	0.91 [0.71-1.16]
2+	172/737 (23.3)	128/746 (17.2)	24.6	68.1	1.44 [1.13-1.82]
**Respiratory support at entry**					
No O_2	22/503 (4.4)	25/508 (4.9)	-1.3	11.5	0.90 [0.50-1.60]
Low-flow or high-flow O_2	339/1497 (22.6)	322/1503 (21.4)	10.4	144.8	1.07 [0.91-1.26]
Ventilation	69/144 (47.9)	51/136 (37.5)	10.0	29.3	1.41 [0.98-2.02]
**Bilateral lung lesions**					
Not imaged at entry	42/159 (26.4)	43/160 (26.9)	0.2	18.0	1.01 [0.64-1.60]
Yes	383/1857 (20.6)	348/1865 (18.7)	18.7	163.8	1.12 [0.96-1.31]
No	5/128 (3.9)	7/122 (5.7)	-0.3	2.4	0.89 [0.25-3.18]
**Current smoking**					
Yes	25/142 (17.6)	27/147 (18.4)	-1.3	9.8	0.88 [0.47-1.64]
No	405/2002 (20.2)	371/2000 (18.6)	20.0	174.6	1.12 [0.97-1.30]
**Diabetes**					
Yes	120/517 (23.2)	103/563 (18.3)	13.3	48.3	1.32 [0.99-1.74]
No	310/1627 (19.1)	295/1584 (18.6)	4.6	136.0	1.03 [0.87-1.22]
**Heart disease**					
Yes	104/457 (22.8)	100/487 (20.5)	5.1	44.7	1.12 [0.84-1.50]
No	326/1687 (19.3)	298/1660 (18.0)	14.7	139.3	1.11 [0.94-1.31]
**Chronic liver disease**					
Yes	7/14 (50.0)	7/24 (29.2)	1.0	1.9	1.69 [0.41-6.89]
No	423/2130 (19.9)	391/2123 (18.4)	18.1	182.6	1.10 [0.96-1.28]
**Chronic lung disease**					
Yes	37/120 (30.8)	30/110 (27.3)	4.8	13.9	1.41 [0.83-2.38]
No	393/2024 (19.4)	368/2037 (18.1)	15.1	171.1	1.09 [0.94-1.27]
**Asthma**					
Yes	7/79 (8.9)	18/105 (17.1)	-3.5	5.4	0.52 [0.22-1.22]
No	423/2065 (20.5)	380/2042 (18.6)	21.3	179.4	1.13 [0.97-1.30]
**Corticosteroids at entry or later (ignoring any biases*)**					
Yes	332/1232 (26.9)	305/1286 (23.7)	20.7	138.7	1.16 [0.98-1.37]
No	98/912 (10.7)	93/861 (10.8)	0.1	44.6	1.00 [0.75-1.34]
**Geographic location‡**					
Europe or Canada	67/268 (25.0)	56/256 (21.9)	4.3	25.3	1.18 [0.80-1.75]
Latin America	165/493 (33.5)	145/495 (29.3)	12.2	68.2	1.20 [0.94-1.52]
Asia and Africa	198/1383 (14.3)	197/1396 (14.1)	3.6	89.2	1.04 [0.85-1.28]
**Control Type**					
Lopinavir	122/648 (18.8)	112/677 (16.5)	10.0	52.1	1.21 [0.92-1.59]
Standard care	308/1496 (20.6)	286/1470 (19.5)	11.8	132.5	1.09 [0.92-1.30]
**Scheduled Interferon regimen**					
Subcutaneous	241/1679 (14.4)	212/1683 (12.6)	15.0	106.6	1.15 [0.95-1.39]
Intravenous†	189/465 (40.6)	186/464 (40.1)	2.1	68.1	1.03 [0.81-1.31]
**Total**	**430/2144 (20.1)**	**398/2147 (18.5)**	**19.1**	**185.6**	**1.11 [0.96-1.28]**
					**p = 0.16**

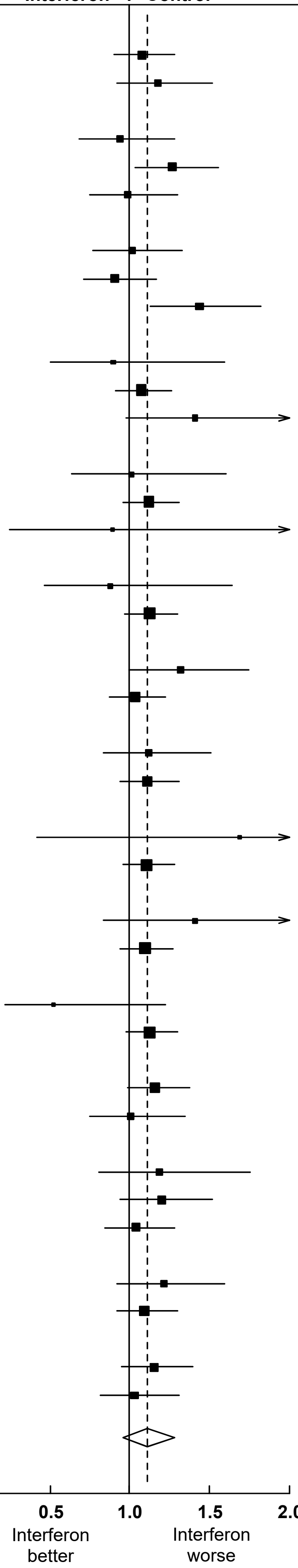

Heterogeneity test: χ^2_{20}= 23.4; p = 0.27

* RRs in steroid subgroups may be somewhat biased by later steroid use.

† Possible only if high-flow oxygen or ventilation was being given (and the country had been supplied with intravenous interferon)

‡ Wastefully fine stratification by country and, within country, by the 9 strata of age and ventilation yielded RR=1.13, 95% CI 0.97-1.31, with no good evidence of between-country RR heterogeneity.

Figure S9A-D. Remdesivir, Hydroxychloroquine, Lopinavir & Interferon, each vs its own control - effects on time to discharge alive in patients NOT being ventilated (no O_2, or low-flow / high-flow O_2) at entry Denominators: all who entered. Verticals: ends of scheduled treatment durations if still in hospital

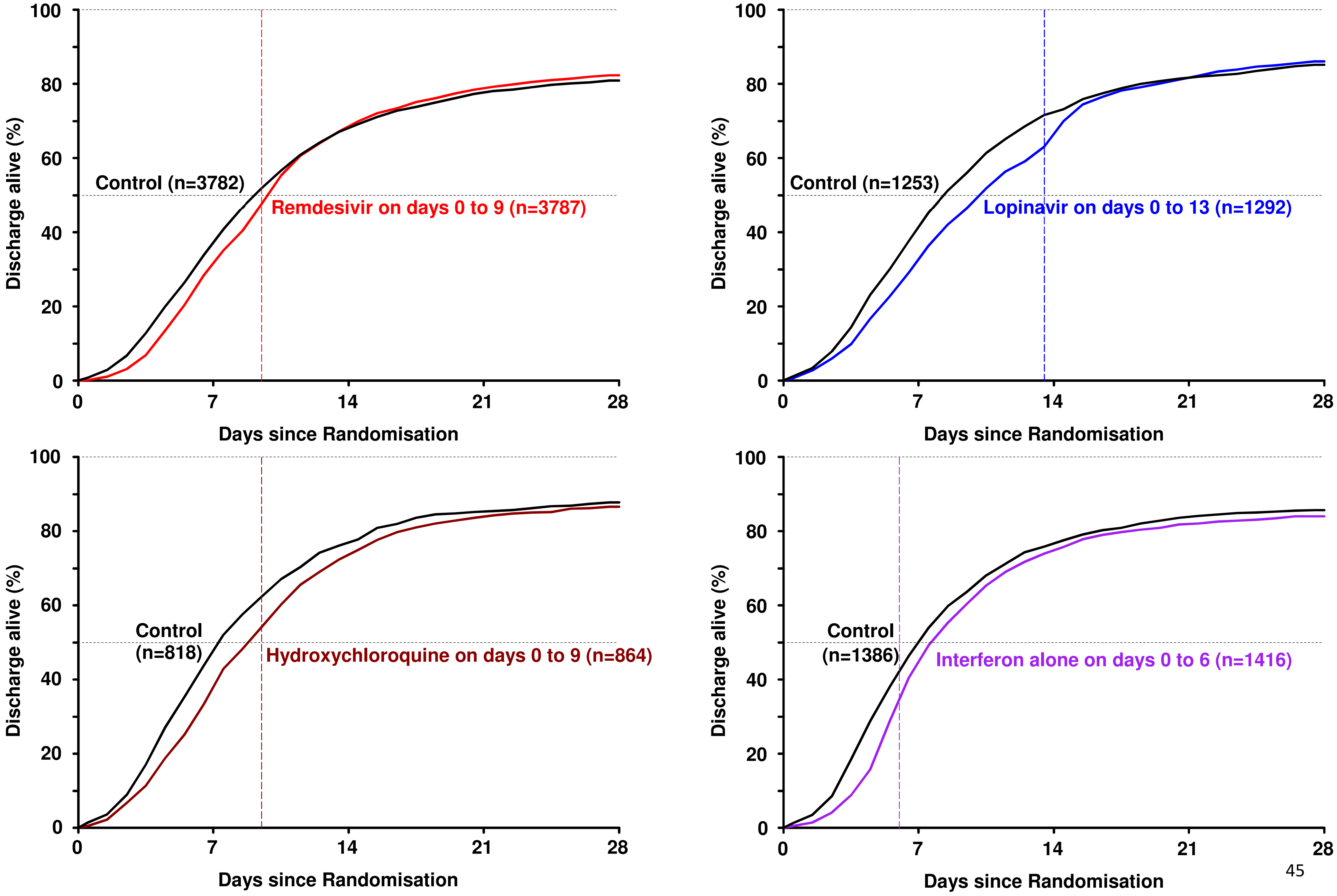

Figure S10A-D. Remdesivir, hydroxychloroquine, lopinavir & interferon, each vs its own control - effects on time to discharge alive in patients already being ventilated at entry Denominators: all who entered. Verticals: ends of scheduled treatment durations if still in hospital

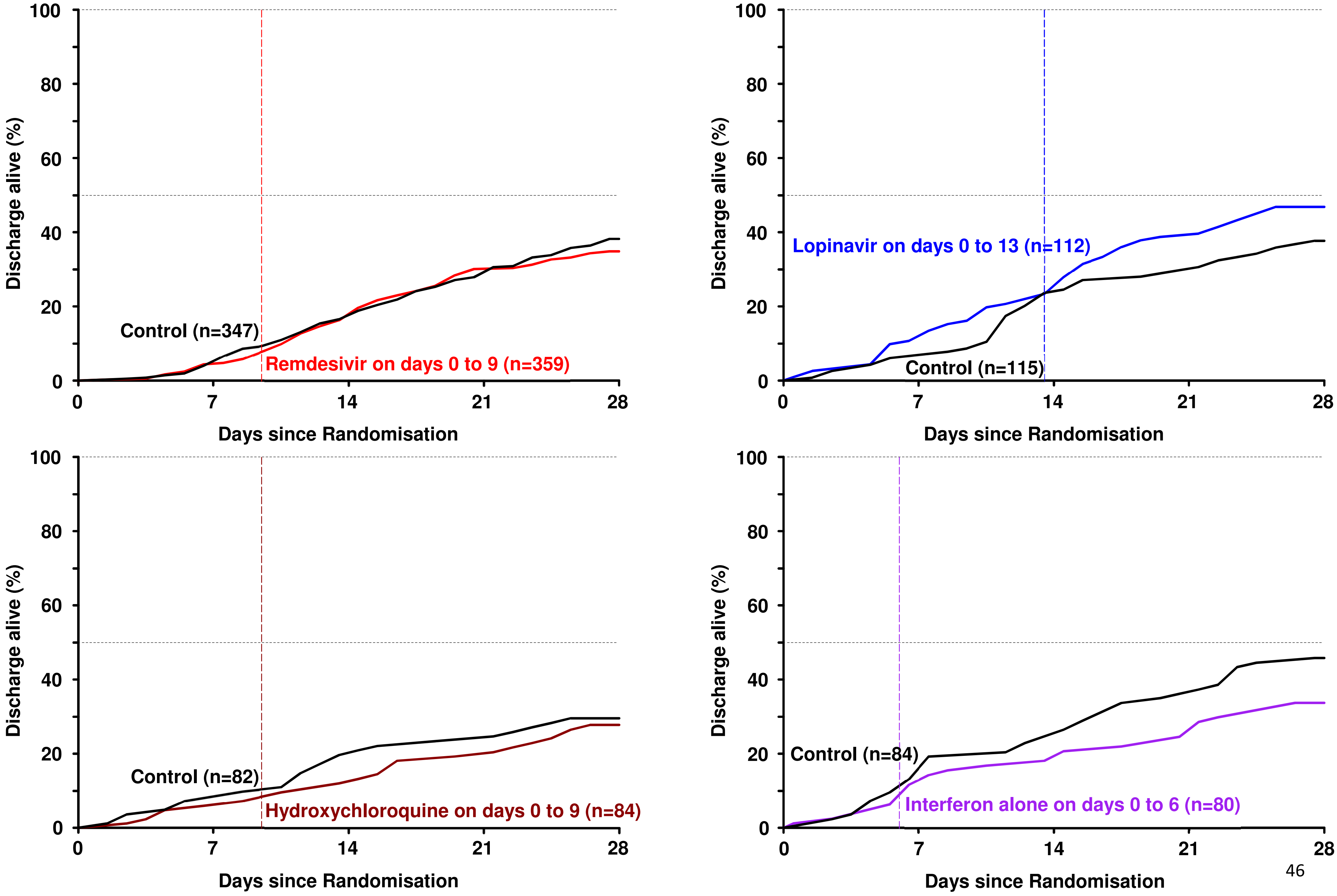

Figure S11A-D. Remdesivir, hydroxychloroquine, lopinavir & interferon, each vs its own controls - effects on time to discharge alive in patients being given low-flow O_2 / high-flow O_2 at entry Denominators: all who entered. Verticals: ends of scheduled treatment durations if still in hospital

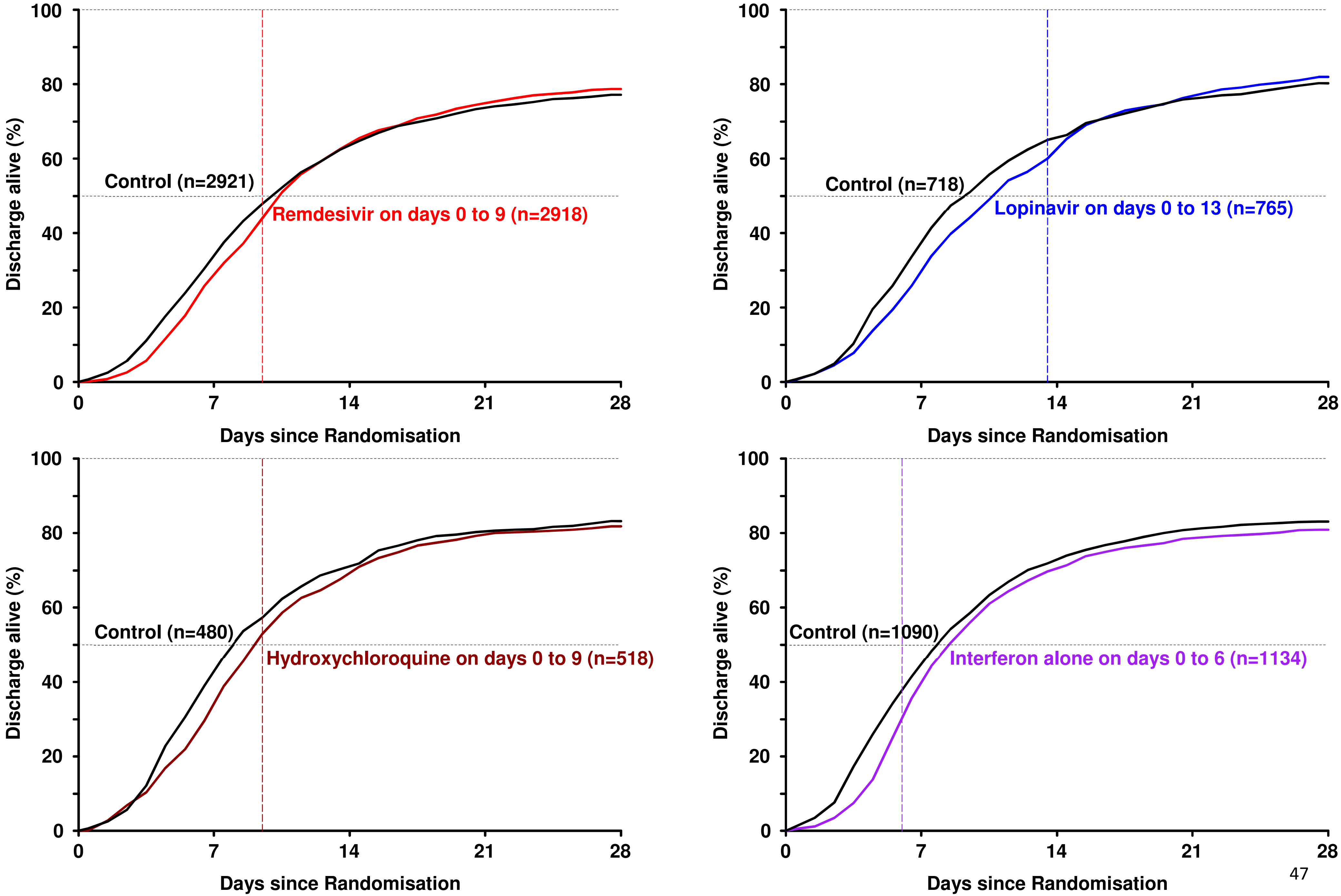

Figure S12A-D. Remdesivir, hydroxychloroquine, lopinavir & interferon, each vs its own controls - effects on time to discharge alive in patients being given no O_2 at entry (Approximates "mild-to-moderate" in ACTT-1 & FDA reports.) Denominators: all who entered. Verticals: ends of scheduled treatment durations if still in hospital

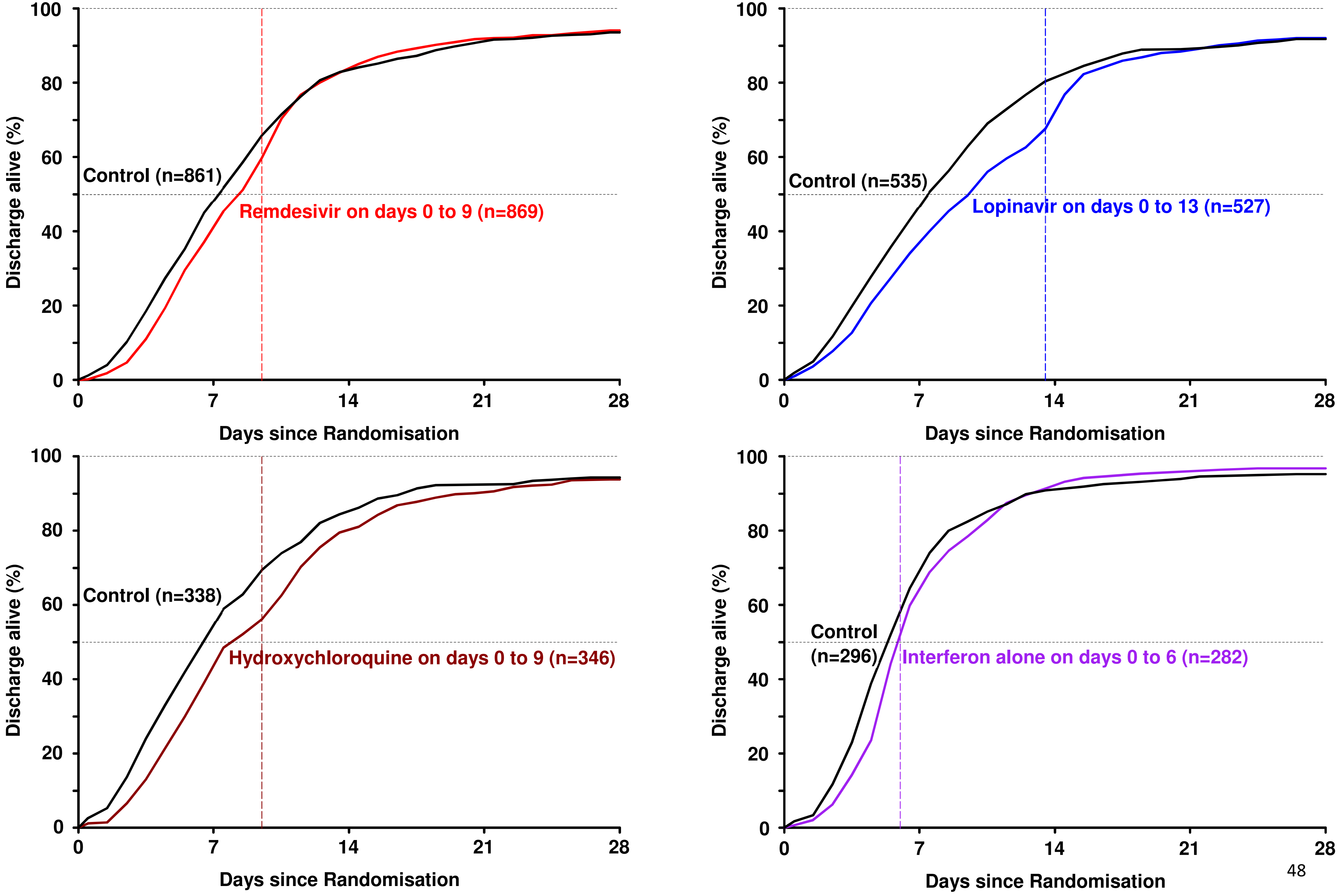

Figure S13A-D. Remdesivir, hydroxychloroquine, lopinavir & interferon, each vs its own controls - effects on time to discharge alive in patients on low/high-flow O_2 or ventilated Denominators: all who entered. Verticals: ends of scheduled treatment durations if still in hospital

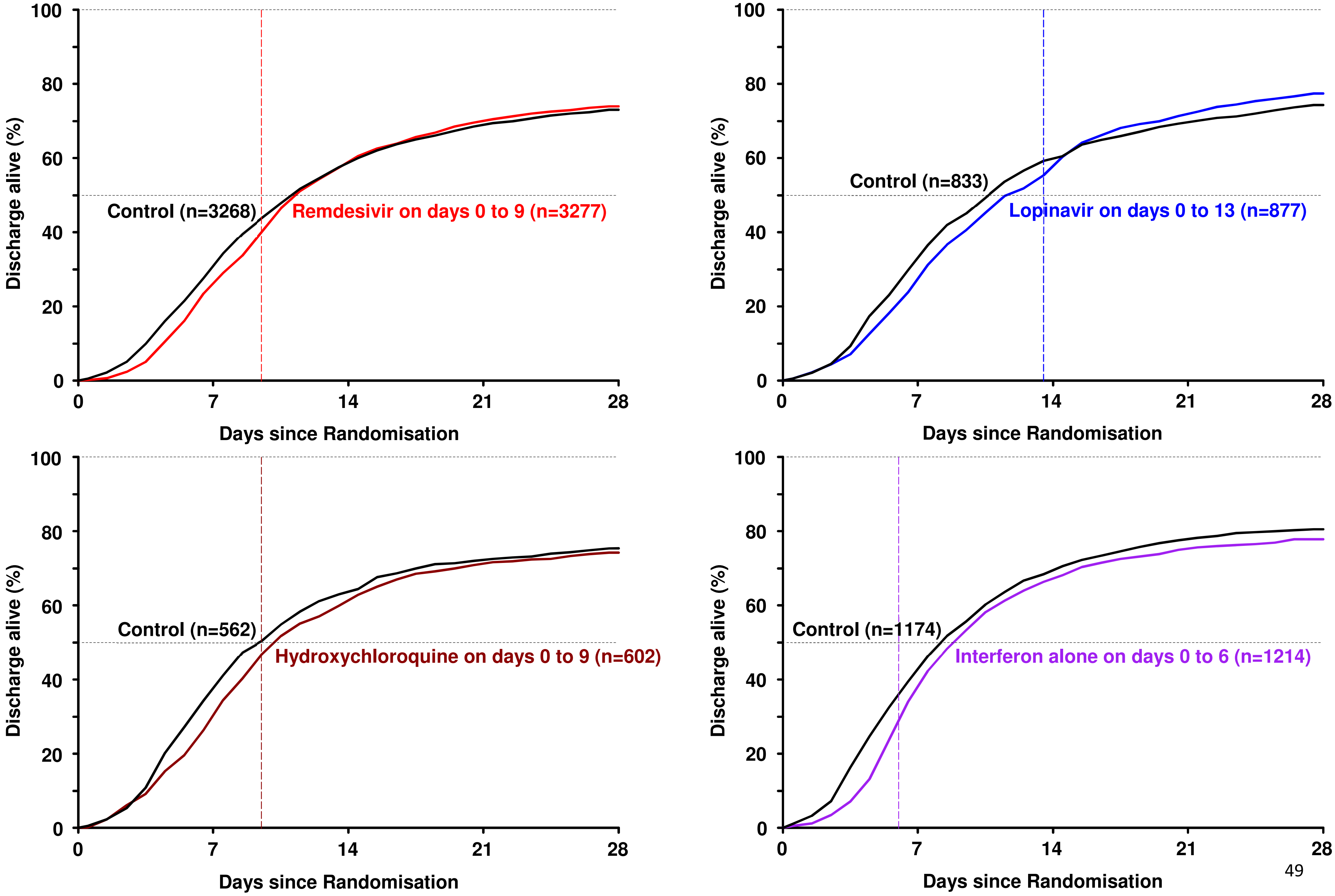

Figure S14A-D. Remdesivir, hydroxychloroquine, lopinavir & interferon, each vs its own controls - effects on time to discharge alive in all patients, regardless of respiratory support at entry Denominators: all who entered. Verticals: ends of scheduled treatment durations if still in hospital

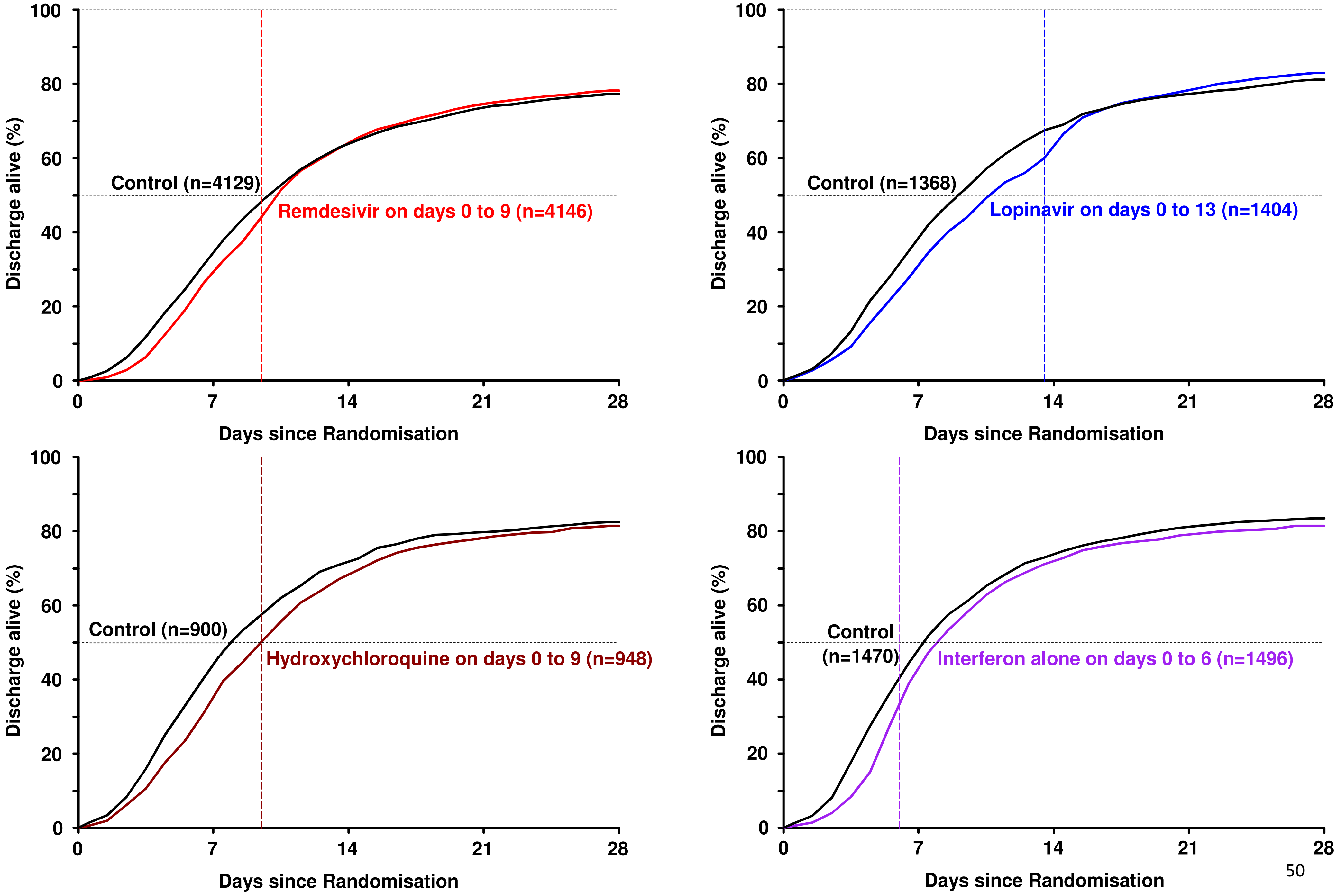

Figure S15A-D. Pairwise randomised comparisons between pairs of study drugs - effects on time to discharge alive, restricted to patients randomised where both of those two drugs were available Denominators: all who entered. Verticals: ends of scheduled treatment durations if still in hospital

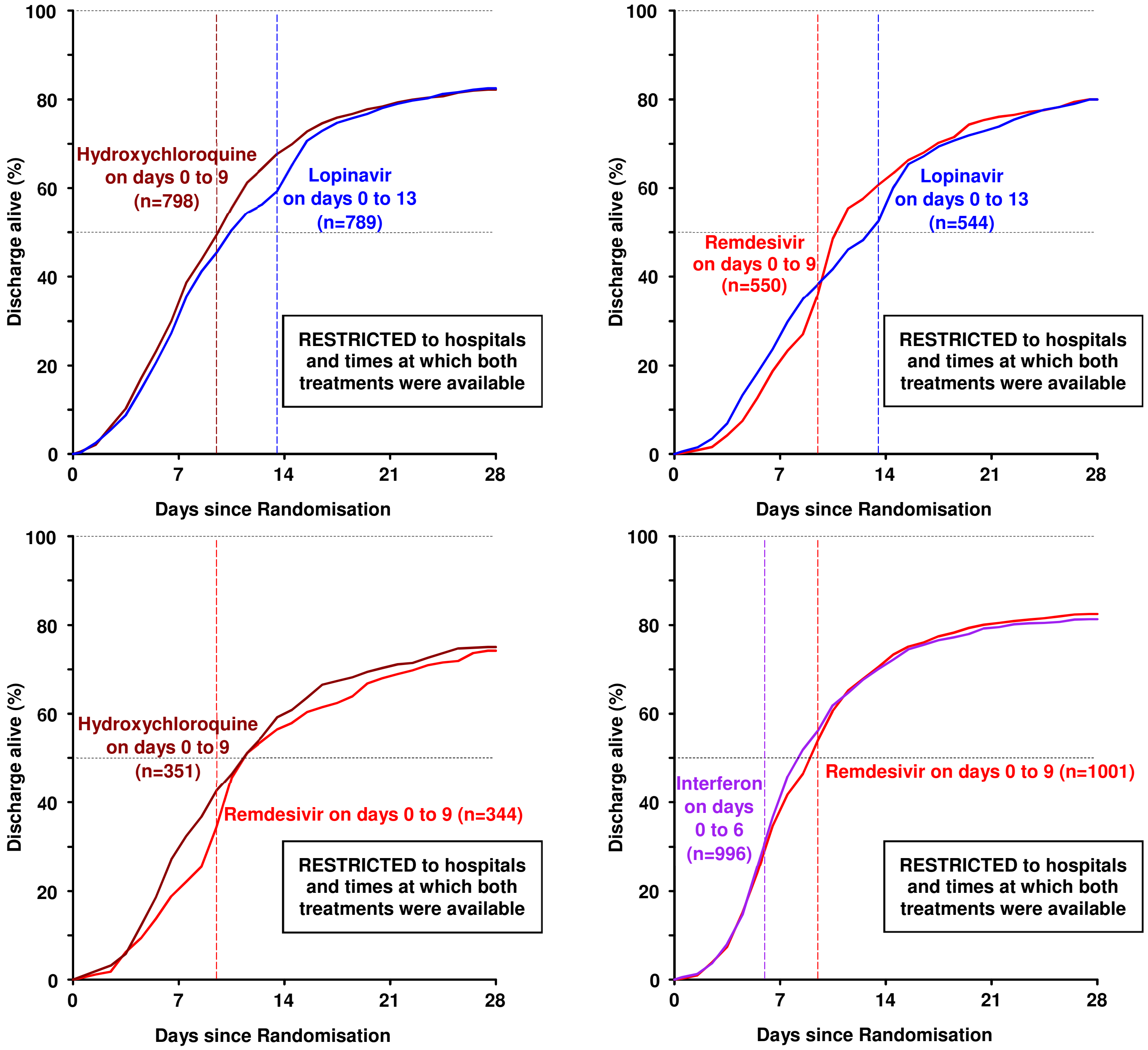

Figure S16A-D. Effects of (A) remdesivir, (B) hydroxychloroquine, (C) lopinavir, (D) interferon on cardiac death in hospital
(any death in hospital for which the trial's electronic death report included a cardiac cause)

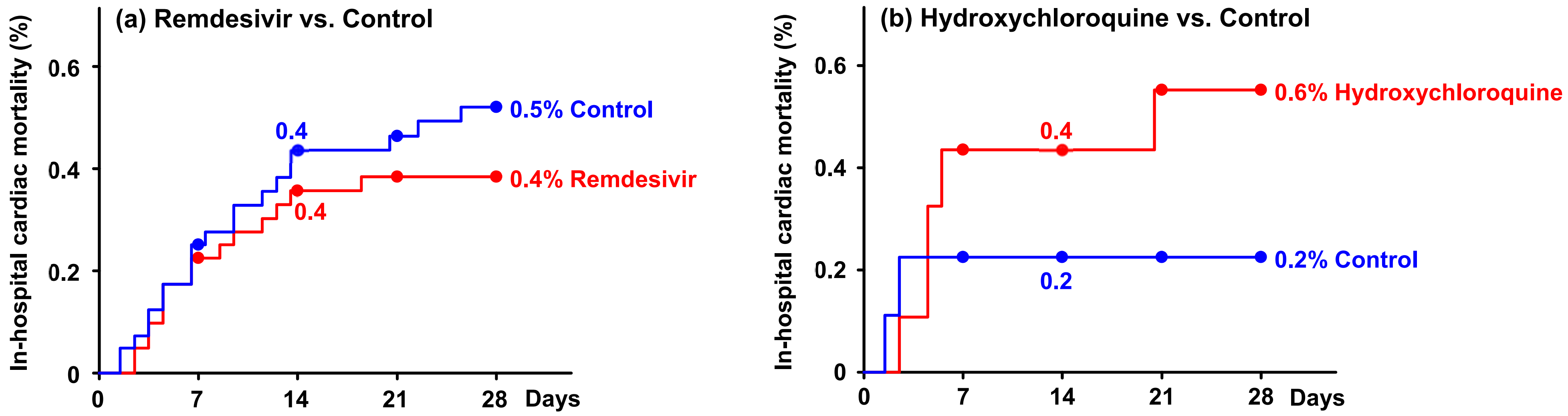

No. randomised, nos. dying, and denominators

Remdesivir	4146	9	3878	5	3703	1	3606	0	3554	2
Control	4129	10	3861	7	3665	1	3557	2	3490	0

No. randomised, nos. dying, and denominators

Hydroxychlor.	948	4	890	0	858	1	845	0	841	4
Control	900	2	848	0	820	0	812	0	808	1

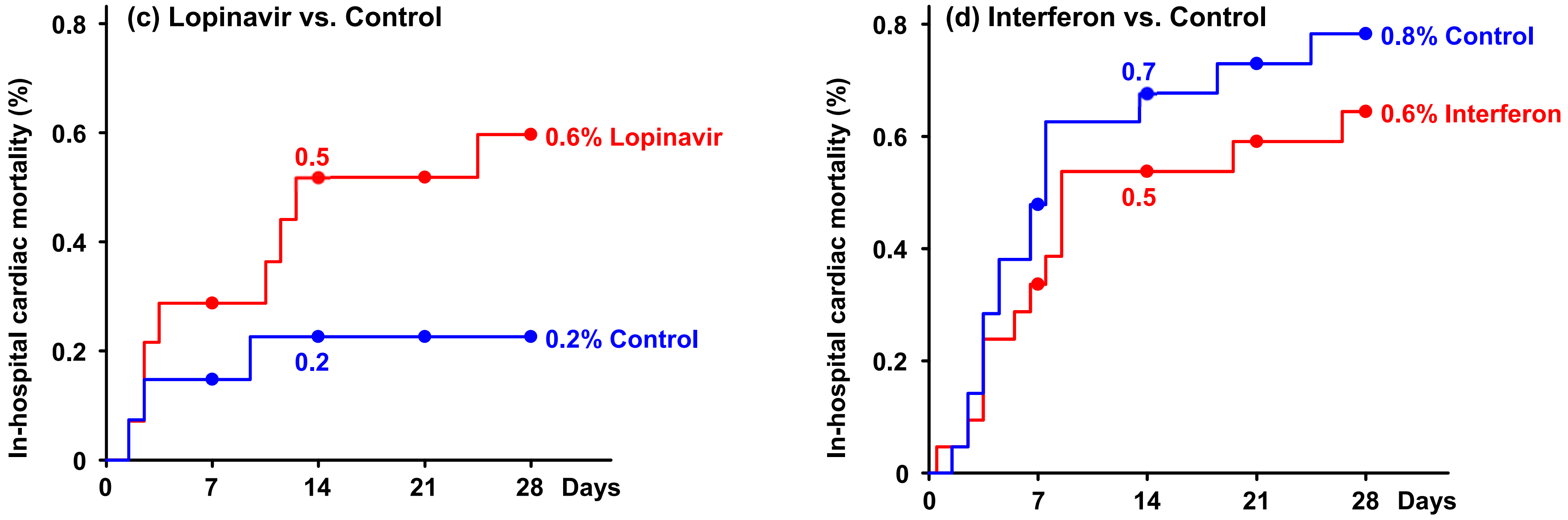

No. randomised, nos. dying, and denominators

Lopinavir	1404	4	1334	3	1286	0	1263	1	1250	0
Control	1368	2	1292	1	1242	0	1220	0	1208	0

No. randomised, nos. dying, and denominators

Interferon	2144	7	1999	4	1904	1	1859	1	1828	1
Control	2147	10	2015	4	1939	1	1904	1	1880	0

Figure S17A: Remdesivir vs its control in hospitalised COVID – Meta-analysis of mortality in Solidarity, ACTT-1, & other randomised trials with some deaths

Solidarity includes in-hospital deaths before or after day 28, but other trials may stop at day 28. Statistical analyses (O-E, RR, etc) are stratified for respiratory support, but the overall % is not. The French, Canadian and Norwegian parts of Solidarity, published separately, are already included in this meta-analysis, so they are not additional to it.

Trial, and respiratory support reported at the time of randomisation	Deaths reported / No. of patients (%): Remdesivir	Deaths reported / No. of patients (%): Control	Remdesivir deaths: Observed-Expected (O-E)**	Var (O-E)	Death rate ratio RR, and 95% CI (Remdesivir : Control)
**Solidarity (in-hospital mortality; controls not given placebo): trend p = 0.05**					
No oxygen	25/869 (2.9)	33/861 (3.8)	-3.9	14.4	0.76 [0.46-1.28]
Oxygen, not ventilated*	426/2918 (14.6)	476/2921 (16.3)	-32.0	223.5	0.87 [0.76-0.99]
Ventilated	151/359 (42.1)	134/347 (38.6)	8.3	69.8	1.13 [0.89-1.42]
**Subtotal, with stratified analyses**	**602/4146 (14.5)**	**643/4129 (15.6)**	**-27.6**	**307.7**	**0.91 [0.82-1.02]**
**ACTT-1 (28-day mortality; controls given placebo infusions): trend p = 0.03**					
No oxygen	3/75 (4.0)	3/63 (4.8)	-0.3	1.5	0.82 [0.17-4.01]
Low-flow oxygen	9/232 (3.9)	25/203 (12.3)	-8.0	6.7	0.30 [0.14-0.64]
High-flow oxygen or non-invasive ventilation	19/95 (20.0)	20/98 (20.4)	0.2	9.6	1.02 [0.54-1.92]
Invasive ventilation	28/131 (21.4)	29/154 (18.8)	1.8	14.3	1.13 [0.67-1.90]
**Subtotal, with stratified analyses**	**59/533 (11.1)**	**77/518 (14.9)**	**-6.4**	**32.1**	**0.82 [0.58-1.16]**
**Canada, after Solidarity (in-hospital mortality; controls not given placebo): trend p = 0.75**					
No oxygen	3/11 (27.3)	1/10 (10.0)	0.9	0.8	2.91 [0.35-24.42]
Low-flow oxygen	10/96 (10.4)	11/89 (12.4)	-0.9	4.7	0.83 [0.33-2.04]
High-flow oxygen or non-invasive ventilation	14/49 (28.6)	13/47 (27.7)	0.2	4.9	1.05 [0.43-2.53]
Invasive ventilation	4/14 (28.6)	2/7 (28.6)	0.0	1.0	1.00 [0.14-7.10]
**Subtotal, with stratified analyses**	**31/170 (18.2)**	**27/153 (17.6)**	**0.2**	**11.4**	**1.02 [0.57-1.82]**
**Other trials (28-day mortality; only Wuhan was placebo-controlled): trend p = 0.55**					
Wuhan: (a) low-flow oxygen	11/129 (8.5)	(7/68) x2† (10.3)	-0.8	3.7	0.81 [0.29-2.23]
(b) high-flow oxygen or ventilation	11/29 (37.9)	(3/10) x2† (30.0)	0.6	1.8	1.40 [0.32-6.01]
SIMPLE: no oxygen	5/384 (1.3)	(4/200) x2† (2.0)	-0.9	2.0	0.64 [0.16-2.55]
Amritsar: no ventilation	6/41 (14.6)	5/41 (12.2)	0.5	2.4	1.23 [0.35-4.35]
Egypt: mild/moderate	9/100 (9.0)	7/100 (7.0)	1.0	3.7	1.31 [0.47-3.63]
**Subtotal, with stratified analyses**	**42/683 (6.1)**	**40/697† (5.7)**	**0.4**	**13.6**	**1.03 [0.61-1.75]**
**Summation of relevant strata from all other trials except Solidarity: trend p = 0.23**					
No oxygen	11/470 (2.3)	12/473† (2.5)	-0.3	4.4	0.93 [0.37-2.39]
Low-flow oxygen	45/598 (7.5)	62/569† (10.9)	-8.2	21.1	0.68 [0.44-1.04]
High-flow oxygen or ventilation	76/318 (23.9)	70/326† (21.5)	2.8	31.6	1.09 [0.77-1.55]
**Stratified total**	**132/1386 (9.5)**	**144/1368† (10.5)**	**-5.7**	**57.1**	**0.90 [0.70-1.17]**
**Meta-analysis (by summation of relevant strata from Solidarity and all other trials): trend p = 0.02**					
No oxygen	36/1339 (2.7)	45/1334† (3.4)	-4.2	18.8	0.80 [0.51-1.26]
On oxygen, no ventilation; includes high-flow oxygen only in Solidarity*	471/3516 (13.4)	538/3490† (15.4)	-40.2	244.6	0.85 [0.75-0.96]
Ventilation; includes high-flow oxygen in studies other than Solidarity	227/677 (33.5)	204/673† (30.3)	11.0	101.4	1.11 [0.92-1.35]
**Total (all trials), with stratified analyses**	**734/5532 (13.3)**	**787/5497† (14.3)**	**-33.4**	**364.8**	**0.91 [0.82-1.01] p = 0.08**

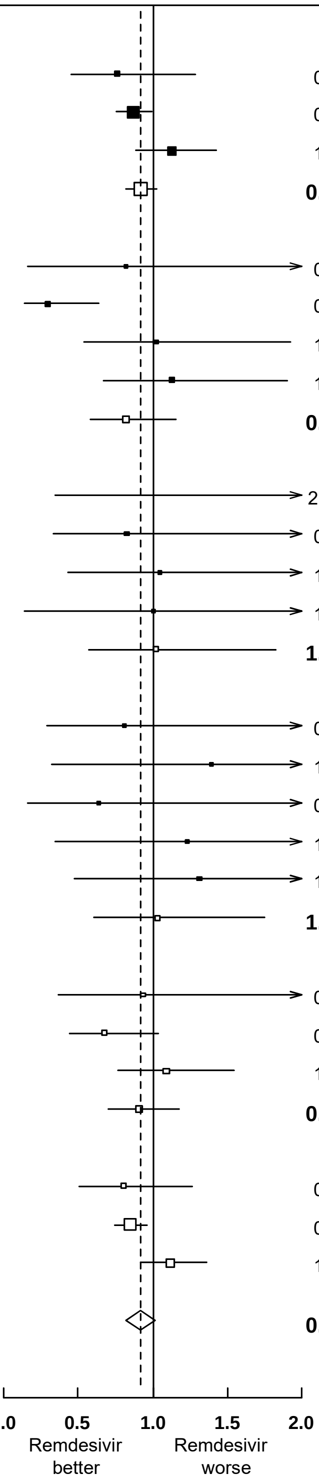

* High-flow and low-flow oxygen were not recorded separately at entry into Solidarity

** Age-stratified log-rank O-E for Solidarity, O-E from 2x2 tables for small trials, and w.loge HR for ACTT-1 strata (with the weight w being the inverse of the variance of loge HR, obtained from the 95% CI for the Hazard Ratio). If V is the variance of the logrank statistic O-E then RR is got from taking $\log_e$ RR to be (O-E)/V with Normal variance 1/V. Summation of (O-E) and V yields the stratified total (ie, the inverse-variance-weighted average of the separate $\log_e$ RR values).

‡ After Solidarity ended, its Canadian centres continued randomising remdesivir vs its control until 1 April, 2021.

† For balance, in studies with only half as many allocated control as remdesivir the controls count twice in total deaths/patients.

Figure S17B. Hydroxychloroquine vs its control in hospitalised COVID – Meta-analysis of mortality in Solidarity, Recovery, & the other RCTs with some deaths

Solidarity includes in-hospital deaths before or after day 28, but Recovery and some other trials stop at day 28. Statistical analyses (O-E, RR, etc) are stratified for respiratory support, but the overall % is not. The French, Canadian and Norwegian results, published separately, are not additional to this.

Trial, and respiratory support at the time of randomisation	Deaths reported / No. of patients (%)		Hydroxychlor. deaths: Observed-Expected		Death rate ratio RR, and 95% CI
	Hydroxychlor.	Control	(O-E)*	Var (O-E)	Hydroxychlor. : Control
**Solidarity (stratified by oxygen use and by ventilation at entry)**					
No oxygen	8/346 (2.3)	6/338 (1.8)	0.9	3.5	1.29 [0.45-3.69]
Low-flow or high-flow O_2	61/518 (11.8)	55/480 (11.5)	0.9	28.8	1.03 [0.72-1.49]
Ventilation (any type) or ECMO	35/84 (41.7)	28/82 (34.1)	3.4	15.0	1.25 [0.76-2.08]
**Stratified total: Solidarity**	**104/948 (11.0)**	**89/900 (9.9)**	**5.2**	**47.3**	**1.12 [0.84-1.48]**
**Recovery (stratified by oxygen use and by ventilation at entry)**					
No oxygen	58/362 (16.0)	(99/750) /2† (13.2)	7.3	34.1	1.24 [0.89-1.73]
O_2 or non-invasive ventilation	253/938 (27.0)	(475/1873) /2† (25.4)	12.4	157.9	1.08 [0.93-1.26]
Invasive ventilation or ECMO	110/261 (42.1)	(216/532) /2† (40.6)	1.9	70.6	1.03 [0.81-1.30]
**Stratified total: Recovery**	**421/1561 (27.0)**	**(790/3155) /2† (25.0)**	**21.7**	**262.7**	**1.09 [0.96-1.23]**
**Solidarity and Recovery**	**525/2509 (20.9)**	**484/2478 (19.5)**	**26.9**	**309.9**	**1.09 [0.98-1.22]**
**18 small trials**	**148/1452 (10.2)**	**161/1472 (10.9)**	**-3.5**	**58.1**	**0.94 [0.73-1.22]**
**Stratified total**	**673/3961 (17.0)**	**645/3950 (16.3)**	**23.4**	**368.0**	**1.07 [0.96-1.18]**

Heterogeneity between Solidarity, Recovery & 18 small trials: $\chi^2_2 = 1.1$; p = 0.58

p = 0.22

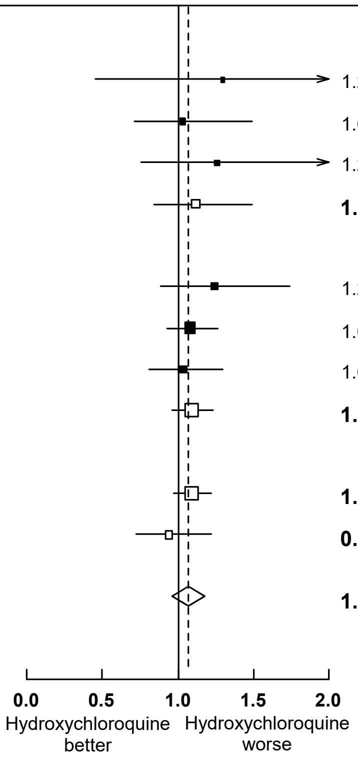

* Log-rank O-E for Solidarity and Recovery, and sum of O-E from 2x2 tables for small trials.

RR is got by taking $\log_e$RR to be (O-E)/V with Normal variance 1/V. Similar use of subtotals or of totals of (O-E) and of V yield inverse-variance-weighted averages of the $\log_e$RR values.

† For balance, only half the control numbers in Recovery are added into totals and subtotals

Figure S17C. Lopinavir vs its control in hospitalised COVID – Meta-analysis of mortality in Solidarity, Recovery, and the other RCT with some deaths

Solidarity includes in-hospital deaths before or after day 28, but the other trials stop at day 28.
Statistical analyses (O-E, RR, etc) are stratified for respiratory support, but the overall % is not.
The French, Canadian and Norwegian results, published separately, are not additional to this.

Trial, and respiratory support at the time of randomisation	Deaths reported / No. of patients (%): Lopinavir	Deaths reported / No. of patients (%): Control	Lopinavir deaths: Observed-Expected (O-E)*	Var (O-E)	Death rate ratio RR, and 95% CI (Lopinavir : Control)
**Solidarity (stratified by oxygen use and by ventilation at entry)**					
No oxygen	21/527 (4.0)	17/535 (3.2)	2.5	9.4	1.31 [0.69-2.48]
Low-flow or high-flow O_2	96/765 (12.5)	100/718 (13.9)	-7.8	48.3	0.85 [0.64-1.13]
Ventilation (any type) or ECMO	34/112 (30.4)	36/115 (31.3)	0.6	16.7	1.04 [0.64-1.68]
**Stratified total: Solidarity**	**151/1404 (10.8)**	**153/1368 (11.2)**	**-4.7**	**74.5**	**0.94 [0.75-1.18]**
**Recovery (stratified by oxygen use and by ventilation at entry)**					
No oxygen	71/425 (16.7)	(126/896) /2† (14.1)	8.2	42.5	1.21 [0.90-1.64]
O_2 or non-invasive ventilation	279/1131 (24.7)	(586/2384) /2† (24.6)	-0.4	185.9	1.00 [0.86-1.15]
Invasive ventilation or ECMO	24/60 (40.0)	(55/144) /2† (38.2)	1.6	15.6	1.11 [0.67-1.82]
**Stratified total: Recovery**	**374/1616 (23.1)**	**(767/3424) /2† (22.4)**	**9.4**	**244.0**	**1.04 [0.92-1.18]**
**Solidarity and Recovery**	**525/3020 (17.4)**	**537/3080 (17.4)**	**4.7**	**318.5**	**1.01 [0.91-1.13]**
**Wuhan trial**	**19/99 (19.2)**	**25/100 (25.0)**	**-2.9**	**8.6**	**0.71 [0.37-1.39]**
**Stratified total**	**544/3119 (17.4)**	**562/3180 (17.7)**	**1.8**	**327.2**	**1.01 [0.90-1.12]**

Heterogeneity between Solidarity, Recovery & Wuhan trials: $\chi^2_2 = 1.6$; p = 0.45

p = 0.92

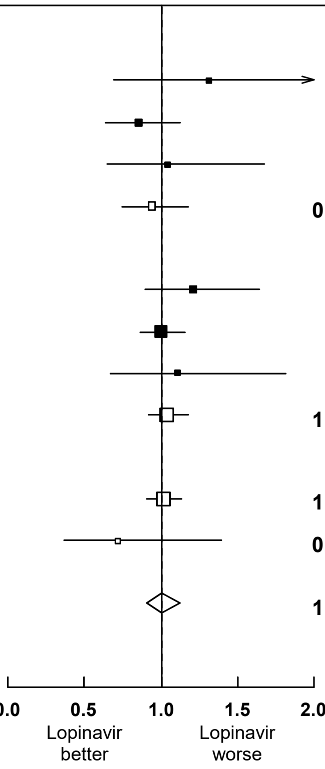

* Log-rank O-E for Solidarity and Recovery, and O-E from a 2x2 table for the Wuhan trial.

RR is got by taking $\log_e$RR to be (O-E)/V with Normal variance 1/V. Similar use of subtotals or of totals of (O-E) and of V yield inverse-variance-weighted averages of the $\log_e$RR values.

† For balance, only half the control numbers in Recovery are added into totals and subtotals.

Figure S17D. Interferon-β vs its control in hospitalised COVID – Meta-analysis of mortality in Solidarity, ACTT-3, and the other RCTs with some deaths

Solidarity includes in-hospital deaths before or after day 28, but other trials may stop at day 28.
Statistical analyses (O-E, RR etc) are stratified for respiratory support, but the overall % is not.
The French, Canadian and Norwegian results, published separately, are not additional to this.

Trial, and respiratory support at the time of randomisation	Deaths reported / No. of patients (%): Interferon	Control	Interferon deaths: Observed-Expected (O-E)*	Var (O-E)	Death rate ratio RR, and 95% CI (Interferon : Control)
**Solidarity (in-hospital mortality; controls not given placebo)**					
No oxygen	12/503 (2.4)	16/508 (3.1)	-1.8	7.0	0.78 [0.37-1.63]
Low-flow or high-flow O_2	235/1497 (15.7)	199/1503 (13.2)	18.9	107.6	1.19 [0.99-1.44]
Ventilation (any type) or ECMO	69/144 (47.9)	51/136 (37.5)	10.0	29.3	1.41 [0.98-2.02]
Stratified total: Solidarity	316/2144 (14.7)	266/2147 (12.4)	27.1	143.9	**1.21 [1.03-1.42]**
**ACTT-3 (28-day mortality; controls given placebo)**					
No O_2	2/84 (2.4)	0/68 (0.0)	0.9	0.5	6.05 [0.38-96.72]
Low-flow O_2	12/368 (3.3)	12/380 (3.2)	0.2	6.0	1.04 [0.47-2.32]
High-flow O_2 or non-invasive ventilation	7/35 (20.0)	4/34 (11.8)	1.4	2.6	1.74 [0.51-5.93]
Stratified subtotal	21/487 (4.3)	16/482 (3.3)	2.5	9.0	**1.33 [0.69-2.54]**
**3 small trials (unstratified†)**	**24/126 (19.0)**	**39/126 (31.0)**	**-6.2**	**9.6**	**0.53 [0.28-0.99]**
**Stratified total**	**361/2757 (13.1)**	**321/2755 (11.7)**	**23.5**	**162.5**	**1.16 [0.99-1.35]**

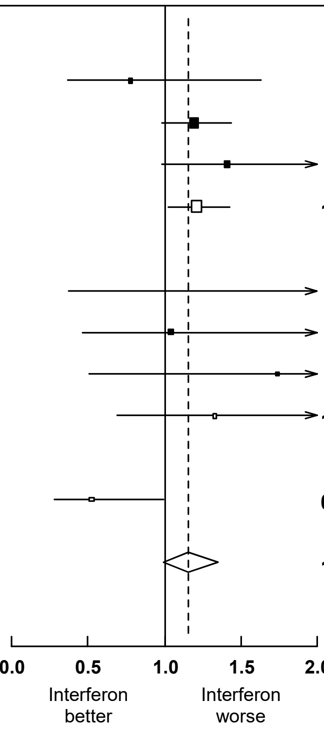

Heterogeneity between Solidarity, ACTT-3 & small trials: $\chi^2_2 = 6.4$; p = 0.04

p = 0.07

* Log-rank O-E for Solidarity, and sum of O-E from 2x2 tables for small trials.

Note that the multivariate analysis of Solidarity in Table S3 yields RR=1.15 (p=0.06) for interferon instead of RR=1.21 (p=0.02), as in the pre-specified pairwise Kaplan-Meier and log-rank analyses. The reduction in RR on going from pairwise to multivariate analysis shows the apparently adverse effect of interferon in Solidarity was at least partly due to an adverse play of chance at randomisation.

RR is got by taking logeRR to be (O-E)/V with Normal variance 1/V. Similar use of subtotals or of totals of (O-E) and of V yields inverse-variance-weighted averages of the logeRR values.

† All 3 small trials were single-centre Iranian studies. For balance, in the one trial in which half as many were allocated control as interferon the control results are counted twice in the total number of deaths and patients. In another, 4 early deaths that were omitted as they prevented interferon completion have been restored.
